# Supplementary material for: Simulation of Solvatochromic Phenomena in Xanthione Using Explicit Solvent Methods
Source: Molecules. 2024 Nov 27;29(23):5609. doi: 10.3390/molecules29235609 (PMC11643922; doi:10.3390/molecules29235609)
Supplement: Supplementary file 1 [file molecules-29-05609-s001.zip › molecules-3292532-supplementary.pdf]

Electronic supplementary information: Simulation of solvatochromic phenomena in xanthione using explicit solvent methods

Anjay Manian,[1, a)] Zifei Chen,[2] Rohan J. Hudson,[3,4] and Salvy P. Russo[1, b)]

[1)] *ARC Centre of Excellence in Exciton Science, School of Science, RMIT University, Melbourne, 3000, Australia.*

[2)] *ARC Centre of Excellence in Exciton Science, School of Chemistry, The University of Melbourne, Parkville, 3010, Australia.*

[3)] *MOGLabs, Carlton, 3053, Australia*

[4)] *ARC Centre of Excellence in Exciton Science, School of Chemistry, University of Melbourne, Parkville, 3010, Australia.*

(Dated: 12 November 2024)

Electronic supplementary information contains all absolute energies (hartree) for xanthione in each solvent for implicit and explicit solvent configurations, rendered frontier molecular orbitals, and the optimised coordinates for each configurations studied in this work.

[a)] Electronic mail: anjay.manian3@rmit.edu.au

[b)] Electronic mail: salvy.russo@rmit.edu.au

Xanthione

CONTENTS

I. Energies S7
A. Vertical excitation energies S7

II. Molecular orbitals S8

III. Optimised Geometries - Acetone S16
A. Implicit S16
1. S_0 S16
2. L_a S17
3. L_b S19
B. S=2 S21
1. S_0 S21
2. L_a S23
3. L_b S25
C. S=4 S28
1. S_0 S28
2. L_a S31
3. L_b S34

IV. Optimised Geometries - Acetonitrile S37
A. Implicit S37
1. S_0 S37
2. L_a S39
3. L_b S40
B. S=2 S42
1. S_0 S42
2. L_a S44
3. L_b S46
C. S=4 S48
1. S_0 S48
2. L_a S51

Xanthione

3. L_b S53

D. S=6 S56

1. S_0 S56

2. L_a S59

3. L_b S62

V. Optimised Geometries - Dichloroethane S65

A. Implicit S65

1. S_0 S65

2. L_a S66

3. L_b S68

B. S=2 S70

1. S_0 S70

2. L_a S72

3. L_b S74

C. S=4 S77

1. S_0 S77

2. L_a S79

3. L_b S82

D. S=6 S85

1. S_0 S85

2. L_a S88

3. L_b S91

VI. Optimised Geometries - Dimethyl sulfoxide S95

A. Implicit S95

1. S_0 S95

2. L_a S96

3. L_b S98

B. S=2 S100

1. S_0 S100

2. L_a S102

3. L_b S105

Xanthione

C. S=4 S107

1. S_0 S107

2. L_a S110

3. L_b S113

D. S=6 S116

1. S_0 S116

2. L_a S120

3. L_b S123

VII. Optimised Geometries - Formamide S127

A. Implicit S127

1. S_0 S127

2. L_a S129

3. L_b S130

B. S=2 S132

1. S_0 S132

2. L_a S134

3. L_b S136

C. S=4 S138

1. S_0 S138

2. L_a S141

3. L_b S143

D. S=6 S146

1. S_0 S146

2. L_a S149

3. L_b S152

VIII. Optimised Geometries - Formic acid S155

A. Implicit S155

1. S_0 S155

2. L_a S156

3. L_b S158

B. S=2 S160

Xanthione

1. S_0 S160

2. L_a S162

3. L_b S164

C. S=4 S166

1. S_0 S166

2. L_a S168

3. L_b S171

D. S=6 S173

1. S_0 S173

2. L_a S176

3. L_b S178

IX. Optimised Geometries - Toluene S181

A. Implicit S181

1. S_0 S181

2. L_a S183

3. L_b S185

B. S=2 S186

1. S_0 S186

2. L_a S189

3. L_b S192

X. Optimised Geometries - Water S194

A. Implicit S194

1. S_0 S194

2. L_a S196

3. L_b S198

B. S=2 S200

1. S_0 S200

2. L_a S202

3. L_b S203

C. S=4 S205

1. S_0 S205

Xanthione

2. L_a S208

3. L_b S210

D. S=6 S212

1. S_0 S212

2. L_a S214

3. L_b S216

XI. Optimised Geometries - Gas phase S219

A. Implicit S219

1. S_0 S219

2. L_a S220

3. L_b S222

Xanthione

TABLE S1. Absolute vertical excitation energies of xanthione for all studied solvents in each implict and explicit solvent configuration. Energies are in atomic units.

IMPLICIT			S=2			S=4			S=6		
S_0	L_a	L_b	S_0	L_a	L_b	S_0	L_a	L_b	S_0	L_a	L_b
Acetone (ε=20.493)											
-1398.642893	-1398.640036	-1398.638971	-1784.867534	-1784.859463	-1784.86042	-2171.159146	-2171.152629	-2171.152243	-	-	-
-1398.549580	-1398.552947	-1398.549701	-1784.756165	-1784.792881	-1784.789285	-2171.069309	-2171.070021	-2171.064731	-	-	-
-1398.537860	-1398.537087	-1398.539223	-1784.766528	-1784.766042	-1784.766908	-2171.061572	-2171.058636	-2171.059833	-	-	-
Acetonitrile (ε=35.688)											
-1398.643806	-1398.640944	-1398.640091	-1664.107599	-1664.102476	-1664.1023	-1929.58421	-1929.579542	-1929.575019	-2195.045564	-2195.03821	-2195.044457
-1398.549775	-1398.553024	-1398.549677	-1664.01694	-1664.018963	-1664.014454	-1929.493745	-1929.496246	-1929.485915	-2194.954951	-2194.952487	-2194.953734
-1398.539465	-1398.538619	-1398.540425	-1664.008124	-1664.004194	-1664.008195	-1929.484484	-1929.48175	-1929.480613	-2194.943727	-2194.938169	-2194.949535
Dichloroethane (ε=10.125)											
-1398.644108	-1398.641251	-1398.640467	-2504.974756	-2504.968362	-2504.968009	-3611.308827	-3611.305009	-3611.303934	-7392.832947	-7392.825162	-7392.822846
-1398.549833	-1398.553034	-1398.54968	-2504.883431	-2504.883914	-2504.878498	-3611.216514	-3611.239523	-3611.213803	-7392.728203	-7392.730974	-7392.73186
-1398.539993	-1398.539131	-1398.540868	-2504.876225	-2504.871008	-2504.872943	-3611.209656	-3611.212005	-3611.209047	-7392.730634	-7392.722623	-7392.74277
Dimythylsulfoxide (ε=46.826)											
-1398.640881	-1398.637989	-1398.636516	-3396.678433	-3396.670871	-3396.669523	-5394.75107	-5394.743445	-5394.743119	-7392.832947	-7392.825162	-7392.822846
-1398.549087	-1398.552755	-1398.549818	-3396.604847	-3396.605638	-3396.603012	-5394.638503	-5394.678278	-5394.674081	-7392.746993	-7392.746806	-7392.74277
-1398.534315	-1398.533659	-1398.537182	-3396.576448	-3396.577578	-3396.578457	-5394.649718	-5394.651026	-5394.65098	-7392.728203	-7392.730974	-7392.73186
Formamide (ε=108.940)											
-1398.644668	-1398.6418	-1398.641154	-1738.409152	-1738.394279	-1738.402564	-2078.183578	-2078.167859	-2078.176415	-2417.947934	-2417.941503	-2417.942883
-1398.549933	-1398.553071	-1398.549662	-1738.318657	-1738.322981	-1738.314205	-2078.093551	-2078.095928	-2078.08763	-2417.858424	-2417.858772	-2417.852288
-1398.540981	-1398.540049	-1398.541521	-1738.317116	-1738.309979	-1738.315032	-2078.092201	-2078.085194	-2078.086053	-2417.85532	-2417.849392	-2417.852016
Formic acid (ε=51.100)											
-1398.644213	-1398.641359	-1398.640667	-1999.474364	-1999.466807	-1999.465395	-2600.319465	-2600.311853	-2600.312284	-3201.175849	-3201.16808	-3201.168587
-1398.54986	-1398.553021	-1398.549695	-1999.382882	-1999.385902	-1999.37927	-2600.227546	-2600.230029	-2600.22425	-3201.083695	-3201.086162	-3201.082494
-1398.539609	-1398.538764	-1398.540605	-1999.374496	-1999.369154	-1999.372711	-2600.217657	-2600.213936	-2600.213387	-3201.071737	-3201.068104	-3201.070274
Toluene (ε=2.374)											
-1398.631734	-1398.628276	-1398.624625	-1941.662191	-1941.654962	-1941.646849	-	-	-	-	-	-
-1398.54567	-1398.551004	-1398.553111	-1941.579034	-1941.583521	-1941.574334	-	-	-	-	-	-
-1398.521019	-1398.526821	-1398.529989	-1941.562049	-1941.564222	-1941.568663	-	-	-	-	-	-
Water (ε=78.355)											
-1398.643031	-1398.640181	-1398.63927	-1442.410738	-1442.401387	-1442.398971	-1486.178175	-1486.17055	-1486.169141	-1529.945093	-1529.936984	-1529.934796
-1398.549754	-1398.552997	-1398.549695	-1442.324143	-1442.325136	-1442.319498	-1486.091919	-1486.093714	-1486.08535	-1529.858556	-1529.858708	-1529.854649
-1398.53913	-1398.538338	-1398.540428	-1442.315947	-1442.310299	-1442.31353	-1486.083319	-1486.079704	-1486.079016	-1529.848617	-1529.84447	-1529.847579
Gas phase											
-1398.622981	-1398.619035	-1398.613404									
-1398.503809	-1398.547795	-1398.547242									
-1398.510062	-1398.518137	-1398.522826									

I. ENERGIES

A. Vertical excitation energies

Table S1 shows the absolute energies for each excited state of interest in xanthione for all studied solvents in each implict and explicit solvent configuration. From this data can be calculated the vertical excitation energies.

Xanthione

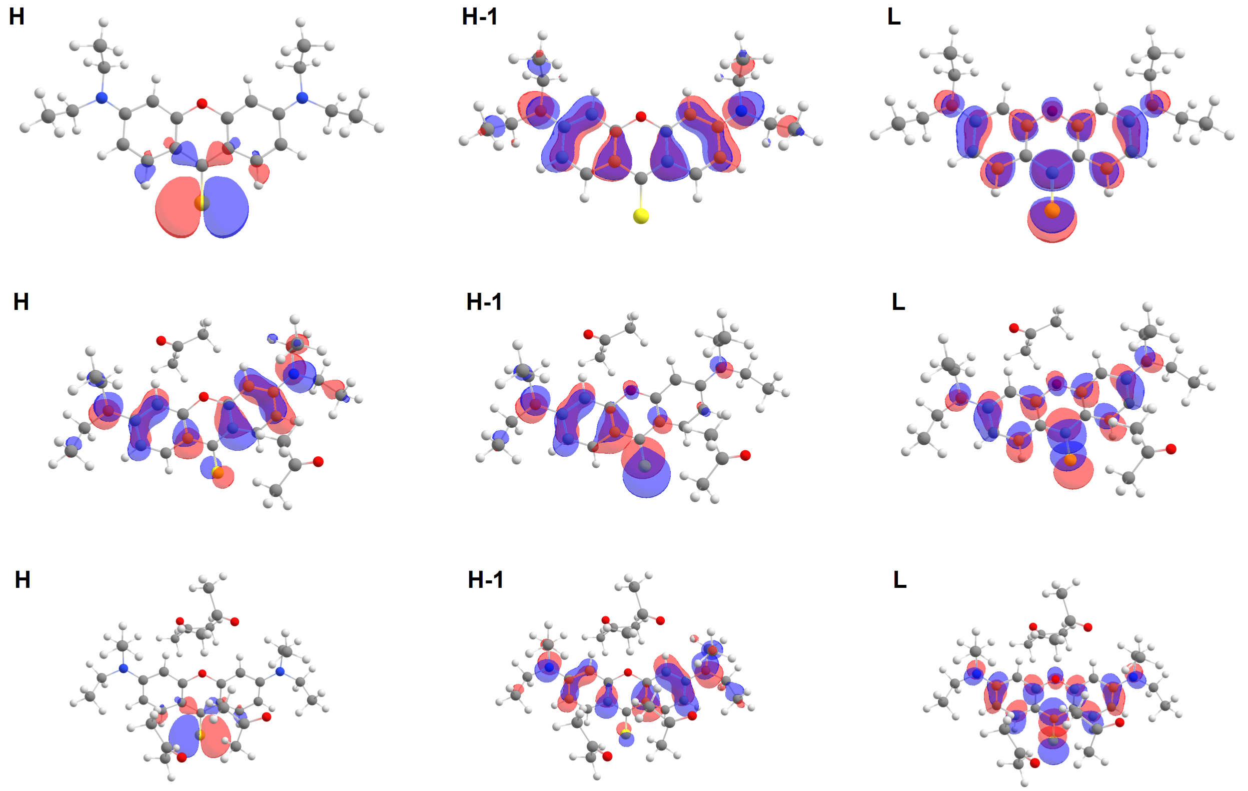

FIG. S1. Visualised frontier molecular orbitals for xanthione in acetone. Top row is the implicit solvent, and each preceding row is for explicit solvent of increasing values. **H** and **L** here refer to the highest occupied and lowest unoccupied molecular orbitals, respectively.

II. MOLECULAR ORBITALS

The frontier molecular orbitals for xanthione in each implicit and explicit solvent configurations can be observed in Figures S1- S9.

Xanthione

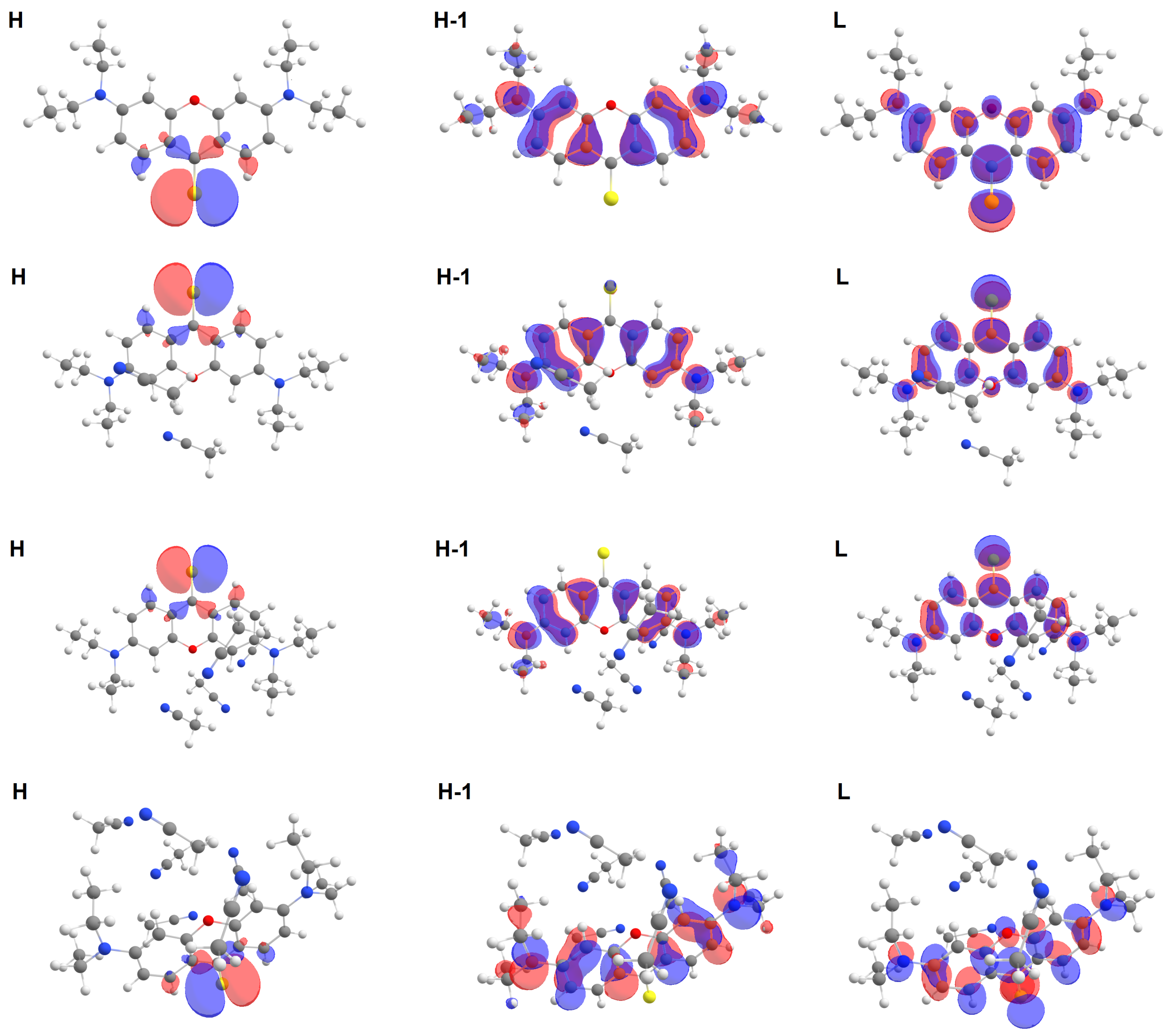

FIG. S2. Visualised frontier molecular orbitals for xanthione in acetonitrile. Top row is the implicit solvent, and each preceding row is for explicit solvent of increasing values. **H** and **L** here refer to the highest occupied and lowest unoccupied molecular orbitals, respectively.

.

Xanthione

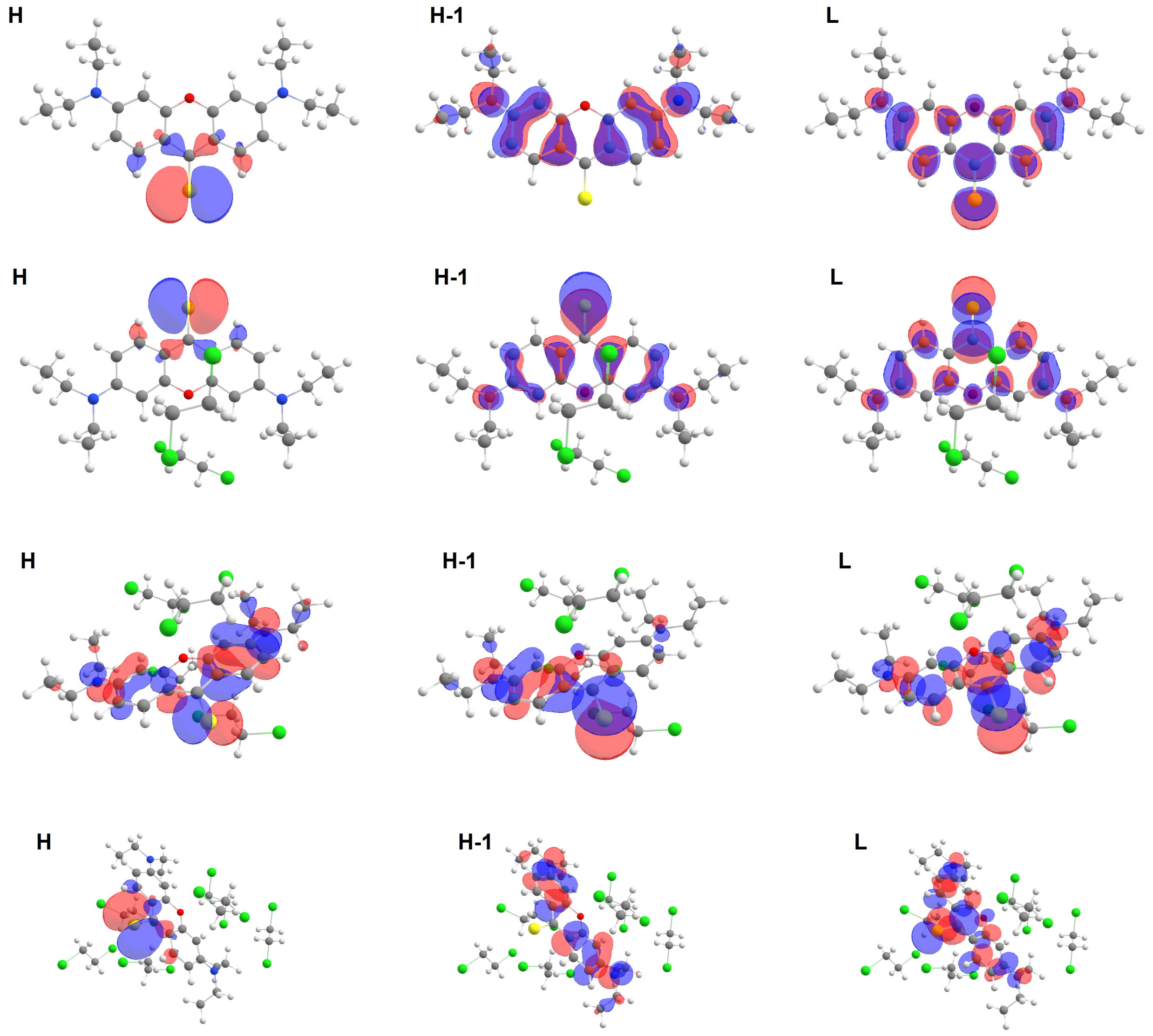

FIG. S3. Visualised frontier molecular orbitals for xanthione in dichloroethane. Top row is the implicit solvent, and each preceding row is for explicit solvent of increasing values. **H** and **L** here refer to the highest occupied and lowest unoccupied molecular orbitals, respectively.

.

Xanthione

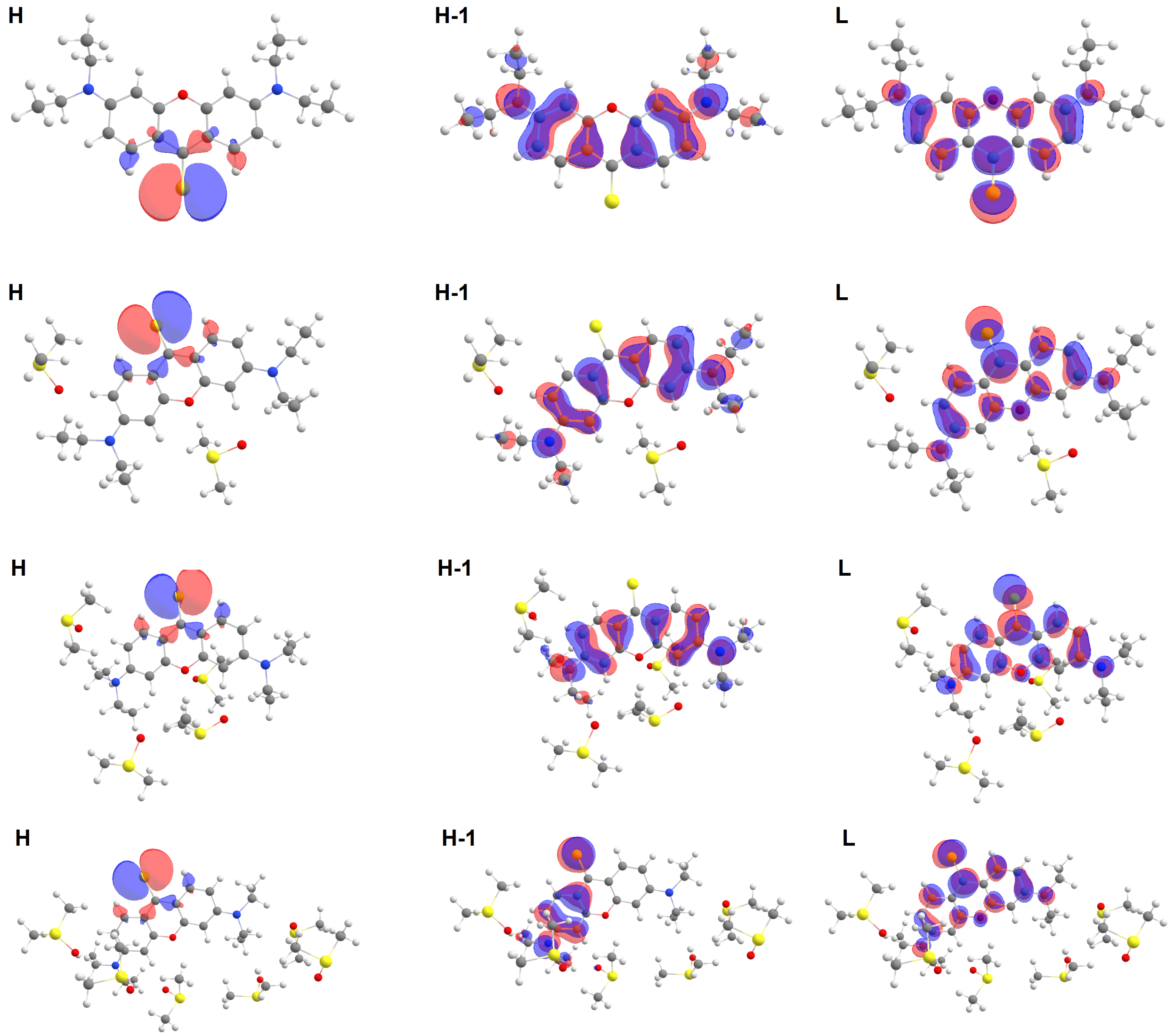

FIG. S4. Visualised frontier molecular orbitals for xanthione in dimethyl sulfoxide. Top row is the implicit solvent, and each preceding row is for explicit solvent of increasing values. **H** and **L** here refer to the highest occupied and lowest unoccupied molecular orbitals, respectively.

.

Xanthione

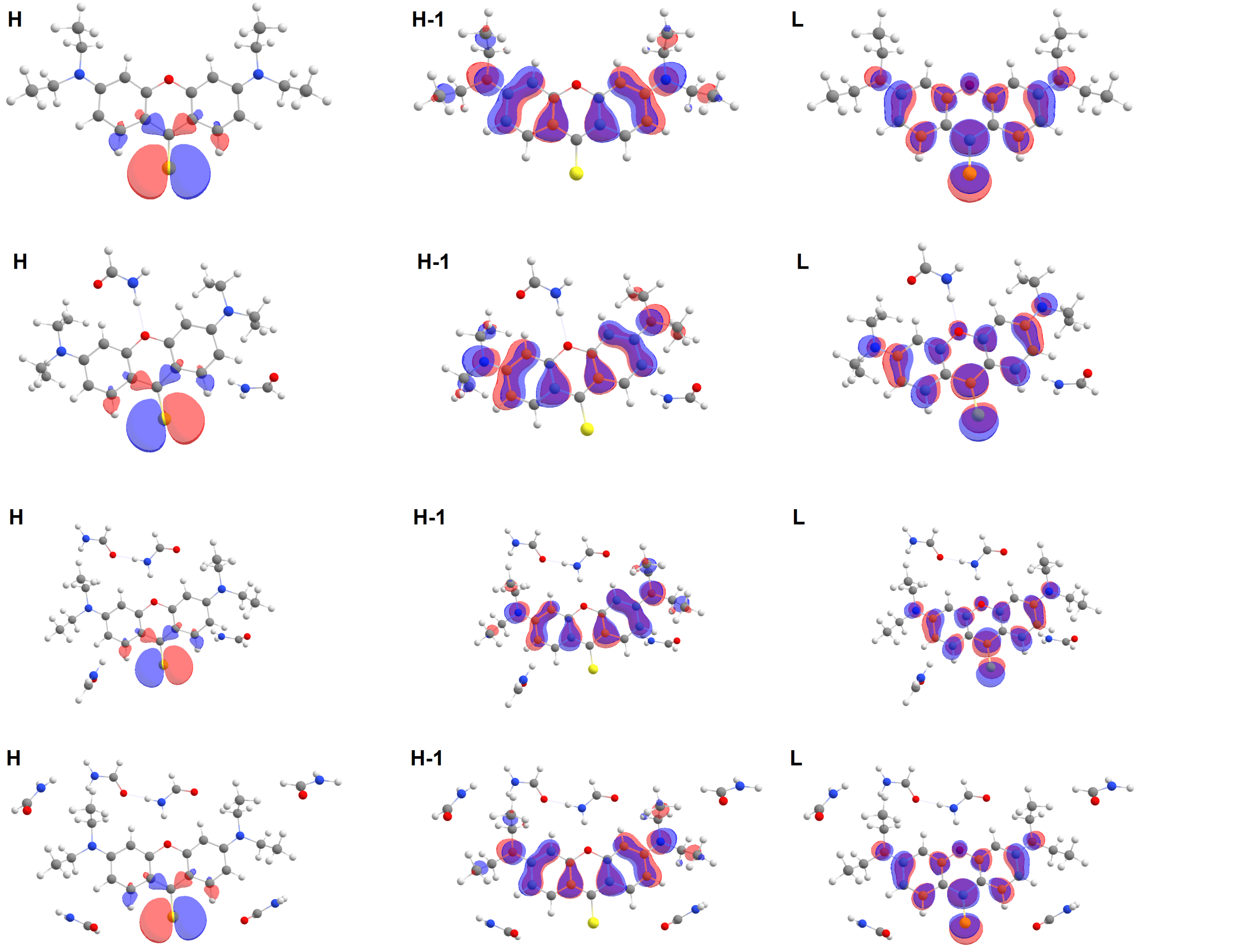

FIG. S5. Visualised frontier molecular orbitals for xanthione in formamide. Top row is the implicit solvent, and each preceding row is for explicit solvent of increasing values. **H** and **L** here refer to the highest occupied and lowest unoccupied molecular orbitals, respectively.

.

Xanthione

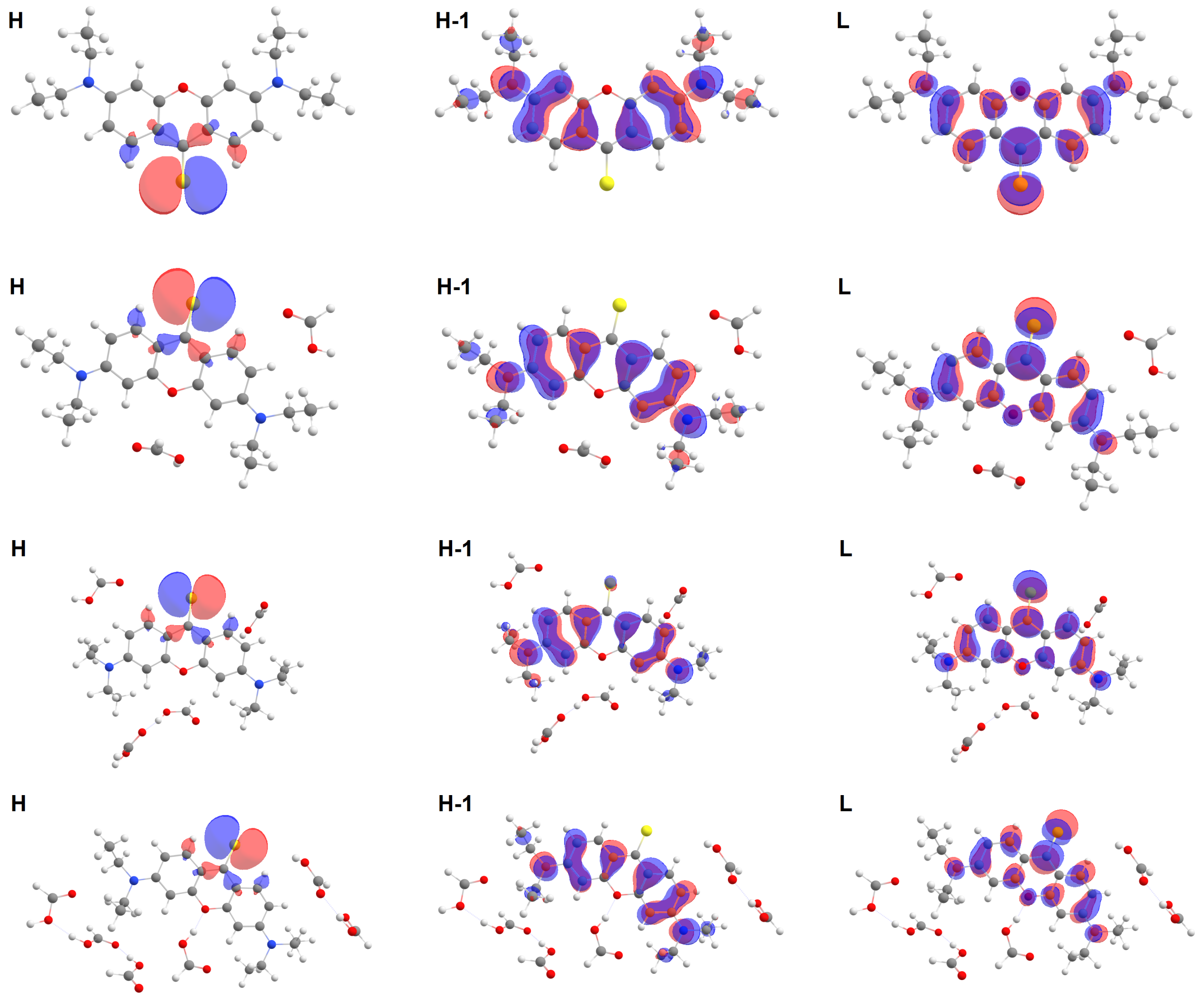

FIG. S6. Visualised frontier molecular orbitals for xanthione in formic acid. Top row is the implicit solvent, and each preceding row is for explicit solvent of increasing values. **H** and **L** here refer to the highest occupied and lowest unoccupied molecular orbitals, respectively.

.

Xanthione

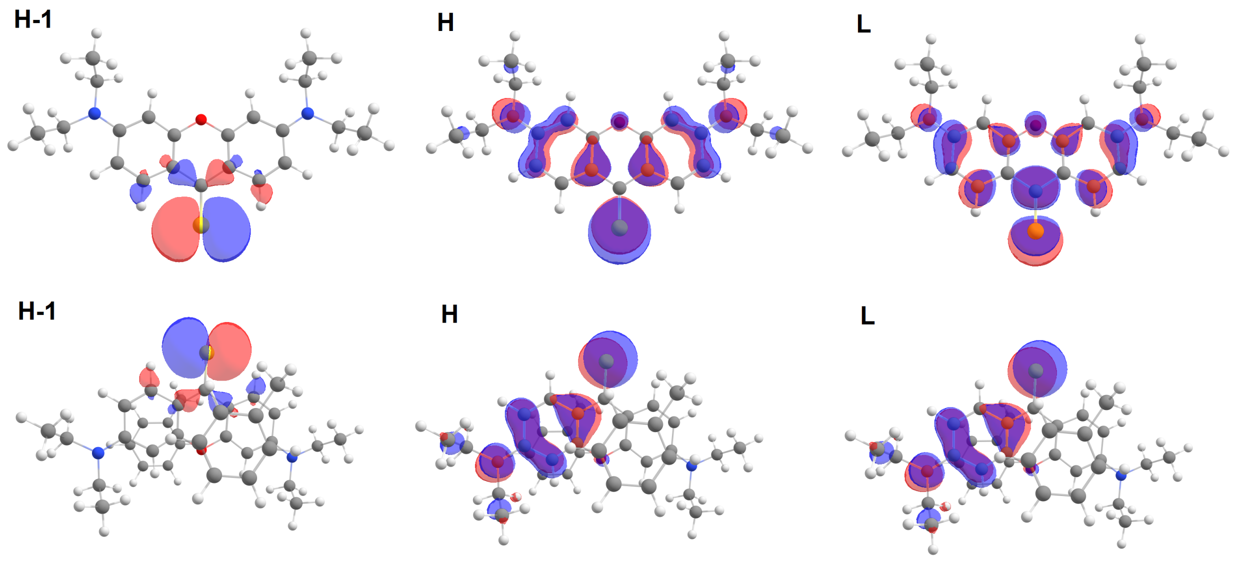

FIG. S7. Visualised frontier molecular orbitals for xanthione in toluene. Top row is the implicit solvent, and each preceding row is for explicit solvent of increasing values. Top row is the implicit solvent, and each preceding row is for explicit solvent of increasing values. **H** and **L** here refer to the highest occupied and lowest unoccupied molecular orbitals, respectively.

.

Xanthione

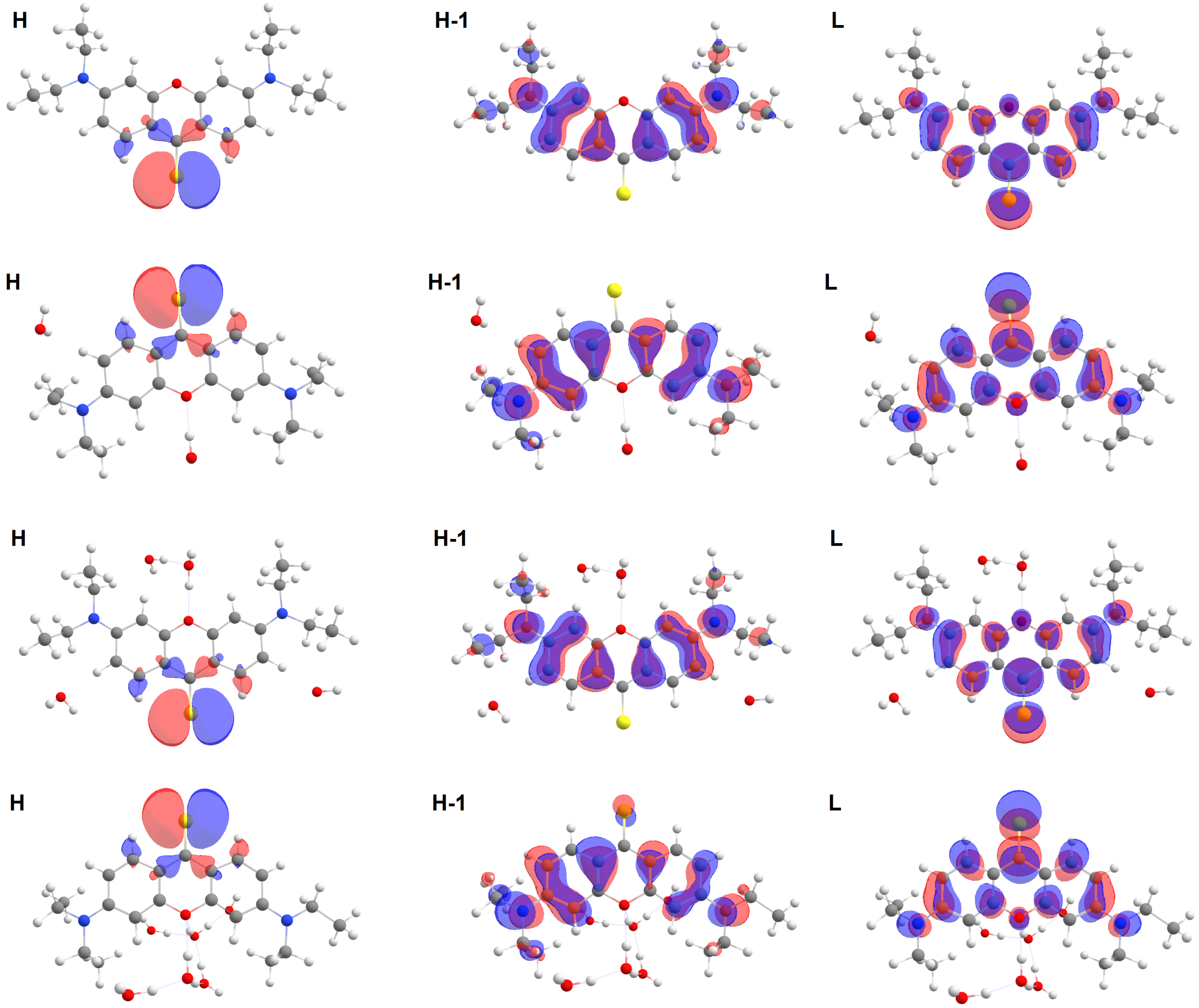

FIG. S8. Visualised frontier molecular orbitals for xanthione in water. Top row is the implicit solvent, and each preceding row is for explicit solvent of increasing values. **H** and **L** here refer to the highest occupied and lowest unoccupied molecular orbitals, respectively.

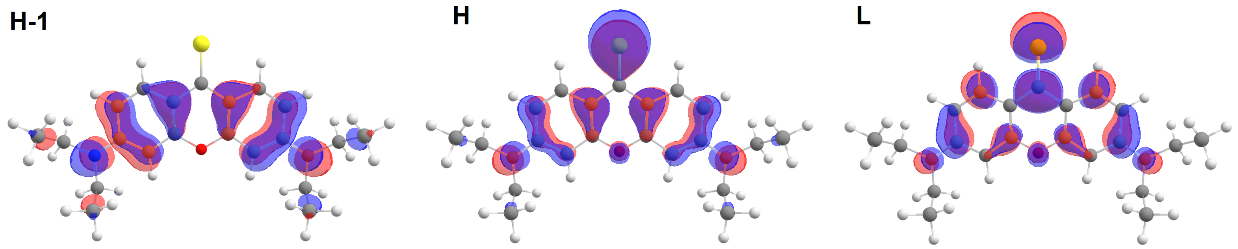

FIG. S9. Visualised frontier molecular orbitals for xanthione in the gas phase. **H** and **L** here refer to the highest occupied and lowest unoccupied molecular orbitals, respectively.

Xanthione

III. OPTIMISED GEOMETRIES - ACETONE

A. Implicit

1. S_0

6	2.308277000	-0.785941000	-0.357131000
6	3.581837000	-0.205334000	-0.293199000
6	3.639065000	1.205748000	-0.111902000
6	2.502146000	1.951813000	-0.030178000
6	1.215085000	1.387069000	-0.104976000
6	1.175799000	-0.003201000	-0.271323000
6	-0.000008000	2.153176000	-0.015703000
6	-1.215110000	1.387071000	-0.104963000
6	-1.175832000	-0.003197000	-0.271290000
6	-2.502169000	1.951811000	-0.030124000
6	-3.639093000	1.205749000	-0.111816000
6	-2.308313000	-0.785937000	-0.357074000
6	-3.581876000	-0.205338000	-0.293114000
1	-2.570716000	3.021932000	0.103542000
1	-2.166391000	-1.847998000	-0.470821000
1	-4.590442000	1.706334000	-0.035807000
1	2.166358000	-1.848004000	-0.470851000
1	2.570695000	3.021936000	0.103473000
1	4.590428000	1.706323000	-0.035951000
8	-0.000016000	-0.676840000	-0.351002000
16	-0.000009000	3.831132000	0.179614000
7	4.712799000	-0.948722000	-0.412296000
7	-4.712815000	-0.948783000	-0.412223000
6	4.642573000	-2.397234000	-0.560590000
1	3.860548000	-2.641469000	-1.280252000
1	5.579123000	-2.718644000	-1.012744000
6	4.416811000	-3.152718000	0.743262000

Xanthione

1	3.505353000	-2.823896000	1.241874000
1	5.249958000	-3.006009000	1.429569000
1	4.327134000	-4.221152000	0.542841000
6	6.034452000	-0.345899000	-0.276392000
1	6.740354000	-1.017101000	-0.762174000
1	6.064288000	0.583829000	-0.844448000
6	6.467808000	-0.101258000	1.163392000
1	6.540979000	-1.037806000	1.715015000
1	5.763567000	0.545393000	1.686458000
1	7.446657000	0.379789000	1.180329000
6	-4.642468000	-2.397326000	-0.560291000
1	-5.579115000	-2.718930000	-1.012101000
1	-3.860633000	-2.641576000	-1.280160000
6	-4.416179000	-3.152611000	0.743595000
1	-5.249156000	-3.005977000	1.430120000
1	-3.504635000	-2.823577000	1.241908000
1	-4.326365000	-4.221049000	0.543266000
6	-6.034532000	-0.346005000	-0.276651000
1	-6.064173000	0.583791000	-0.844589000
1	-6.740302000	-1.017130000	-0.762739000
6	-6.468299000	-0.101505000	1.163035000
1	-5.764188000	0.545076000	1.686367000
1	-6.541655000	-1.038095000	1.714563000
1	-7.447141000	0.379565000	1.179738000

2. L_a

6	2.322450000	-0.778658000	-0.360359000
6	3.602090000	-0.195059000	-0.305972000
6	3.657933000	1.202051000	-0.138347000
6	2.507706000	1.955690000	-0.054549000
6	1.228877000	1.387462000	-0.117131000

Xanthione

6	1.189301000	-0.006687000	-0.274342000
6	0.000001000	2.111327000	-0.022666000
6	-1.228862000	1.387434000	-0.117085000
6	-1.189253000	-0.006726000	-0.274272000
6	-2.507695000	1.955632000	-0.054469000
6	-3.657918000	1.201962000	-0.138206000
6	-2.322385000	-0.778712000	-0.360259000
6	-3.602047000	-0.195140000	-0.305819000
1	-2.601427000	3.027924000	0.068161000
1	-2.185650000	-1.842618000	-0.468850000
1	-4.606559000	1.709796000	-0.072938000
1	2.185710000	-1.842571000	-0.468875000
1	2.601414000	3.027984000	0.068077000
1	4.606573000	1.709897000	-0.073154000
8	0.000031000	-0.683578000	-0.340476000
16	-0.000014000	3.813897000	0.244299000
7	4.737357000	-0.955241000	-0.433072000
7	-4.737274000	-0.955401000	-0.432807000
6	4.658543000	-2.403595000	-0.536159000
1	3.876530000	-2.668996000	-1.249391000
1	5.593841000	-2.745131000	-0.977667000
6	4.426837000	-3.125946000	0.786760000
1	3.517235000	-2.777652000	1.275754000
1	5.260037000	-2.966561000	1.470451000
1	4.329740000	-4.199113000	0.615327000
6	6.054966000	-0.353973000	-0.300812000
1	6.764151000	-1.028480000	-0.778844000
1	6.085260000	0.571298000	-0.878196000
6	6.493734000	-0.088528000	1.135228000
1	6.580675000	-1.018448000	1.696325000
1	5.783275000	0.554345000	1.654545000
1	7.466889000	0.404858000	1.144258000

Xanthione

6	-4.658398000	-2.403781000	-0.535524000
1	-5.593795000	-2.745478000	-0.976686000
1	-3.876547000	-2.669387000	-1.248872000
6	-4.426351000	-3.125746000	0.787547000
1	-5.259375000	-2.966160000	1.471404000
1	-3.516624000	-2.777318000	1.276209000
1	-4.329297000	-4.198961000	0.616391000
6	-6.054938000	-0.354066000	-0.301403000
1	-6.084790000	0.571194000	-0.878818000
1	-6.763866000	-1.028533000	-0.779878000
6	-6.494585000	-0.088571000	1.134360000
1	-5.784355000	0.554206000	1.654111000
1	-6.581994000	-1.018481000	1.695404000
1	-7.467684000	0.404936000	1.142796000

3. L_b

6	2.324753000	-0.778188000	-0.370714000
6	3.601884000	-0.191659000	-0.299063000
6	3.647795000	1.207260000	-0.116564000
6	2.497274000	1.954663000	-0.035104000
6	1.216269000	1.387126000	-0.114783000
6	1.194056000	-0.003712000	-0.287026000
6	-0.008686000	2.126925000	-0.024244000
6	-1.216572000	1.384426000	-0.108537000
6	-1.177743000	-0.034807000	-0.288465000
6	-2.517246000	1.957627000	-0.023148000
6	-3.653188000	1.205395000	-0.111161000
6	-2.299828000	-0.796302000	-0.377975000
6	-3.592948000	-0.202679000	-0.297394000
1	-2.576795000	3.025690000	0.118432000
1	-2.171030000	-1.858293000	-0.506883000

Xanthione

1	-4.606324000	1.702830000	-0.034292000
1	2.189355000	-1.840883000	-0.491383000
1	2.564677000	3.024527000	0.103004000
1	4.594705000	1.716600000	-0.035532000
8	0.005981000	-0.693007000	-0.375243000
16	-0.069859000	3.853948000	0.190605000
7	4.740040000	-0.944514000	-0.419621000
7	-4.709464000	-0.955963000	-0.403175000
6	4.668741000	-2.392390000	-0.539089000
1	3.895252000	-2.652910000	-1.263215000
1	5.610377000	-2.725313000	-0.973213000
6	4.425258000	-3.127727000	0.774563000
1	3.508448000	-2.788120000	1.256003000
1	5.249862000	-2.971131000	1.469105000
1	4.335267000	-4.199491000	0.591269000
6	6.055071000	-0.338531000	-0.276714000
1	6.768778000	-1.005314000	-0.758583000
1	6.082578000	0.592705000	-0.843996000
6	6.485105000	-0.086808000	1.164351000
1	6.571716000	-1.022128000	1.716366000
1	5.770135000	0.549132000	1.685909000
1	7.456845000	0.408937000	1.182854000
6	-4.639475000	-2.406750000	-0.564514000
1	-5.579359000	-2.720722000	-1.012337000
1	-3.859384000	-2.644338000	-1.285999000
6	-4.409833000	-3.161449000	0.740079000
1	-5.236180000	-3.010364000	1.432647000
1	-3.490274000	-2.841129000	1.228465000
1	-4.332307000	-4.228689000	0.530986000
6	-6.038223000	-0.363326000	-0.265405000
1	-6.070293000	0.564926000	-0.834357000
1	-6.738953000	-1.043765000	-0.743275000

Xanthione

6	-6.456989000	-0.119586000	1.180178000
1	-5.751970000	0.532332000	1.694415000
1	-6.524675000	-1.054851000	1.733418000
1	-7.437437000	0.356940000	1.196410000

B. S=2

1. S_0

6	-2.744114000	0.267144000	0.219463000
6	-3.907801000	-0.508309000	0.051816000
6	-3.747570000	-1.818993000	-0.496316000
6	-2.514572000	-2.279173000	-0.881472000
6	-1.339410000	-1.501184000	-0.755509000
6	-1.521380000	-0.223991000	-0.186993000
6	-0.036136000	-1.944713000	-1.168652000
6	1.045196000	-1.013070000	-0.979963000
6	0.819510000	0.245372000	-0.387377000
6	2.387688000	-1.282537000	-1.336852000
6	3.404614000	-0.392768000	-1.107132000
6	1.822270000	1.162031000	-0.134971000
6	3.155045000	0.871500000	-0.490015000
1	2.601984000	-2.239003000	-1.795083000
1	1.543332000	2.081360000	0.354478000
1	4.410733000	-0.677364000	-1.372896000
1	-2.769874000	1.261886000	0.633212000
1	-2.416216000	-3.270312000	-1.304090000
1	-4.603716000	-2.464843000	-0.620517000
8	-0.450216000	0.634068000	-0.007977000
16	0.226600000	-3.525441000	-1.850081000
7	-5.139015000	-0.015751000	0.384471000
7	4.164925000	1.762471000	-0.255992000

Xanthione

6	-5.282550000	1.342698000	0.930703000
1	-4.619775000	2.021256000	0.386319000
1	-6.304403000	1.666143000	0.720321000
6	-5.008720000	1.432150000	2.433250000
1	-4.002930000	1.076047000	2.672929000
1	-5.725646000	0.832881000	3.000711000
1	-5.090530000	2.470259000	2.768883000
6	-6.344669000	-0.850371000	0.293767000
1	-7.200312000	-0.172817000	0.269167000
1	-6.345816000	-1.380358000	-0.663456000
6	-6.499549000	-1.838200000	1.451322000
1	-6.588101000	-1.310349000	2.404227000
1	-5.640736000	-2.511940000	1.516021000
1	-7.398962000	-2.445133000	1.311385000
6	3.907593000	3.015021000	0.467134000
1	4.739988000	3.687225000	0.251182000
1	3.012730000	3.491434000	0.053797000
6	3.767326000	2.834744000	1.980079000
1	4.694737000	2.450801000	2.412376000
1	2.963884000	2.135291000	2.226281000
1	3.540110000	3.794010000	2.454293000
6	5.555711000	1.465126000	-0.630313000
1	5.564204000	0.971423000	-1.605475000
1	6.058455000	2.424706000	-0.770943000
6	6.310822000	0.627281000	0.402459000
1	5.818081000	-0.331284000	0.588511000
1	6.386047000	1.161855000	1.353795000
1	7.327102000	0.427519000	0.048085000
6	3.693897000	-2.564248000	1.554916000
8	4.875970000	-2.497254000	1.176815000
6	2.857928000	-3.781582000	1.283478000
6	3.039857000	-1.433566000	2.295259000

Xanthione

1	3.483106000	-4.592378000	0.908411000
1	3.694850000	-0.562757000	2.318403000
1	2.095662000	-3.548740000	0.529898000
1	2.331959000	-4.104382000	2.186608000
1	2.814488000	-1.743457000	3.321697000
1	2.090607000	-1.165098000	1.823262000
6	-1.839076000	3.407367000	-1.049834000
8	-2.576904000	3.496919000	-0.052686000
6	-0.476196000	4.036736000	-1.068573000
6	-2.268135000	2.667771000	-2.283247000
1	-0.227675000	4.429951000	-0.082471000
1	-3.299536000	2.329739000	-2.187855000
1	-0.451420000	4.853424000	-1.798178000
1	0.276917000	3.305622000	-1.375944000
1	-1.619206000	1.799007000	-2.436004000
1	-2.167368000	3.305114000	-3.167180000

2. L_a

6	-2.721565000	-0.292492000	-0.195340000
6	-3.901850000	0.469993000	-0.044819000
6	-3.768332000	1.768023000	0.504499000
6	-2.535160000	2.248434000	0.913184000
6	-1.355546000	1.486216000	0.800826000
6	-1.511378000	0.206306000	0.226313000
6	-0.055461000	1.907654000	1.215869000
6	1.063852000	1.040545000	1.027541000
6	0.862293000	-0.225742000	0.437081000
6	2.396740000	1.349454000	1.364876000
6	3.443014000	0.476308000	1.124022000
6	1.887290000	-1.111409000	0.182447000
6	3.221199000	-0.785165000	0.523251000

Xanthione

1	2.618375000	2.312282000	1.812167000
1	1.630975000	-2.040521000	-0.302178000
1	4.442752000	0.793422000	1.377931000
1	-2.732130000	-1.287018000	-0.611472000
1	-2.480996000	3.246554000	1.334470000
1	-4.631810000	2.406471000	0.619647000
8	-0.414665000	-0.645457000	0.068419000
16	0.167164000	3.500891000	1.988910000
7	-5.128837000	-0.050701000	-0.403972000
7	4.255576000	-1.664962000	0.277240000
6	-5.237420000	-1.407336000	-0.952283000
1	-4.575820000	-2.076956000	-0.394531000
1	-6.258174000	-1.750052000	-0.763838000
6	-4.931862000	-1.499409000	-2.449567000
1	-3.927236000	-1.127109000	-2.669401000
1	-5.646322000	-0.912163000	-3.032987000
1	-4.990107000	-2.539486000	-2.785149000
6	-6.338097000	0.774090000	-0.354397000
1	-7.192485000	0.093383000	-0.358404000
1	-6.374924000	1.305840000	0.602590000
6	-6.464483000	1.765783000	-1.513712000
1	-6.526916000	1.239308000	-2.469704000
1	-5.603197000	2.438343000	-1.553563000
1	-7.367009000	2.373707000	-1.397699000
6	4.013711000	-2.931121000	-0.419020000
1	4.857700000	-3.587960000	-0.196312000
1	3.129408000	-3.414866000	0.009458000
6	3.857405000	-2.791378000	-1.935763000
1	4.775296000	-2.404692000	-2.386167000
1	3.042686000	-2.107929000	-2.190160000
1	3.638987000	-3.764513000	-2.386050000
6	5.642913000	-1.298881000	0.578739000

Xanthione

1	5.681188000	-0.823755000	1.564140000
1	6.207536000	-2.230237000	0.668061000
6	6.297978000	-0.396438000	-0.470436000
1	5.741543000	0.534449000	-0.611300000
1	6.354928000	-0.906362000	-1.436542000
1	7.317563000	-0.142581000	-0.162625000
6	3.440340000	2.514585000	-1.688623000
8	4.588620000	2.700096000	-1.251554000
6	2.350685000	3.527603000	-1.476716000
6	3.077881000	1.271842000	-2.448376000
1	2.700636000	4.327836000	-0.824398000
1	3.965039000	0.667046000	-2.636331000
1	1.468995000	3.054579000	-1.034492000
1	2.043751000	3.953616000	-2.438199000
1	2.593905000	1.525648000	-3.396381000
1	2.363224000	0.681458000	-1.865613000
6	-1.746116000	-3.412574000	1.077231000
8	-2.483640000	-3.539028000	0.084020000
6	-0.364833000	-4.000521000	1.101519000
6	-2.193429000	-2.669111000	2.301688000
1	-0.105128000	-4.396217000	0.119224000
1	-3.238377000	-2.374367000	2.209964000
1	-0.315431000	-4.808809000	1.839272000
1	0.365503000	-3.243268000	1.399693000
1	-1.578955000	-1.771714000	2.427720000
1	-2.057882000	-3.284431000	3.196437000

3. L_b

6	-2.729631000	0.282126000	0.213501000
6	-3.905671000	-0.512949000	0.060270000

Xanthione

6	-3.745579000	-1.830890000	-0.467720000
6	-2.510609000	-2.293699000	-0.849702000
6	-1.327464000	-1.497738000	-0.747154000
6	-1.515340000	-0.198301000	-0.183849000
6	-0.029481000	-1.932546000	-1.169014000
6	1.053221000	-1.009184000	-0.985728000
6	0.839612000	0.260984000	-0.399888000
6	2.397698000	-1.282485000	-1.336094000
6	3.423890000	-0.392914000	-1.103645000
6	1.842165000	1.168962000	-0.149293000
6	3.182365000	0.868717000	-0.495025000
1	2.608570000	-2.242018000	-1.789354000
1	1.567163000	2.094876000	0.331261000
1	4.428675000	-0.686746000	-1.366803000
1	-2.767327000	1.282567000	0.612806000
1	-2.398558000	-3.289202000	-1.255457000
1	-4.600791000	-2.480321000	-0.582727000
8	-0.443482000	0.671615000	-0.026644000
16	0.194105000	-3.560623000	-1.866509000
7	-5.125481000	-0.007002000	0.393159000
7	4.197112000	1.763566000	-0.252518000
6	-5.271408000	1.370192000	0.899165000
1	-4.601621000	2.030897000	0.343318000
1	-6.292341000	1.683348000	0.674623000
6	-5.007319000	1.490389000	2.403150000
1	-4.000384000	1.148016000	2.655793000
1	-5.725098000	0.903220000	2.980854000
1	-5.098796000	2.537004000	2.706689000
6	-6.338263000	-0.837012000	0.322592000
1	-7.190099000	-0.156711000	0.299601000
1	-6.342201000	-1.380398000	-0.626291000
6	-6.475602000	-1.807646000	1.499224000

Xanthione

1	-6.559256000	-1.267126000	2.444681000
1	-5.615844000	-2.479164000	1.563403000
1	-7.376023000	-2.414469000	1.370293000
6	3.940462000	3.014241000	0.469676000
1	4.777272000	3.683608000	0.261085000
1	3.049590000	3.495353000	0.052696000
6	3.788050000	2.834354000	1.982844000
1	4.710283000	2.446070000	2.422237000
1	2.979322000	2.138365000	2.221237000
1	3.561623000	3.795105000	2.454678000
6	5.587549000	1.450308000	-0.604428000
1	5.604835000	0.963266000	-1.583395000
1	6.108679000	2.402808000	-0.726089000
6	6.313680000	0.589649000	0.432800000
1	5.800095000	-0.360392000	0.605042000
1	6.386940000	1.115954000	1.388825000
1	7.330262000	0.373379000	0.088956000
6	3.636494000	-2.568930000	1.563294000
8	4.826453000	-2.531407000	1.205471000
6	2.772808000	-3.761379000	1.270386000
6	2.999833000	-1.423630000	2.296023000
1	3.383565000	-4.588742000	0.907533000
1	3.696584000	-0.589698000	2.379072000
1	2.035618000	-3.506639000	0.498293000
1	2.217337000	-4.071304000	2.160273000
1	2.693805000	-1.743576000	3.297883000
1	2.098733000	-1.093133000	1.771411000
6	-1.846722000	3.380598000	-1.078824000
8	-2.628712000	3.436880000	-0.112343000
6	-0.508372000	4.057737000	-1.037034000
6	-2.197094000	2.632034000	-2.330823000
1	-0.313146000	4.449524000	-0.038597000

Xanthione

1	-3.221049000	2.262187000	-2.283068000
1	-0.483281000	4.881704000	-1.758429000
1	0.280646000	3.355340000	-1.320313000
1	-1.514146000	1.784898000	-2.453447000
1	-2.076411000	3.273558000	-3.209016000

C. S=4

1. S_0

6	-2.296708000	0.654734000	-0.402947000
6	-3.591261000	0.153250000	-0.633256000
6	-3.695134000	-1.105445000	-1.302256000
6	-2.574624000	-1.786366000	-1.708202000
6	-1.265598000	-1.294477000	-1.492126000
6	-1.192881000	-0.049681000	-0.837373000
6	-0.065346000	-1.988514000	-1.885227000
6	1.177380000	-1.324848000	-1.606439000
6	1.202687000	-0.076749000	-0.948164000
6	2.449431000	-1.865215000	-1.916024000
6	3.617390000	-1.230752000	-1.584334000
6	2.355271000	0.589950000	-0.594063000
6	3.610520000	0.026959000	-0.902252000
1	2.478820000	-2.822196000	-2.419893000
1	2.248993000	1.519461000	-0.051700000
1	4.552881000	-1.709726000	-1.830065000
1	-2.122014000	1.599991000	0.083064000
1	-2.675370000	-2.738465000	-2.212095000
1	-4.664075000	-1.540189000	-1.496272000
8	0.024366000	0.547493000	-0.577222000
16	-0.124281000	-3.566300000	-2.621077000
7	-4.697119000	0.851747000	-0.234478000

Xanthione

7	4.771075000	0.661261000	-0.564423000
6	-4.555283000	2.131531000	0.476947000
1	-3.782252000	2.731397000	-0.012477000
1	-5.495015000	2.673623000	0.353220000
6	-4.237908000	1.973721000	1.965872000
1	-3.326736000	1.387819000	2.117059000
1	-5.054166000	1.471090000	2.490954000
1	-4.091056000	2.955858000	2.425407000
6	-6.046084000	0.287766000	-0.376847000
1	-6.749199000	1.120031000	-0.308477000
1	-6.159131000	-0.127192000	-1.382802000
6	-6.386672000	-0.767387000	0.677388000
1	-6.358178000	-0.338270000	1.682146000
1	-5.681250000	-1.602466000	0.645153000
1	-7.391075000	-1.164364000	0.503425000
6	4.742274000	1.902465000	0.224408000
1	5.680442000	2.428094000	0.031230000
1	3.939263000	2.543248000	-0.144227000
6	4.565077000	1.666838000	1.726386000
1	5.465669000	1.230172000	2.166402000
1	3.735689000	0.981008000	1.914935000
1	4.355230000	2.613425000	2.231367000
6	6.077323000	0.030058000	-0.795067000
1	6.093269000	-0.399944000	-1.800229000
1	6.822767000	0.828337000	-0.793367000
6	6.439973000	-1.029848000	0.248478000
1	5.631114000	-1.754263000	0.373425000
1	6.620797000	-0.569465000	1.223097000
1	7.348125000	-1.561335000	-0.051526000
6	2.229172000	-2.157568000	1.876114000
8	3.415319000	-1.798757000	1.970265000
6	1.856343000	-3.493717000	1.300861000

Xanthione

6	1.108134000	-1.275167000	2.347387000
1	2.751835000	-4.089533000	1.122639000
1	1.499640000	-0.321603000	2.701112000
1	1.329104000	-3.356488000	0.350574000
1	1.172023000	-4.020447000	1.972213000
1	0.552271000	-1.775885000	3.146007000
1	0.395304000	-1.094416000	1.537687000
6	-0.905971000	3.471230000	-1.780247000
8	-1.341678000	3.752612000	-0.647997000
6	0.546061000	3.619133000	-2.125690000
6	-1.816768000	2.960121000	-2.859206000
1	1.118782000	3.946902000	-1.259756000
1	-2.851936000	2.954994000	-2.518892000
1	0.666004000	4.334189000	-2.946248000
1	0.945043000	2.660113000	-2.471682000
1	-1.527625000	1.941401000	-3.137903000
1	-1.726913000	3.578280000	-3.757858000
6	-1.859717000	-3.379260000	2.128530000
8	-1.025194000	-3.686580000	2.999181000
6	-2.649694000	-2.107078000	2.224670000
6	-2.078032000	-4.247592000	0.924221000
1	-2.379859000	-1.559803000	3.128057000
1	-1.714457000	-5.257520000	1.117097000
1	-3.722700000	-2.323883000	2.235577000
1	-2.462848000	-1.478760000	1.348500000
1	-1.519574000	-3.839185000	0.072718000
1	-3.131061000	-4.275066000	0.634954000
6	0.809067000	3.669704000	1.614916000
8	1.842705000	3.425698000	0.964207000
6	0.354087000	5.077792000	1.845432000
6	-0.028630000	2.567259000	2.191153000
1	1.146347000	5.783513000	1.594083000

Xanthione

1	0.256074000	2.405798000	3.237918000
1	0.030769000	5.226981000	2.879172000
1	-0.507703000	5.254774000	1.192994000
1	0.128594000	1.644600000	1.633891000
1	-1.087263000	2.834284000	2.173206000

2. *L_a*

6	2.350761000	0.511665000	0.319489000
6	3.656661000	0.034760000	0.566790000
6	3.775107000	-1.192062000	1.263063000
6	2.653869000	-1.875566000	1.705651000
6	1.345038000	-1.400029000	1.484680000
6	1.255125000	-0.184593000	0.773892000
6	0.141516000	-2.048304000	1.902893000
6	-1.123772000	-1.443401000	1.625954000
6	-1.163807000	-0.220378000	0.921090000
6	-2.379966000	-1.973738000	1.983044000
6	-3.567601000	-1.335468000	1.667155000
6	-2.325170000	0.438337000	0.592548000
6	-3.576076000	-0.097582000	0.977236000
1	-2.423567000	-2.916471000	2.517963000
1	-2.235725000	1.358094000	0.030027000
1	-4.494907000	-1.801476000	1.965967000
1	2.168731000	1.437331000	-0.200780000
1	2.793733000	-2.810042000	2.238174000
1	4.747620000	-1.615836000	1.465531000
8	0.016979000	0.384652000	0.483324000
16	0.219388000	-3.597287000	2.786318000
7	4.758004000	0.753699000	0.148753000
7	-4.748091000	0.573964000	0.702824000

Xanthione

6	4.592220000	2.025890000	-0.563587000
1	3.812078000	2.614491000	-0.070344000
1	5.523171000	2.585571000	-0.443990000
6	4.273736000	1.870086000	-2.053485000
1	3.369034000	1.274373000	-2.205656000
1	5.095043000	1.376828000	-2.580207000
1	4.115921000	2.851421000	-2.511951000
6	6.105872000	0.191043000	0.260941000
1	6.810356000	1.019119000	0.156113000
1	6.249528000	-0.207178000	1.271149000
6	6.417267000	-0.887337000	-0.780337000
1	6.368520000	-0.476348000	-1.792094000
1	5.704948000	-1.714745000	-0.716439000
1	7.422161000	-1.290455000	-0.622220000
6	-4.735062000	1.884954000	0.043627000
1	-5.608944000	2.436097000	0.404438000
1	-3.856254000	2.442111000	0.372789000
6	-4.756426000	1.818814000	-1.484678000
1	-5.713759000	1.433108000	-1.848092000
1	-3.969591000	1.160960000	-1.860626000
1	-4.606719000	2.817986000	-1.904385000
6	-6.047468000	-0.062871000	0.930002000
1	-6.074948000	-0.486748000	1.940011000
1	-6.799809000	0.729105000	0.910783000
6	-6.400276000	-1.135899000	-0.103449000
1	-5.627266000	-1.907585000	-0.150920000
1	-6.491929000	-0.697978000	-1.100294000
1	-7.350228000	-1.614522000	0.153501000
6	-2.398023000	-1.620032000	-2.329711000
8	-3.552699000	-1.247470000	-2.601317000
6	-2.139001000	-2.931432000	-1.646835000
6	-1.201241000	-0.784802000	-2.683239000

Xanthione

1	-3.069097000	-3.489001000	-1.533949000
1	-1.516855000	0.153389000	-3.139988000
1	-1.708846000	-2.752499000	-0.655596000
1	-1.409993000	-3.515163000	-2.216954000
1	-0.553032000	-1.338619000	-3.369879000
1	-0.608699000	-0.569268000	-1.789072000
6	1.069762000	3.224627000	1.929098000
8	1.462387000	3.640521000	0.823244000
6	-0.377112000	3.278865000	2.322184000
6	2.028981000	2.643471000	2.927843000
1	-0.979779000	3.708343000	1.523424000
1	3.044774000	2.651971000	2.533569000
1	-0.498574000	3.864392000	3.239189000
1	-0.741860000	2.268112000	2.531472000
1	1.745373000	1.613459000	3.165927000
1	1.992361000	3.214062000	3.861689000
6	1.602743000	-3.213439000	-2.288233000
8	0.798185000	-3.368901000	-3.225377000
6	2.638321000	-2.129676000	-2.322399000
6	1.532292000	-4.073726000	-1.060248000
1	2.572272000	-1.570872000	-3.256218000
1	1.001303000	-5.001083000	-1.277509000
1	3.641839000	-2.554524000	-2.219080000
1	2.494653000	-1.447081000	-1.478667000
1	0.983037000	-3.535111000	-0.279332000
1	2.525716000	-4.291523000	-0.662435000
6	-0.784513000	3.754977000	-1.366326000
8	-1.822291000	3.378459000	-0.789872000
6	-0.404456000	5.203253000	-1.409522000
6	0.133872000	2.776845000	-2.036393000
1	-1.237287000	5.829415000	-1.088257000
1	-0.126724000	2.704041000	-3.099200000

Xanthione

1	-0.077203000	5.497144000	-2.410723000
1	0.440026000	5.341610000	-0.725889000
1	0.028728000	1.794305000	-1.577622000
1	1.171537000	3.108646000	-1.967916000

3. L_b

6	-2.294161000	0.637628000	-0.371164000
6	-3.599581000	0.152840000	-0.614444000
6	-3.709802000	-1.099676000	-1.279741000
6	-2.588747000	-1.798673000	-1.676214000
6	-1.271257000	-1.326933000	-1.450724000
6	-1.198958000	-0.077217000	-0.790790000
6	-0.080780000	-2.027820000	-1.837145000
6	1.171188000	-1.373626000	-1.566081000
6	1.208896000	-0.112841000	-0.903378000
6	2.448657000	-1.921966000	-1.894802000
6	3.624708000	-1.290265000	-1.575673000
6	2.360943000	0.542479000	-0.570709000
6	3.626512000	-0.033840000	-0.895067000
1	2.458823000	-2.879031000	-2.397111000
1	2.269877000	1.477012000	-0.031925000
1	4.555877000	-1.771519000	-1.834377000
1	-2.114251000	1.582076000	0.115513000
1	-2.696643000	-2.750061000	-2.179792000
1	-4.681329000	-1.525937000	-1.483326000
8	0.032507000	0.516657000	-0.510873000
16	-0.114212000	-3.624915000	-2.628418000
7	-4.700497000	0.877892000	-0.225337000
7	4.780180000	0.599309000	-0.549787000
6	-4.542895000	2.150074000	0.492309000
1	-3.759843000	2.742700000	0.009184000

Xanthione

1	-5.475201000	2.705537000	0.370424000
6	-4.231300000	1.982094000	1.982641000
1	-3.323611000	1.390849000	2.133137000
1	-5.052287000	1.480865000	2.501643000
1	-4.081070000	2.961500000	2.447111000
6	-6.054955000	0.340247000	-0.389991000
1	-6.745335000	1.184395000	-0.335692000
1	-6.155560000	-0.077016000	-1.396720000
6	-6.434502000	-0.708246000	0.659307000
1	-6.422408000	-0.277164000	1.663555000
1	-5.739806000	-1.552519000	0.643021000
1	-7.440462000	-1.090816000	0.463022000
6	4.762858000	1.861548000	0.209553000
1	5.694234000	2.383461000	-0.018335000
1	3.945128000	2.486638000	-0.148591000
6	4.624356000	1.647192000	1.721083000
1	5.542163000	1.237146000	2.149539000
1	3.810911000	0.951762000	1.938893000
1	4.411019000	2.603887000	2.204346000
6	6.087432000	-0.037229000	-0.768737000
1	6.106331000	-0.469201000	-1.772286000
1	6.836962000	0.755296000	-0.753364000
6	6.416955000	-1.101544000	0.284999000
1	5.586945000	-1.800039000	0.411645000
1	6.613142000	-0.640402000	1.255408000
1	7.309445000	-1.656176000	-0.017976000
6	2.152812000	-2.020314000	1.872351000
8	3.339776000	-1.649342000	1.842523000
6	1.732397000	-3.348776000	1.314746000
6	1.080461000	-1.152857000	2.468127000
1	2.602453000	-3.901074000	0.959920000
1	1.520885000	-0.263084000	2.918525000

Xanthione

1	1.038418000	-3.204285000	0.479449000
1	1.194885000	-3.925382000	2.073182000
1	0.506722000	-1.713162000	3.211610000
1	0.373560000	-0.842121000	1.691724000
6	-0.932016000	3.402303000	-1.828744000
8	-1.324018000	3.741260000	-0.696380000
6	0.515633000	3.471663000	-2.215305000
6	-1.893176000	2.899731000	-2.866921000
1	1.129865000	3.764611000	-1.365374000
1	-2.919598000	2.972632000	-2.508006000
1	0.650068000	4.186574000	-3.034067000
1	0.851677000	2.495964000	-2.580052000
1	-1.671692000	1.853032000	-3.100157000
1	-1.785063000	3.468691000	-3.795483000
6	-1.856945000	-3.341985000	2.175405000
8	-1.052209000	-3.614568000	3.085225000
6	-2.683321000	-2.090215000	2.220335000
6	-2.000084000	-4.229208000	0.974211000
1	-2.500667000	-1.548915000	3.148892000
1	-1.542797000	-5.200207000	1.166195000
1	-3.748311000	-2.327653000	2.136827000
1	-2.435101000	-1.446800000	1.370409000
1	-1.496434000	-3.771268000	0.113627000
1	-3.049655000	-4.356646000	0.697630000
6	0.899542000	3.708012000	1.512196000
8	1.921458000	3.373354000	0.881973000
6	0.517346000	5.148505000	1.653365000
6	0.006214000	2.685810000	2.147952000
1	1.344337000	5.796579000	1.362089000
1	0.279715000	2.572549000	3.203965000
1	0.200558000	5.377321000	2.674447000
1	-0.334652000	5.327089000	0.988431000

Xanthione

1	0.117869000	1.725516000	1.645798000
1	-1.037160000	3.004784000	2.106594000

IV. OPTIMISED GEOMETRIES - ACETONITRILE

A. Implicit

1. S_0

6	2.308166000	-0.785911000	-0.357272000
6	3.581930000	-0.205333000	-0.292981000
6	3.639060000	1.206005000	-0.111417000
6	2.502260000	1.952013000	-0.029996000
6	1.214933000	1.387322000	-0.105171000
6	1.175841000	-0.003195000	-0.271616000
6	-0.000006000	2.152790000	-0.016529000
6	-1.214962000	1.387273000	-0.104980000
6	-1.175838000	-0.003238000	-0.271471000
6	-2.502282000	1.951920000	-0.029631000
6	-3.639078000	1.205868000	-0.110966000
6	-2.308138000	-0.785993000	-0.357013000
6	-3.581912000	-0.205432000	-0.292677000
1	-2.571289000	3.021983000	0.104113000
1	-2.166263000	-1.848048000	-0.470826000
1	-4.590450000	1.706349000	-0.034688000
1	2.166293000	-1.847963000	-0.471134000
1	2.571230000	3.022086000	0.103687000
1	4.590416000	1.706532000	-0.035289000
8	0.000017000	-0.676558000	-0.351535000
16	-0.000035000	3.832094000	0.177573000
7	4.712546000	-0.948405000	-0.411980000
7	-4.712547000	-0.948531000	-0.411587000

Xanthione

6	4.642426000	-2.397019000	-0.561510000
1	3.860192000	-2.640580000	-1.281089000
1	5.578852000	-2.717903000	-1.014166000
6	4.417123000	-3.153372000	0.741844000
1	3.505566000	-2.825372000	1.240858000
1	5.250457000	-3.006972000	1.427964000
1	4.327771000	-4.221617000	0.540444000
6	6.034487000	-0.345964000	-0.274597000
1	6.740501000	-1.017266000	-0.759926000
1	6.065136000	0.583991000	-0.842148000
6	6.466257000	-0.102257000	1.165784000
1	6.538144000	-1.039082000	1.717082000
1	5.762016000	0.544905000	1.688262000
1	7.445434000	0.378025000	1.183826000
6	-4.642429000	-2.397064000	-0.561803000
1	-5.578923000	-2.717709000	-1.014481000
1	-3.860266000	-2.640320000	-1.281576000
6	-4.417045000	-3.154087000	0.741155000
1	-5.250247000	-3.007854000	1.427473000
1	-3.505347000	-2.826490000	1.240173000
1	-4.327907000	-4.222258000	0.539243000
6	-6.034411000	-0.345914000	-0.274561000
1	-6.064958000	0.583843000	-0.842476000
1	-6.740469000	-1.017344000	-0.759659000
6	-6.466313000	-0.101652000	1.165686000
1	-5.762107000	0.545664000	1.688019000
1	-6.538272000	-1.038296000	1.717285000
1	-7.445484000	0.378658000	1.183464000

Xanthione

2. L_a

6	2.322692000	-0.778947000	-0.359784000
6	3.602429000	-0.195119000	-0.306318000
6	3.658148000	1.202320000	-0.140523000
6	2.507766000	1.955924000	-0.057625000
6	1.229078000	1.387321000	-0.119256000
6	1.189463000	-0.006959000	-0.274443000
6	-0.000008000	2.111440000	-0.025039000
6	-1.229072000	1.387269000	-0.119126000
6	-1.189403000	-0.007017000	-0.274378000
6	-2.507767000	1.955816000	-0.057363000
6	-3.658139000	1.202147000	-0.140130000
6	-2.322594000	-0.779050000	-0.359601000
6	-3.602355000	-0.195260000	-0.305983000
1	-2.601264000	3.028428000	0.063531000
1	-2.186102000	-1.843133000	-0.466877000
1	-4.606745000	1.710167000	-0.075817000
1	2.186183000	-1.843041000	-0.466951000
1	2.601216000	3.028551000	0.063184000
1	4.606736000	1.710403000	-0.076487000
8	0.000049000	-0.684093000	-0.339203000
16	-0.000026000	3.812017000	0.249136000
7	4.737594000	-0.955239000	-0.432743000
7	-4.737449000	-0.955481000	-0.432349000
6	4.658958000	-2.403765000	-0.535452000
1	3.876684000	-2.669491000	-1.248251000
1	5.594135000	-2.745245000	-0.977152000
6	4.427953000	-3.125733000	0.787751000
1	3.518178000	-2.777965000	1.276851000
1	5.261314000	-2.965715000	1.471085000
1	4.331447000	-4.198967000	0.616534000

Xanthione

6	6.055318000	-0.353761000	-0.301042000
1	6.764528000	-1.028963000	-0.777949000
1	6.085805000	0.570794000	-0.879543000
6	6.493721000	-0.086582000	1.134757000
1	6.579998000	-1.015814000	1.697093000
1	5.783631000	0.557646000	1.652938000
1	7.467158000	0.406212000	1.143223000
6	-4.658688000	-2.404054000	-0.534300000
1	-5.594155000	-2.745837000	-0.975127000
1	-3.876867000	-2.670191000	-1.247473000
6	-4.426877000	-3.125242000	0.789197000
1	-5.259783000	-2.964657000	1.472959000
1	-3.516743000	-2.777323000	1.277500000
1	-4.330631000	-4.198596000	0.618582000
6	-6.055268000	-0.353891000	-0.302177000
1	-6.084883000	0.570750000	-0.880572000
1	-6.763982000	-1.028930000	-0.780072000
6	-6.495350000	-0.086922000	1.133138000
1	-5.785758000	0.557092000	1.652276000
1	-6.582391000	-1.016268000	1.695178000
1	-7.468737000	0.405986000	1.140611000

3. L_b

6	2.323507000	-0.777655000	-0.371854000
6	3.601358000	-0.191576000	-0.297556000
6	3.647421000	1.207833000	-0.113256000
6	2.497114000	1.954565000	-0.032746000
6	1.215070000	1.387883000	-0.115176000
6	1.193074000	-0.003433000	-0.288955000
6	-0.008733000	2.130795000	-0.026178000

Xanthione

6	-1.216124000	1.385399000	-0.109972000
6	-1.177667000	-0.033715000	-0.290829000
6	-2.517582000	1.957620000	-0.023077000
6	-3.653415000	1.206132000	-0.110398000
6	-2.299353000	-0.795371000	-0.379777000
6	-3.593132000	-0.202513000	-0.297300000
1	-2.577305000	3.025659000	0.118789000
1	-2.170336000	-1.857213000	-0.509745000
1	-4.606532000	1.703495000	-0.032897000
1	2.188158000	-1.840173000	-0.494071000
1	2.564124000	3.024277000	0.106612000
1	4.594340000	1.716847000	-0.030386000
8	0.005818000	-0.693225000	-0.379967000
16	-0.067596000	3.857085000	0.186435000
7	4.738427000	-0.944171000	-0.416545000
7	-4.708745000	-0.955836000	-0.401340000
6	4.667121000	-2.391955000	-0.542256000
1	3.894063000	-2.649157000	-1.267848000
1	5.609135000	-2.722941000	-0.976728000
6	4.422646000	-3.131625000	0.768685000
1	3.504894000	-2.794548000	1.250103000
1	5.246493000	-2.976949000	1.464502000
1	4.333730000	-4.202674000	0.581114000
6	6.054135000	-0.339182000	-0.271635000
1	6.767626000	-1.006031000	-0.753423000
1	6.082744000	0.592847000	-0.837393000
6	6.481802000	-0.090344000	1.170590000
1	6.565654000	-1.026607000	1.721360000
1	5.767340000	0.546597000	1.691626000
1	7.454488000	0.403340000	1.190954000
6	-4.638500000	-2.406592000	-0.565883000
1	-5.578919000	-2.719691000	-1.012929000

Xanthione

1	-3.859154000	-2.642182000	-1.288688000
6	-4.407019000	-3.163149000	0.737283000
1	-5.232549000	-3.013410000	1.431042000
1	-3.486705000	-2.843720000	1.224822000
1	-4.329797000	-4.229879000	0.525756000
6	-6.038000000	-0.364336000	-0.260071000
1	-6.072089000	0.564215000	-0.828280000
1	-6.739026000	-1.045347000	-0.736429000
6	-6.452762000	-0.122129000	1.186924000
1	-5.747662000	0.531138000	1.699328000
1	-6.517225000	-1.057778000	1.739826000
1	-7.434054000	0.352460000	1.205584000

B. S=2

1. S_0

6	-2.535212000	0.551832000	-0.212169000
6	-3.812836000	-0.042783000	-0.215042000
6	-3.871968000	-1.459352000	-0.396631000
6	-2.732398000	-2.200339000	-0.574641000
6	-1.442120000	-1.620647000	-0.584827000
6	-1.408233000	-0.224549000	-0.399700000
6	-0.225719000	-2.372756000	-0.756638000
6	0.999803000	-1.622910000	-0.711204000
6	0.987018000	-0.224808000	-0.529017000
6	2.285359000	-2.205127000	-0.819945000
6	3.436893000	-1.467155000	-0.739457000
6	2.124923000	0.549216000	-0.447163000
6	3.395414000	-0.051284000	-0.547426000
1	2.339128000	-3.276551000	-0.959822000

Xanthione

1	1.999078000	1.607605000	-0.286524000
1	4.385010000	-1.978377000	-0.808462000
1	-2.391460000	1.608216000	-0.049802000
1	-2.801239000	-3.271433000	-0.710192000
1	-4.824060000	-1.967784000	-0.390420000
8	-0.207859000	0.461638000	-0.389360000
16	-0.240714000	-4.098125000	-0.992837000
7	-4.947839000	0.704144000	-0.060643000
7	4.537551000	0.693544000	-0.464973000
6	-4.869638000	2.155707000	0.152185000
1	-4.137533000	2.577813000	-0.543207000
1	-5.837185000	2.575247000	-0.129955000
6	-4.532500000	2.552137000	1.590783000
1	-3.585457000	2.111826000	1.914194000
1	-5.313378000	2.219260000	2.279212000
1	-4.447778000	3.639825000	1.671137000
6	-6.275135000	0.074569000	-0.003955000
1	-7.005928000	0.848507000	-0.246294000
1	-6.351507000	-0.676443000	-0.795317000
6	-6.605970000	-0.543352000	1.355367000
1	-6.620637000	0.220240000	2.137240000
1	-5.870620000	-1.302241000	1.636422000
1	-7.590898000	-1.018362000	1.323342000
6	4.472209000	2.141568000	-0.218171000
1	5.417638000	2.568329000	-0.559589000
1	3.688685000	2.577996000	-0.843224000
6	4.234775000	2.506279000	1.248832000
1	5.072145000	2.186532000	1.874123000
1	3.326856000	2.032876000	1.631981000
1	4.123987000	3.589657000	1.352021000
6	5.862179000	0.057095000	-0.456615000
1	5.898734000	-0.705560000	-1.239226000

Xanthione

1	6.587057000	0.821981000	-0.742490000
6	6.241501000	-0.544769000	0.898168000
1	5.467602000	-1.228585000	1.256540000
1	6.366754000	0.238456000	1.650346000
1	7.184201000	-1.094176000	0.817886000
6	1.884968000	-0.815361000	2.823074000
7	3.025340000	-1.053424000	2.864462000
6	0.461743000	-0.519259000	2.781067000
1	0.278661000	0.394466000	2.211836000
1	0.069834000	-0.384665000	3.791483000
1	-0.083795000	-1.338003000	2.305966000
6	0.373764000	3.616807000	-1.504159000
7	1.315200000	3.897807000	-0.876365000
6	-0.791528000	3.240317000	-2.286191000
1	-1.680634000	3.763862000	-1.927407000
1	-0.960893000	2.165272000	-2.199144000
1	-0.643583000	3.490170000	-3.338902000

2. L_a

6	-2.554941000	-0.556130000	0.238058000
6	-3.838088000	0.038140000	0.245520000
6	-3.897438000	1.441810000	0.418885000
6	-2.747061000	2.192691000	0.591164000
6	-1.463188000	1.612415000	0.598995000
6	-1.427174000	0.213860000	0.416539000
6	-0.234466000	2.325844000	0.755659000
6	1.005979000	1.617621000	0.730311000
6	0.996101000	0.218805000	0.544819000
6	2.281911000	2.204311000	0.846883000
6	3.446658000	1.461498000	0.769241000

Xanthione

6	2.137758000	-0.544315000	0.463981000
6	3.410600000	0.060185000	0.572966000
1	2.359381000	3.276443000	0.992398000
1	2.020016000	-1.602900000	0.294127000
1	4.390780000	1.979657000	0.846958000
1	-2.415801000	-1.614492000	0.080926000
1	-2.843141000	3.265220000	0.721714000
1	-4.848067000	1.953902000	0.416573000
8	-0.211214000	-0.473001000	0.400522000
16	-0.249278000	4.099995000	0.939769000
7	-4.978603000	-0.727332000	0.104384000
7	4.562631000	-0.695762000	0.494980000
6	-4.890648000	-2.174608000	-0.107430000
1	-4.152967000	-2.594224000	0.584814000
1	-5.854246000	-2.602319000	0.178971000
6	-4.556529000	-2.577685000	-1.545972000
1	-3.613801000	-2.131556000	-1.874547000
1	-5.342132000	-2.251838000	-2.232786000
1	-4.464500000	-3.665324000	-1.623549000
6	-6.301711000	-0.100301000	0.040541000
1	-7.035935000	-0.871820000	0.283513000
1	-6.380706000	0.655526000	0.828746000
6	-6.634004000	0.516914000	-1.320124000
1	-6.655865000	-0.249459000	-2.099425000
1	-5.892453000	1.268394000	-1.604920000
1	-7.615165000	1.000390000	-1.288458000
6	4.496176000	-2.135816000	0.226364000
1	5.440709000	-2.570277000	0.563150000
1	3.712326000	-2.583131000	0.844286000
6	4.259897000	-2.487963000	-1.244882000
1	5.099626000	-2.164681000	-1.865564000
1	3.354527000	-2.006618000	-1.624459000

Xanthione

1	4.144254000	-3.569809000	-1.359680000
6	5.878563000	-0.051640000	0.468837000
1	5.922715000	0.706805000	1.256764000
1	6.615788000	-0.812065000	0.737285000
6	6.238543000	0.567934000	-0.884807000
1	5.451747000	1.244519000	-1.228823000
1	6.365968000	-0.207289000	-1.645124000
1	7.174731000	1.129831000	-0.810603000
6	1.831722000	0.934395000	-2.795133000
7	2.983898000	1.084107000	-2.890929000
6	0.393246000	0.748952000	-2.689091000
1	0.166833000	-0.185916000	-2.172297000
1	-0.062346000	0.720223000	-3.681226000
1	-0.054900000	1.569170000	-2.123404000
6	0.466428000	-3.714501000	1.314121000
7	1.362590000	-4.043898000	0.644559000
6	-0.639152000	-3.272282000	2.146741000
1	-1.569368000	-3.755325000	1.838727000
1	-0.756467000	-2.191331000	2.046766000
1	-0.452082000	-3.516213000	3.194585000

3. L_b

6	-2.542626000	0.569017000	-0.205386000
6	-3.828102000	-0.024175000	-0.231952000
6	-3.884656000	-1.430371000	-0.438741000
6	-2.738122000	-2.173605000	-0.615880000
6	-1.443878000	-1.599682000	-0.602339000
6	-1.418711000	-0.200898000	-0.393556000
6	-0.229674000	-2.347268000	-0.765044000
6	0.997387000	-1.608124000	-0.706198000

Xanthione

6	0.991507000	-0.194619000	-0.499490000
6	2.292530000	-2.200744000	-0.823435000
6	3.448978000	-1.467423000	-0.736417000
6	2.126506000	0.558405000	-0.407823000
6	3.411125000	-0.055278000	-0.524051000
1	2.330378000	-3.269753000	-0.977809000
1	2.015353000	1.617421000	-0.239276000
1	4.394991000	-1.980762000	-0.821729000
1	-2.402127000	1.623282000	-0.024337000
1	-2.806227000	-3.242463000	-0.768517000
1	-4.836588000	-1.940243000	-0.455255000
8	-0.206548000	0.497462000	-0.354162000
16	-0.206586000	-4.114660000	-1.018913000
7	-4.965023000	0.732459000	-0.075756000
7	4.543711000	0.693044000	-0.429492000
6	-4.882127000	2.177311000	0.165848000
1	-4.140922000	2.610547000	-0.513066000
1	-5.845331000	2.607418000	-0.115991000
6	-4.556330000	2.545470000	1.615845000
1	-3.613238000	2.095787000	1.937794000
1	-5.344076000	2.201708000	2.290923000
1	-4.469425000	3.631342000	1.716905000
6	-6.291871000	0.105987000	-0.039163000
1	-7.021477000	0.885365000	-0.267922000
1	-6.363690000	-0.629997000	-0.845582000
6	-6.631513000	-0.542320000	1.305314000
1	-6.656100000	0.204537000	2.102916000
1	-5.894660000	-1.303634000	1.574968000
1	-7.614181000	-1.020451000	1.254114000
6	4.484635000	2.140983000	-0.161593000
1	5.425128000	2.567908000	-0.512755000
1	3.687241000	2.587951000	-0.758571000

Xanthione

6	4.277948000	2.470008000	1.320439000
1	5.132871000	2.149776000	1.920360000
1	3.384602000	1.978437000	1.713275000
1	4.160208000	3.550576000	1.439481000
6	5.874450000	0.066486000	-0.471771000
1	5.889786000	-0.677024000	-1.272288000
1	6.586360000	0.844151000	-0.750996000
6	6.282575000	-0.566763000	0.862395000
1	5.525870000	-1.272456000	1.213137000
1	6.415362000	0.196030000	1.632893000
1	7.229524000	-1.099915000	0.741367000
6	1.796756000	-0.852681000	2.795918000
7	2.959515000	-0.782570000	2.845832000
6	0.346328000	-0.934333000	2.733540000
1	-0.065315000	-0.027759000	2.284247000
1	-0.074397000	-1.048082000	3.734813000
1	0.038153000	-1.790174000	2.128116000
6	0.458971000	3.636789000	-1.500467000
7	1.484955000	3.834299000	-0.983423000
6	-0.814527000	3.361641000	-2.142838000
1	-1.616281000	3.927094000	-1.662364000
1	-1.042079000	2.296651000	-2.063331000
1	-0.779411000	3.638101000	-3.198693000

C. S=4

1. S_0

6	-2.019233000	-0.170080000	0.040859000
6	-3.292240000	0.431301000	-0.028664000
6	-3.344371000	1.859362000	0.030072000

Xanthione

6	-2.198403000	2.605380000	0.149098000
6	-0.913118000	2.018791000	0.215802000
6	-0.887012000	0.610283000	0.162680000
6	0.305784000	2.775674000	0.330790000
6	1.525310000	2.016820000	0.358725000
6	1.504908000	0.608335000	0.290338000
6	2.811131000	2.600790000	0.443663000
6	3.960014000	1.853976000	0.450554000
6	2.641512000	-0.173845000	0.290191000
6	3.913882000	0.427402000	0.369622000
1	2.868200000	3.679888000	0.495779000
1	2.515685000	-1.242606000	0.227924000
1	4.909671000	2.364630000	0.500322000
1	-1.889306000	-1.238976000	-0.002006000
1	-2.259039000	3.684707000	0.191686000
1	-4.292803000	2.371645000	-0.026118000
8	0.306665000	-0.080956000	0.231736000
16	0.298377000	4.514757000	0.426721000
7	-4.425993000	-0.320470000	-0.144751000
7	5.056155000	-0.323675000	0.373652000
6	-4.348224000	-1.787782000	-0.214455000
1	-3.648505000	-2.145217000	0.544475000
1	-5.330766000	-2.173481000	0.064700000
6	-3.956502000	-2.319703000	-1.594059000
1	-2.993907000	-1.914523000	-1.919215000
1	-4.706946000	-2.057438000	-2.344473000
1	-3.868071000	-3.409081000	-1.561958000
6	-5.743328000	0.307840000	-0.315993000
1	-6.490774000	-0.439342000	-0.043416000
1	-5.851216000	1.124733000	0.403366000
6	-6.003098000	0.807105000	-1.738301000
1	-5.979020000	-0.021094000	-2.451215000

Xanthione

1	-5.252888000	1.541236000	-2.045416000
1	-6.987064000	1.281333000	-1.798190000
6	5.004995000	-1.771985000	0.122457000
1	5.942560000	-2.194235000	0.489328000
1	4.206709000	-2.220810000	0.719540000
6	4.814327000	-2.126344000	-1.353331000
1	5.660101000	-1.779611000	-1.952268000
1	3.903722000	-1.671299000	-1.752789000
1	4.728294000	-3.209857000	-1.470308000
6	6.381506000	0.300798000	0.488497000
1	6.335990000	1.097177000	1.236135000
1	7.057793000	-0.456842000	0.890375000
6	6.932696000	0.838560000	-0.833259000
1	6.254397000	1.572770000	-1.276631000
1	7.075956000	0.030530000	-1.555057000
1	7.899809000	1.322689000	-0.668227000
6	1.388080000	-3.466351000	-0.864059000
7	2.111483000	-3.759010000	0.003183000
6	0.480445000	-3.070134000	-1.925941000
1	-0.550244000	-3.259630000	-1.616200000
1	0.685449000	-3.636343000	-2.837170000
1	0.592497000	-2.003771000	-2.142787000
6	-0.849708000	-3.280555000	1.538519000
7	-1.590707000	-3.745006000	0.766816000
6	0.085089000	-2.672600000	2.468955000
1	1.008315000	-3.253837000	2.506138000
1	-0.352332000	-2.615209000	3.466961000
1	0.329792000	-1.663895000	2.126768000
6	-0.653262000	0.654472000	-3.150601000
7	0.393828000	0.172881000	-2.975383000
6	-1.964019000	1.245103000	-3.362166000
1	-2.738227000	0.475511000	-3.318425000

Xanthione

1	-2.011597000	1.732476000	-4.338140000
1	-2.173872000	1.986695000	-2.588579000
6	-2.662951000	-0.439551000	3.381409000
7	-3.120901000	-1.507984000	3.289856000
6	-2.081982000	0.888289000	3.487402000
1	-1.015565000	0.855897000	3.253103000
1	-2.203975000	1.281238000	4.498840000
1	-2.566602000	1.570223000	2.785266000

2. *L_a*

6	-1.998286000	-0.097087000	0.054209000
6	-3.274629000	0.503372000	-0.055439000
6	-3.322945000	1.918162000	-0.080690000
6	-2.164259000	2.676515000	-0.001114000
6	-0.887103000	2.090380000	0.108194000
6	-0.864606000	0.678108000	0.135446000
6	0.345779000	2.805360000	0.187713000
6	1.579387000	2.087461000	0.248757000
6	1.555012000	0.676069000	0.258444000
6	2.859813000	2.672935000	0.285974000
6	4.018155000	1.915250000	0.318637000
6	2.689310000	-0.102429000	0.286400000
6	3.968734000	0.500406000	0.319006000
1	2.947745000	3.754120000	0.279322000
1	2.562002000	-1.174156000	0.283961000
1	4.967238000	2.430198000	0.329054000
1	-1.872749000	-1.168011000	0.068400000
1	-2.250081000	3.757370000	-0.027843000
1	-4.267285000	2.433043000	-0.175849000
8	0.339505000	-0.013507000	0.255338000

Xanthione

16	0.345901000	4.589131000	0.181713000
7	-4.413694000	-0.269028000	-0.133380000
7	5.114580000	-0.268481000	0.360944000
6	-4.326148000	-1.733810000	-0.125343000
1	-3.636313000	-2.049573000	0.661558000
1	-5.310700000	-2.111964000	0.159664000
6	-3.912047000	-2.343921000	-1.466853000
1	-2.941000000	-1.963094000	-1.796525000
1	-4.647669000	-2.118040000	-2.243784000
1	-3.831110000	-3.430992000	-1.375219000
6	-5.725631000	0.347196000	-0.345467000
1	-6.477866000	-0.381865000	-0.036514000
1	-5.836433000	1.204113000	0.327257000
6	-5.984177000	0.772526000	-1.793016000
1	-5.960374000	-0.092953000	-2.460770000
1	-5.230901000	1.487562000	-2.135989000
1	-6.966668000	1.245982000	-1.881639000
6	5.048961000	-1.724040000	0.186817000
1	5.989902000	-2.134045000	0.561330000
1	4.257166000	-2.137022000	0.818039000
6	4.831406000	-2.165563000	-1.262474000
1	5.663604000	-1.851183000	-1.897762000
1	3.910606000	-1.737531000	-1.668534000
1	4.747430000	-3.254655000	-1.315951000
6	6.438146000	0.357856000	0.406588000
1	6.411537000	1.194582000	1.111612000
1	7.128363000	-0.376145000	0.829973000
6	6.959747000	0.828866000	-0.953737000
1	6.264587000	1.531142000	-1.422057000
1	7.095345000	-0.016650000	-1.633172000
1	7.925546000	1.330125000	-0.837064000
6	1.389393000	-3.683599000	-0.580439000

Xanthione

7	2.148873000	-3.660219000	0.304734000
6	0.428535000	-3.687229000	-1.669046000
1	-0.567257000	-3.904347000	-1.274418000
1	0.691238000	-4.445304000	-2.410284000
1	0.404228000	-2.707863000	-2.157151000
6	-0.862149000	-3.122875000	1.773109000
7	-1.599723000	-3.698920000	1.076817000
6	0.066999000	-2.381263000	2.607535000
1	1.005643000	-2.930446000	2.702039000
1	-0.360407000	-2.212418000	3.597172000
1	0.281337000	-1.418384000	2.136242000
6	-0.923920000	0.048508000	-3.216460000
7	-0.037612000	-0.698411000	-3.097787000
6	-2.032473000	0.976307000	-3.361328000
1	-2.982563000	0.452897000	-3.232526000
1	-2.018596000	1.437767000	-4.350911000
1	-1.970195000	1.760758000	-2.604092000
6	-2.713162000	-0.181241000	3.409578000
7	-3.161259000	-1.257831000	3.410599000
6	-2.145648000	1.156502000	3.403712000
1	-1.066365000	1.110415000	3.242132000
1	-2.334619000	1.657344000	4.355572000
1	-2.586157000	1.749081000	2.598856000

3. L_b

6	-2.004673000	-0.081888000	0.055912000
6	-3.271648000	0.523321000	-0.124568000
6	-3.305328000	1.943272000	-0.184919000
6	-2.149779000	2.691894000	-0.073410000
6	-0.874172000	2.105287000	0.104236000

Xanthione

6	-0.874930000	0.692507000	0.166559000
6	0.349886000	2.846013000	0.203269000
6	1.562600000	2.088189000	0.279063000
6	1.528613000	0.658342000	0.317067000
6	2.868144000	2.668316000	0.303912000
6	4.009581000	1.906580000	0.335883000
6	2.647765000	-0.122615000	0.341286000
6	3.944226000	0.479084000	0.340405000
1	2.928101000	3.747220000	0.286184000
1	2.516398000	-1.193493000	0.372059000
1	4.966260000	2.407434000	0.336668000
1	-1.885618000	-1.152556000	0.103573000
1	-2.200112000	3.771329000	-0.126092000
1	-4.242025000	2.460951000	-0.330565000
8	0.315285000	-0.014893000	0.358971000
16	0.406998000	4.631300000	0.171274000
7	-4.409020000	-0.239503000	-0.233916000
7	5.065099000	-0.293913000	0.344009000
6	-4.336411000	-1.705631000	-0.184855000
1	-3.683567000	-2.005503000	0.638623000
1	-5.336126000	-2.067023000	0.065357000
6	-3.869876000	-2.349453000	-1.493323000
1	-2.880278000	-1.987581000	-1.786388000
1	-4.566997000	-2.133987000	-2.307486000
1	-3.807217000	-3.434557000	-1.371392000
6	-5.708180000	0.379542000	-0.515729000
1	-6.476954000	-0.337283000	-0.220666000
1	-5.838811000	1.254092000	0.129209000
6	-5.898265000	0.767648000	-1.984372000
1	-5.854219000	-0.115061000	-2.627686000
1	-5.124104000	1.467100000	-2.312050000
1	-6.871800000	1.246275000	-2.125952000

Xanthione

6	4.986717000	-1.754969000	0.163689000
1	5.939545000	-2.167216000	0.497158000
1	4.212481000	-2.165903000	0.815144000
6	4.713474000	-2.164606000	-1.286664000
1	5.518448000	-1.837901000	-1.948625000
1	3.773877000	-1.737801000	-1.646837000
1	4.635507000	-3.253065000	-1.346931000
6	6.407287000	0.306288000	0.404409000
1	6.385423000	1.146727000	1.101507000
1	7.073073000	-0.443212000	0.835270000
6	6.936820000	0.751704000	-0.961923000
1	6.262532000	1.471191000	-1.433202000
1	7.054748000	-0.100265000	-1.635204000
1	7.914265000	1.225999000	-0.838182000
6	1.343454000	-3.707588000	-0.470085000
7	2.098136000	-3.554339000	0.406164000
6	0.392348000	-3.860107000	-1.556404000
1	-0.602291000	-4.051135000	-1.146098000
1	0.678312000	-4.690457000	-2.205590000
1	0.359672000	-2.938198000	-2.145127000
6	-0.950581000	-3.090807000	1.850631000
7	-1.665237000	-3.661807000	1.126926000
6	-0.050824000	-2.353994000	2.720630000
1	0.877863000	-2.910721000	2.858898000
1	-0.518544000	-2.176675000	3.690423000
1	0.191107000	-1.394954000	2.255119000
6	-0.639146000	-0.036319000	-3.135539000
7	0.210444000	-0.810262000	-2.942494000
6	-1.703661000	0.923340000	-3.374882000
1	-2.675395000	0.425314000	-3.342686000
1	-1.583769000	1.391205000	-4.354253000
1	-1.692066000	1.701513000	-2.608733000

Xanthione

6	-2.837235000	-0.091495000	3.356798000
7	-3.303622000	-1.160046000	3.332805000
6	-2.246500000	1.236012000	3.381632000
1	-1.161288000	1.170889000	3.275785000
1	-2.475056000	1.739800000	4.323182000
1	-2.634439000	1.837727000	2.556741000

D. S=6

1. S_0

6	2.255575000	-0.424062000	-0.535562000
6	3.452848000	-1.160078000	-0.416792000
6	3.346946000	-2.516341000	0.022430000
6	2.130273000	-3.072338000	0.321425000
6	0.920139000	-2.351407000	0.213699000
6	1.043695000	-1.021085000	-0.238051000
6	-0.363810000	-2.906036000	0.546964000
6	-1.496801000	-2.045914000	0.357851000
6	-1.333611000	-0.733555000	-0.131614000
6	-2.829883000	-2.434122000	0.625934000
6	-3.899896000	-1.608358000	0.392630000
6	-2.389080000	0.118346000	-0.383427000
6	-3.713941000	-0.303656000	-0.160594000
1	-2.991270000	-3.427636000	1.022248000
1	-2.164523000	1.092303000	-0.779025000
1	-4.892578000	-1.967918000	0.617614000
1	2.245130000	0.609525000	-0.845786000
1	2.074044000	-4.098077000	0.660323000
1	4.232739000	-3.122196000	0.134203000
8	-0.074833000	-0.225099000	-0.400254000

Xanthione

16	-0.534016000	-4.534627000	1.144326000
7	4.671605000	-0.612117000	-0.710360000
7	-4.779489000	0.486756000	-0.491254000
6	5.911408000	-1.385210000	-0.545567000
1	5.770423000	-2.384542000	-0.966987000
1	6.672476000	-0.900319000	-1.160011000
6	6.399867000	-1.479291000	0.901527000
1	5.638709000	-1.919143000	1.551714000
1	6.650649000	-0.491248000	1.294982000
1	7.295959000	-2.104610000	0.955361000
6	4.804746000	0.803263000	-1.080412000
1	5.736071000	0.899495000	-1.642813000
1	3.999620000	1.078300000	-1.765819000
6	4.822957000	1.751489000	0.119638000
1	5.633059000	1.495217000	0.806989000
1	3.879290000	1.719899000	0.670562000
1	4.984810000	2.779004000	-0.218843000
6	-6.149776000	0.113357000	-0.115803000
1	-6.821786000	0.727572000	-0.718196000
1	-6.338131000	-0.924879000	-0.407176000
6	-6.455814000	0.315750000	1.369296000
1	-6.346108000	1.366783000	1.647805000
1	-5.781865000	-0.272082000	1.998357000
1	-7.482193000	0.006876000	1.588401000
6	-4.585457000	1.788777000	-1.146447000
1	-3.733902000	1.718268000	-1.827095000
1	-5.463886000	1.966839000	-1.772530000
6	-4.402812000	2.944969000	-0.161362000
1	-3.625425000	2.720603000	0.573578000
1	-5.329681000	3.141636000	0.385793000
1	-4.128558000	3.859261000	-0.697150000
6	-0.558386000	1.085218000	-3.552097000

Xanthione

7	-1.664507000	1.398184000	-3.354048000
6	0.822409000	0.699120000	-3.785813000
1	1.008893000	-0.283672000	-3.347721000
1	1.493074000	1.432752000	-3.331059000
1	1.024850000	0.647020000	-4.858262000
6	-1.462114000	2.296089000	3.501922000
7	-2.231690000	2.304411000	2.627025000
6	-0.490794000	2.298644000	4.582410000
1	-0.691177000	3.117485000	5.276471000
1	0.513791000	2.420557000	4.170519000
1	-0.530001000	1.352723000	5.124773000
6	2.711444000	4.013389000	-1.433398000
7	2.430522000	3.099008000	-2.097682000
6	3.056116000	5.135225000	-0.576883000
1	2.350721000	5.956725000	-0.719783000
1	4.061057000	5.496786000	-0.804666000
1	3.024769000	4.825621000	0.470549000
6	0.724871000	2.724834000	0.921375000
7	1.555388000	2.429892000	1.684678000
6	-0.311234000	3.103736000	-0.022661000
1	-1.289723000	2.934598000	0.430154000
1	-0.226928000	2.506277000	-0.931178000
1	-0.223529000	4.160237000	-0.284882000
6	0.905445000	-0.697825000	3.264889000
7	-0.247023000	-0.817212000	3.399303000
6	2.338829000	-0.520745000	3.106318000
1	2.837418000	-0.564495000	4.077291000
1	2.532399000	0.451715000	2.647718000
1	2.751100000	-1.303018000	2.465541000
6	-1.483799000	-2.214929000	-3.161045000
7	-0.332729000	-2.396463000	-3.119125000
6	-2.914875000	-1.968731000	-3.215447000

Xanthione

1	-3.397043000	-2.337905000	-2.307783000
1	-3.357319000	-2.470683000	-4.078445000
1	-3.099956000	-0.895184000	-3.294673000

2. L_a

6	2.285149000	0.009421000	-0.546304000
6	3.475489000	-0.670731000	-0.900133000
6	3.344671000	-1.980657000	-1.420546000
6	2.101106000	-2.571607000	-1.571136000
6	0.909724000	-1.913803000	-1.212576000
6	1.062051000	-0.603134000	-0.709065000
6	-0.396652000	-2.478610000	-1.307418000
6	-1.533158000	-1.700605000	-0.938934000
6	-1.342288000	-0.376146000	-0.489542000
6	-2.870734000	-2.138230000	-0.998040000
6	-3.934650000	-1.312944000	-0.671331000
6	-2.382969000	0.468721000	-0.176073000
6	-3.723690000	0.028640000	-0.273218000
1	-3.080300000	-3.154107000	-1.315067000
1	-2.140196000	1.465923000	0.153155000
1	-4.934714000	-1.714758000	-0.736244000
1	2.295582000	1.009572000	-0.137641000
1	2.049470000	-3.577101000	-1.974492000
1	4.218445000	-2.545399000	-1.708996000
8	-0.056551000	0.147533000	-0.340427000
16	-0.605143000	-4.162304000	-1.856943000
7	4.713451000	-0.075594000	-0.755367000
7	-4.775483000	0.881582000	-0.007088000
6	5.942653000	-0.829703000	-1.016205000
1	5.851699000	-1.351413000	-1.974692000

Xanthione

1	6.745768000	-0.100052000	-1.141980000
6	6.316250000	-1.819420000	0.090825000
1	5.512740000	-2.540097000	0.266126000
1	6.511789000	-1.296212000	1.030313000
1	7.217509000	-2.375180000	-0.185471000
6	4.843720000	1.269327000	-0.189573000
1	5.794272000	1.677630000	-0.542759000
1	4.060390000	1.907944000	-0.605204000
6	4.799891000	1.311401000	1.339748000
1	5.629795000	0.742545000	1.767741000
1	3.865338000	0.893662000	1.722995000
1	4.878461000	2.343898000	1.693684000
6	-6.153541000	0.385358000	0.023124000
1	-6.809499000	1.254912000	-0.059076000
1	-6.339753000	-0.223656000	-0.868047000
6	-6.501251000	-0.405139000	1.287085000
1	-6.410487000	0.225452000	2.175338000
1	-5.834622000	-1.262733000	1.412660000
1	-7.529175000	-0.776523000	1.232942000
6	-4.536437000	2.272293000	0.390579000
1	-3.729531000	2.681085000	-0.223591000
1	-5.431678000	2.841504000	0.126812000
6	-4.229711000	2.449917000	1.880245000
1	-3.427114000	1.782917000	2.205759000
1	-5.111894000	2.223826000	2.486577000
1	-3.933805000	3.483483000	2.087142000
6	-1.424686000	3.223839000	-2.262434000
7	-2.532186000	3.276578000	-2.625430000
6	-0.054102000	3.138208000	-1.789833000
1	0.057387000	2.285327000	-1.116025000
1	0.230241000	4.044231000	-1.253334000
1	0.627971000	2.992089000	-2.629840000

Xanthione

6	-1.568398000	-0.812601000	4.146478000
7	-2.256784000	-0.165987000	3.463488000
6	-0.700947000	-1.606479000	5.000230000
1	-1.002109000	-1.514109000	6.045789000
1	0.330738000	-1.262370000	4.894753000
1	-0.745148000	-2.655902000	4.704770000
6	3.091043000	4.453405000	0.319463000
7	2.267457000	3.638221000	0.437742000
6	4.133576000	5.453715000	0.165089000
1	4.299442000	5.980885000	1.106935000
1	3.852824000	6.183048000	-0.597643000
1	5.069727000	4.978163000	-0.136664000
6	0.917517000	1.107691000	2.844207000
7	1.659346000	0.296304000	3.232621000
6	-0.006027000	2.118152000	2.361839000
1	-0.918466000	1.636876000	2.009507000
1	0.459450000	2.678387000	1.548180000
1	-0.265556000	2.808724000	3.167688000
6	0.933512000	-3.036179000	2.105730000
7	-0.208991000	-3.269338000	2.139081000
6	2.354314000	-2.733043000	2.091509000
1	2.908428000	-3.477487000	2.667940000
1	2.515475000	-1.745288000	2.530225000
1	2.731985000	-2.728836000	1.066844000
6	-1.222369000	0.264125000	-3.859538000
7	-0.065292000	0.402843000	-3.811350000
6	-2.666557000	0.113291000	-3.914741000
1	-2.993128000	-0.664236000	-3.220879000
1	-2.985258000	-0.157586000	-4.923635000
1	-3.143424000	1.054444000	-3.631016000

Xanthione

3. L_b

6	2.497702000	-0.058013000	-0.510177000
6	3.694625000	-0.823422000	-0.525585000
6	3.565132000	-2.235257000	-0.665335000
6	2.328570000	-2.829687000	-0.757002000
6	1.117741000	-2.089153000	-0.719624000
6	1.280089000	-0.682824000	-0.608743000
6	-0.183676000	-2.690724000	-0.753067000
6	-1.299160000	-1.798200000	-0.640228000
6	-1.108550000	-0.386801000	-0.580340000
6	-2.657632000	-2.230032000	-0.580000000
6	-3.710517000	-1.351104000	-0.507596000
6	-2.138585000	0.510709000	-0.521105000
6	-3.489863000	0.059158000	-0.507243000
1	-2.833685000	-3.296369000	-0.599318000
1	-1.894683000	1.559122000	-0.488950000
1	-4.716029000	-1.744354000	-0.477805000
1	2.508352000	1.018019000	-0.416555000
1	2.248158000	-3.904328000	-0.848981000
1	4.442969000	-2.864079000	-0.682927000
8	0.169602000	0.162054000	-0.586506000
16	-0.415870000	-4.454766000	-0.883028000
7	4.920745000	-0.221845000	-0.409683000
7	-4.524341000	0.947837000	-0.521080000
6	6.160021000	-1.008232000	-0.453809000
1	6.072638000	-1.763180000	-1.239448000
1	6.959066000	-0.330881000	-0.762018000
6	6.522905000	-1.664399000	0.881481000
1	5.717577000	-2.313712000	1.234820000
1	6.714611000	-0.910466000	1.648891000
1	7.426438000	-2.270025000	0.765507000

Xanthione

6	5.035467000	1.209084000	-0.095626000
1	6.050986000	1.513318000	-0.355450000
1	4.359871000	1.778822000	-0.737992000
6	4.753824000	1.532372000	1.374750000
1	5.472999000	1.034780000	2.029935000
1	3.749054000	1.214500000	1.664969000
1	4.831180000	2.610830000	1.538907000
6	-5.902935000	0.510745000	-0.256326000
1	-6.557153000	1.337248000	-0.535930000
1	-6.152793000	-0.325859000	-0.916880000
6	-6.138118000	0.128301000	1.207620000
1	-5.959103000	0.982170000	1.865023000
1	-5.475683000	-0.683455000	1.518097000
1	-7.171975000	-0.201718000	1.341296000
6	-4.305649000	2.388744000	-0.727816000
1	-3.425053000	2.525219000	-1.357237000
1	-5.159933000	2.759935000	-1.300713000
6	-4.175877000	3.169427000	0.582584000
1	-3.418262000	2.724528000	1.231530000
1	-5.122174000	3.176174000	1.131151000
1	-3.902244000	4.206763000	0.368557000
6	-0.298039000	2.455440000	-3.003136000
7	-1.437632000	2.667221000	-2.874236000
6	1.119190000	2.179733000	-3.159046000
1	1.673411000	2.569363000	-2.301875000
1	1.498738000	2.648343000	-4.070188000
1	1.275650000	1.101129000	-3.226149000
6	-2.215151000	0.498736000	3.934157000
7	-2.681561000	1.103227000	3.053606000
6	-1.613478000	-0.239324000	5.031564000
1	-1.779674000	0.278479000	5.978525000
1	-0.538191000	-0.333496000	4.862036000

Xanthione

1	-2.042094000	-1.240891000	5.088489000
6	2.817166000	4.492448000	0.160005000
7	2.556533000	3.521893000	-0.428035000
6	3.141492000	5.696077000	0.905844000
1	2.494102000	5.784830000	1.781126000
1	3.004616000	6.580795000	0.280521000
1	4.179923000	5.665447000	1.243123000
6	0.687505000	1.259070000	2.333952000
7	1.130975000	0.305290000	2.836884000
6	0.125938000	2.445299000	1.712175000
1	-0.962442000	2.414494000	1.793614000
1	0.409349000	2.481970000	0.659785000
1	0.491341000	3.346696000	2.209119000
6	-0.768467000	-2.846536000	2.716413000
7	-1.917664000	-2.674791000	2.819586000
6	0.665238000	-3.048702000	2.599153000
1	1.042774000	-3.601287000	3.462920000
1	1.167879000	-2.079210000	2.553495000
1	0.885175000	-3.614395000	1.690778000
6	-1.168136000	-0.720189000	-3.973486000
7	-0.015613000	-0.896678000	-3.967016000
6	-2.601139000	-0.479127000	-3.972295000
1	-3.094763000	-1.137327000	-3.254065000
1	-3.022245000	-0.662408000	-4.962989000
1	-2.797451000	0.557500000	-3.689133000

Xanthione

V. OPTIMISED GEOMETRIES - DICHLOROETHANE

A. Implicit

1. S_0

6	2.308586000	-0.785804000	-0.357084000
6	3.581726000	-0.205230000	-0.293895000
6	3.639136000	1.205398000	-0.113608000
6	2.501987000	1.951594000	-0.031297000
6	1.215474000	1.386754000	-0.105120000
6	1.175762000	-0.002988000	-0.270945000
6	0.000068000	2.154267000	-0.014507000
6	-1.215372000	1.386765000	-0.104853000
6	-1.175730000	-0.002935000	-0.270858000
6	-2.501866000	1.951645000	-0.030762000
6	-3.639038000	1.205491000	-0.112975000
6	-2.308596000	-0.785731000	-0.356892000
6	-3.581666000	-0.205078000	-0.293792000
1	-2.569406000	3.021908000	0.102687000
1	-2.166450000	-1.847809000	-0.470181000
1	-4.590365000	1.706232000	-0.037528000
1	2.166415000	-1.847881000	-0.470372000
1	2.569588000	3.021882000	0.101924000
1	4.590466000	1.706195000	-0.038477000
8	0.000002000	-0.677234000	-0.349995000
16	0.000102000	3.829339000	0.182967000
7	4.713446000	-0.949391000	-0.413377000
7	-4.713457000	-0.949122000	-0.413661000
6	4.642801000	-2.397793000	-0.558529000
1	3.860846000	-2.643331000	-1.277949000
1	5.579332000	-2.720654000	-1.009987000
6	4.416373000	-3.151127000	0.746608000

Xanthione

1	3.505413000	-2.820331000	1.244742000
1	5.249439000	-3.003851000	1.432939000
1	4.325718000	-4.219955000	0.548431000
6	6.034674000	-0.346354000	-0.279111000
1	6.740531000	-1.017367000	-0.765526000
1	6.063592000	0.583147000	-0.847695000
6	6.469935000	-0.100360000	1.159991000
1	6.544915000	-1.036433000	1.712216000
1	5.765291000	0.545312000	1.683643000
1	7.448191000	0.382039000	1.175798000
6	-4.642893000	-2.397444000	-0.559073000
1	-5.579312000	-2.720159000	-1.010891000
1	-3.860757000	-2.642933000	-1.278314000
6	-4.416969000	-3.151083000	0.745992000
1	-5.250432000	-3.004179000	1.431928000
1	-3.506339000	-2.820212000	1.244686000
1	-4.325994000	-4.219848000	0.547618000
6	-6.034615000	-0.346047000	-0.279032000
1	-6.063538000	0.583643000	-0.847325000
1	-6.740535000	-1.016856000	-0.765635000
6	-6.469811000	-0.100630000	1.160181000
1	-5.765158000	0.544804000	1.684111000
1	-6.544741000	-1.036950000	1.711997000
1	-7.448067000	0.381753000	1.176270000

2. L_a

6	2.321946000	-0.778206000	-0.361377000
6	3.601325000	-0.195000000	-0.305521000
6	3.657380000	1.201541000	-0.134860000
6	2.507488000	1.955226000	-0.049524000

Xanthione

6	1.228355000	1.387652000	-0.113652000
6	1.188908000	-0.006313000	-0.274145000
6	-0.000039000	2.110996000	-0.018586000
6	-1.228421000	1.387626000	-0.113638000
6	-1.188941000	-0.006355000	-0.274101000
6	-2.507556000	1.955159000	-0.049508000
6	-3.657444000	1.201441000	-0.134848000
6	-2.321946000	-0.778272000	-0.361376000
6	-3.601366000	-0.195094000	-0.305530000
1	-2.601691000	3.026861000	0.076073000
1	-2.184719000	-1.841849000	-0.472261000
1	-4.606177000	1.708869000	-0.067709000
1	2.184722000	-1.841794000	-0.472208000
1	2.601577000	3.026934000	0.076048000
1	4.606124000	1.708969000	-0.067811000
8	-0.000004000	-0.682804000	-0.342534000
16	-0.000066000	3.816865000	0.238456000
7	4.736839000	-0.955452000	-0.433528000
7	-4.736852000	-0.955602000	-0.433513000
6	4.657595000	-2.403548000	-0.536136000
1	3.876196000	-2.668917000	-1.250125000
1	5.593207000	-2.745662000	-0.976772000
6	4.424132000	-3.125524000	0.786776000
1	3.514558000	-2.776033000	1.274853000
1	5.256643000	-2.966540000	1.471426000
1	4.326252000	-4.198740000	0.615820000
6	6.054263000	-0.354317000	-0.302554000
1	6.762885000	-1.027592000	-0.783386000
1	6.083138000	0.571980000	-0.878392000
6	6.495828000	-0.091279000	1.133153000
1	6.585210000	-1.022206000	1.692214000
1	5.785177000	0.548970000	1.655360000

Xanthione

1	7.468290000	0.403577000	1.141587000
6	-4.657565000	-2.403700000	-0.535764000
1	-5.593373000	-2.745991000	-0.975830000
1	-3.876510000	-2.669226000	-1.250087000
6	-4.423402000	-3.125297000	0.787238000
1	-5.255633000	-2.966244000	1.472211000
1	-3.513670000	-2.775533000	1.274824000
1	-4.325426000	-4.198543000	0.616539000
6	-6.054290000	-0.354428000	-0.302810000
1	-6.082932000	0.571918000	-0.878566000
1	-6.762832000	-1.027606000	-0.783902000
6	-6.496210000	-0.091507000	1.132810000
1	-5.785564000	0.548521000	1.655299000
1	-6.585944000	-1.022494000	1.691716000
1	-7.468575000	0.403544000	1.141060000

3. L_b

6	2.326633000	-0.779796000	-0.369145000
6	3.602553000	-0.191804000	-0.301554000
6	3.648273000	1.206286000	-0.121424000
6	2.497696000	1.955075000	-0.038173000
6	1.218416000	1.385306000	-0.113845000
6	1.195558000	-0.005464000	-0.283905000
6	-0.008153000	2.117679000	-0.021401000
6	-1.217233000	1.382288000	-0.106203000
6	-1.178360000	-0.036341000	-0.284404000
6	-2.515602000	1.957534000	-0.023248000
6	-3.652198000	1.203807000	-0.111823000
6	-2.301574000	-0.797899000	-0.374144000
6	-3.592634000	-0.202738000	-0.297143000

Xanthione

1	-2.574888000	3.025677000	0.117890000
1	-2.173112000	-1.860198000	-0.500821000
1	-4.605179000	1.701658000	-0.035479000
1	2.191690000	-1.842776000	-0.487690000
1	2.565274000	3.025118000	0.098226000
1	4.595257000	1.715929000	-0.043234000
8	0.005997000	-0.692224000	-0.367547000
16	-0.070622000	3.848560000	0.197778000
7	4.742347000	-0.944930000	-0.424803000
7	-4.711766000	-0.955763000	-0.406808000
6	4.671221000	-2.392942000	-0.534054000
1	3.897200000	-2.658934000	-1.255892000
1	5.612362000	-2.728918000	-0.967410000
6	4.429259000	-3.120884000	0.784140000
1	3.513818000	-2.777230000	1.265285000
1	5.254853000	-2.960675000	1.476767000
1	4.337913000	-4.193859000	0.608128000
6	6.056436000	-0.337523000	-0.285733000
1	6.770096000	-1.003869000	-0.768765000
1	6.081720000	0.592775000	-0.854854000
6	6.491077000	-0.082189000	1.153395000
1	6.582349000	-1.016345000	1.706756000
1	5.775539000	0.551766000	1.676559000
1	7.461316000	0.416793000	1.168730000
6	-4.642308000	-2.406382000	-0.561177000
1	-5.581405000	-2.722602000	-1.009743000
1	-3.861362000	-2.648313000	-1.280701000
6	-4.415056000	-3.157389000	0.746015000
1	-5.242587000	-3.003808000	1.436813000
1	-3.496859000	-2.834755000	1.235481000
1	-4.336525000	-4.225700000	0.542151000
6	-6.038964000	-0.360402000	-0.275549000

Xanthione

1	-6.066835000	0.567650000	-0.845442000
1	-6.739610000	-1.038917000	-0.756950000
6	-6.466151000	-0.114079000	1.167111000
1	-5.760885000	0.534157000	1.685702000
1	-6.541055000	-1.048891000	1.720406000
1	-7.444531000	0.367097000	1.178890000

B. S=2

1. S_0

6	2.294714000	0.108385000	-0.553854000
6	3.567352000	0.708975000	-0.656307000
6	3.607864000	2.125182000	-0.836965000
6	2.456336000	2.863936000	-0.915313000
6	1.172442000	2.282521000	-0.812726000
6	1.154887000	0.887259000	-0.630952000
6	-0.050201000	3.041609000	-0.860962000
6	-1.271198000	2.291185000	-0.716751000
6	-1.246594000	0.895284000	-0.542601000
6	-2.557416000	2.877792000	-0.729627000
6	-3.705724000	2.141586000	-0.592360000
6	-2.383416000	0.122589000	-0.396384000
6	-3.658523000	0.722390000	-0.439563000
1	-2.615051000	3.950895000	-0.854062000
1	-2.260770000	-0.938559000	-0.253563000
1	-4.655088000	2.654797000	-0.608419000
1	2.173867000	-0.954935000	-0.410888000
1	2.509478000	3.935913000	-1.050726000
1	4.555635000	2.636373000	-0.907402000
8	-0.044620000	0.206115000	-0.489122000

Xanthione

16	-0.053224000	4.763391000	-1.079742000
7	4.716471000	-0.030151000	-0.593156000
7	-4.801830000	-0.023039000	-0.353559000
6	4.671629000	-1.477680000	-0.348235000
1	3.883902000	-1.922278000	-0.961861000
1	5.614143000	-1.894538000	-0.709069000
6	4.469729000	-1.849157000	1.121670000
1	3.543872000	-1.422245000	1.516293000
1	5.298604000	-1.485871000	1.734312000
1	4.413179000	-2.935806000	1.230424000
6	6.038477000	0.610149000	-0.649504000
1	6.752783000	-0.159416000	-0.948972000
1	6.042177000	1.357325000	-1.448105000
6	6.478100000	1.238042000	0.674318000
1	6.570779000	0.478485000	1.454683000
1	5.759959000	1.988154000	1.016310000
1	7.450322000	1.725455000	0.555620000
6	-4.746167000	-1.487781000	-0.254416000
1	-5.686440000	-1.873173000	-0.655085000
1	-3.958102000	-1.857228000	-0.916467000
6	-4.539677000	-2.004364000	1.169939000
1	-5.373439000	-1.715605000	1.815263000
1	-3.618726000	-1.611529000	1.607684000
1	-4.472031000	-3.096415000	1.168909000
6	-6.123419000	0.616042000	-0.279220000
1	-6.197491000	1.387438000	-1.051705000
1	-6.860326000	-0.146654000	-0.537613000
6	-6.445831000	1.202313000	1.096132000
1	-5.707367000	1.952279000	1.392175000
1	-6.457727000	0.419967000	1.859218000
1	-7.429481000	1.680835000	1.080772000
6	-0.689476000	-0.557383000	2.803761000

Xanthione

1	-1.139222000	-0.408066000	3.781084000
1	-1.272802000	-0.073044000	2.026251000
6	0.767156000	-0.183168000	2.760107000
1	1.340939000	-0.611509000	3.577009000
1	1.226066000	-0.402615000	1.801640000
6	-0.490132000	-3.011224000	-1.284774000
1	-1.034743000	-3.566447000	-0.526346000
1	-0.258546000	-2.005806000	-0.944079000
6	0.717759000	-3.748463000	-1.795691000
1	1.265889000	-3.188530000	-2.548418000
1	0.491520000	-4.752510000	-2.142616000
17	-0.816596000	-2.399600000	2.453975000
17	0.888800000	1.678393000	2.956235000
17	-1.681741000	-2.797989000	-2.723027000
17	1.902375000	-3.959163000	-0.352166000

2. L_a

6	-2.306204000	0.102003000	0.527515000
6	-3.576890000	0.714279000	0.646390000
6	-3.601101000	2.111875000	0.868116000
6	-2.431470000	2.843221000	0.969926000
6	-1.159332000	2.250297000	0.849902000
6	-1.157739000	0.857219000	0.630209000
6	0.082902000	2.950495000	0.909298000
6	1.308299000	2.233711000	0.763653000
6	1.269384000	0.839553000	0.555890000
6	2.595890000	2.805908000	0.797420000
6	3.746198000	2.051056000	0.649077000
6	2.398214000	0.064774000	0.401922000
6	3.684242000	0.649313000	0.464359000

Xanthione

1	2.694325000	3.875645000	0.945339000
1	2.266327000	-0.992211000	0.233639000
1	4.699768000	2.556446000	0.684695000
1	-2.199642000	-0.958053000	0.349345000
1	-2.502395000	3.911884000	1.139237000
1	-4.541156000	2.635546000	0.955472000
8	0.047217000	0.164924000	0.470270000
16	0.103191000	4.721967000	1.130246000
7	-4.738760000	-0.025814000	0.560499000
7	4.823347000	-0.123470000	0.364833000
6	-4.698522000	-1.458188000	0.254153000
1	-3.922095000	-1.936505000	0.858107000
1	-5.648412000	-1.885441000	0.584231000
6	-4.478679000	-1.775397000	-1.226925000
1	-3.545960000	-1.336983000	-1.592332000
1	-5.298068000	-1.384015000	-1.835484000
1	-4.424251000	-2.857371000	-1.377974000
6	-6.049976000	0.625319000	0.626777000
1	-6.777789000	-0.143429000	0.896990000
1	-6.050890000	1.348420000	1.448688000
6	-6.477722000	1.304202000	-0.677029000
1	-6.582558000	0.571222000	-1.481228000
1	-5.743774000	2.050216000	-0.993681000
1	-7.440941000	1.806641000	-0.545489000
6	4.738987000	-1.581290000	0.241323000
1	5.675008000	-1.993375000	0.627110000
1	3.949225000	-1.949107000	0.903727000
6	4.510758000	-2.077268000	-1.188344000
1	5.344567000	-1.794269000	-1.836525000
1	3.594253000	-1.660164000	-1.613432000
1	4.422504000	-3.168049000	-1.202716000
6	6.147544000	0.498991000	0.284724000

Xanthione

1	6.242249000	1.256417000	1.070388000
1	6.881017000	-0.275824000	0.518443000
6	6.462823000	1.111697000	-1.081924000
1	5.723712000	1.869651000	-1.355408000
1	6.463283000	0.343984000	-1.860183000
1	7.448706000	1.586304000	-1.067627000
6	0.731935000	-0.491656000	-2.777600000
1	1.220418000	-0.383011000	-3.741693000
1	1.328449000	-0.053819000	-1.982938000
6	-0.689422000	0.001441000	-2.776114000
1	-1.270825000	-0.369585000	-3.615387000
1	-1.194884000	-0.183987000	-1.834124000
6	0.407718000	-3.012074000	1.273170000
1	0.982205000	-3.620872000	0.580831000
1	0.221784000	-2.020901000	0.868303000
6	-0.844740000	-3.694084000	1.752748000
1	-1.429209000	-3.073938000	2.427041000
1	-0.660782000	-4.674175000	2.183225000
17	0.698557000	-2.336364000	-2.421166000
17	-0.644837000	1.869167000	-2.958197000
17	1.519340000	-2.746405000	2.766981000
17	-1.939157000	-3.990842000	0.253920000

3. L_b

6	2.385815000	0.113327000	-0.626723000
6	3.705074000	0.666556000	-0.649287000
6	3.823842000	2.087641000	-0.682968000
6	2.714466000	2.896201000	-0.676245000
6	1.389307000	2.366556000	-0.637623000
6	1.295258000	0.936467000	-0.629018000

Xanthione

6	0.212388000	3.166904000	-0.590105000
6	-1.052576000	2.498752000	-0.541767000
6	-1.122644000	1.091755000	-0.567612000
6	-2.298238000	3.158081000	-0.467936000
6	-3.498532000	2.477701000	-0.443758000
6	-2.304555000	0.381265000	-0.551723000
6	-3.544000000	1.061882000	-0.510519000
1	-2.291593000	4.239362000	-0.433173000
1	-2.254440000	-0.695683000	-0.574227000
1	-4.412925000	3.049568000	-0.386889000
1	2.221297000	-0.953613000	-0.611178000
1	2.816515000	3.971782000	-0.686254000
1	4.798717000	2.551128000	-0.693455000
8	0.051329000	0.319146000	-0.602284000
16	0.364210000	4.956232000	-0.590133000
7	4.804300000	-0.141096000	-0.640523000
7	-4.740847000	0.378238000	-0.551411000
6	4.689859000	-1.594064000	-0.437610000
1	3.892286000	-1.988601000	-1.071206000
1	5.620479000	-2.040104000	-0.790797000
6	4.446061000	-1.978725000	1.024284000
1	3.525993000	-1.527009000	1.403574000
1	5.273575000	-1.654812000	1.659082000
1	4.353402000	-3.064627000	1.108784000
6	6.162714000	0.420689000	-0.710741000
1	6.810230000	-0.359628000	-1.114232000
1	6.168473000	1.238166000	-1.435388000
6	6.699642000	0.895206000	0.642701000
1	6.797066000	0.061345000	1.341510000
1	6.040540000	1.642361000	1.091728000
1	7.687735000	1.344349000	0.508938000
6	-4.780689000	-1.073866000	-0.743523000

Xanthione

1	-5.729291000	-1.310675000	-1.232905000
1	-3.994799000	-1.360510000	-1.447059000
6	-4.655021000	-1.880954000	0.549727000
1	-5.491270000	-1.674352000	1.223336000
1	-3.728624000	-1.644774000	1.078621000
1	-4.652236000	-2.952921000	0.328882000
6	-6.011609000	1.073980000	-0.326194000
1	-6.057304000	1.967971000	-0.957556000
1	-6.805409000	0.411203000	-0.677203000
6	-6.260303000	1.445865000	1.137397000
1	-5.463723000	2.087681000	1.523244000
1	-6.305717000	0.550082000	1.762366000
1	-7.208627000	1.982630000	1.236647000
6	-1.040806000	-0.896841000	2.703620000
1	-1.380067000	-0.665952000	3.709242000
1	-1.641994000	-0.383643000	1.957530000
6	0.434836000	-0.672852000	2.512254000
1	1.042132000	-1.195587000	3.245567000
1	0.759029000	-0.893536000	1.499830000
6	-0.655778000	-2.910844000	-1.264345000
1	-1.192227000	-3.177148000	-0.357791000
1	-0.316299000	-1.879003000	-1.224241000
6	0.446604000	-3.879749000	-1.593942000
1	0.980790000	-3.621117000	-2.503852000
1	0.115472000	-4.913974000	-1.614426000
17	-1.375551000	-2.731669000	2.462738000
17	0.769784000	1.154158000	2.769007000
17	-1.917590000	-2.985977000	-2.654905000
17	1.714765000	-3.775265000	-0.210556000

Xanthione

C. S=4

1. S_0

6	-2.658523000	0.557198000	0.133223000
6	-3.779865000	1.240663000	-0.381148000
6	-3.562513000	2.100960000	-1.500854000
6	-2.308494000	2.282517000	-2.023558000
6	-1.169651000	1.621601000	-1.509940000
6	-1.414362000	0.754790000	-0.427768000
6	0.159990000	1.811896000	-2.021093000
6	1.197648000	1.017882000	-1.410518000
6	0.914302000	0.161438000	-0.329340000
6	2.550174000	1.043926000	-1.822627000
6	3.523151000	0.298376000	-1.206085000
6	1.869389000	-0.594141000	0.322023000
6	3.218022000	-0.538320000	-0.088199000
1	2.808375000	1.686517000	-2.653828000
1	1.548554000	-1.234702000	1.128037000
1	4.535598000	0.367288000	-1.572480000
1	-2.731576000	-0.118916000	0.969995000
1	-2.163618000	2.950738000	-2.861847000
1	-4.390909000	2.628684000	-1.948105000
8	-0.379309000	0.030854000	0.149556000
16	0.514090000	2.956613000	-3.275320000
7	-5.020409000	1.099192000	0.176074000
7	4.192993000	-1.240659000	0.558931000
6	-5.228081000	0.232533000	1.343643000
1	-4.397567000	0.375331000	2.039614000
1	-6.122627000	0.591241000	1.857165000
6	-5.387164000	-1.247273000	0.992022000
1	-4.539791000	-1.607420000	0.403157000

Xanthione

1	-6.294664000	-1.416155000	0.406409000
1	-5.456081000	-1.847370000	1.904765000
6	-6.203182000	1.732641000	-0.423935000
1	-6.974206000	1.762339000	0.348319000
1	-5.972307000	2.774210000	-0.666132000
6	-6.732916000	0.999513000	-1.657326000
1	-7.045837000	-0.015978000	-1.401535000
1	-5.970127000	0.931392000	-2.437648000
1	-7.596079000	1.530297000	-2.069686000
6	3.892473000	-2.039455000	1.755658000
1	4.803449000	-2.069181000	2.358271000
1	3.149579000	-1.508354000	2.355541000
6	3.425297000	-3.462167000	1.449226000
1	4.207716000	-4.029055000	0.937735000
1	2.539433000	-3.458069000	0.810278000
1	3.174920000	-3.984197000	2.376983000
6	5.565442000	-1.294105000	0.033547000
1	5.905460000	-0.280384000	-0.199663000
1	6.201825000	-1.650498000	0.845382000
6	5.718872000	-2.206750000	-1.184123000
1	5.068444000	-1.891314000	-2.004277000
1	5.462896000	-3.238560000	-0.931401000
1	6.752474000	-2.186428000	-1.541747000
6	1.180622000	-2.210013000	-3.233793000
1	1.434796000	-2.599263000	-4.215405000
1	1.741879000	-1.306589000	-3.012665000
6	-0.302959000	-2.046453000	-3.039014000
1	-0.853589000	-2.970215000	-3.188737000
1	-0.559906000	-1.604312000	-2.081397000
6	0.260812000	0.314037000	3.484316000
1	0.225773000	-0.738300000	3.749536000
1	0.094631000	0.453227000	2.420420000

Xanthione

6	-0.649111000	1.165283000	4.325859000
1	-0.637799000	2.209741000	4.028152000
1	-0.476068000	1.053674000	5.392312000
17	1.786398000	-3.498716000	-2.007042000
17	-0.931151000	-0.853356000	-4.339610000
17	2.029204000	0.862905000	3.810454000
17	-2.407367000	0.566216000	4.030945000
6	-0.836703000	-3.133637000	0.898377000
1	-0.123574000	-3.648294000	0.259981000
1	-0.841100000	-2.066121000	0.697050000
6	-2.210458000	-3.745876000	0.858272000
1	-2.210129000	-4.811710000	1.067209000
1	-2.925553000	-3.224039000	1.488629000
6	2.476033000	4.157873000	-0.070081000
1	2.250853000	5.203123000	0.121995000
1	2.004429000	3.820289000	-0.989977000
6	2.182112000	3.269459000	1.108628000
1	2.421982000	2.226365000	0.925968000
1	2.640113000	3.614477000	2.030836000
17	-0.173518000	-3.308304000	2.649950000
17	-2.853725000	-3.567004000	-0.897928000
17	4.327445000	4.085159000	-0.385575000
17	0.326758000	3.318849000	1.413164000

2. L_a

6	-2.655171000	-0.509730000	-0.212750000
6	-3.789925000	-1.262919000	0.170651000
6	-3.585172000	-2.313208000	1.097773000
6	-2.322167000	-2.612378000	1.575179000
6	-1.179742000	-1.888093000	1.183653000

Xanthione

6	-1.413103000	-0.824447000	0.287481000
6	0.152477000	-2.161913000	1.613504000
6	1.225134000	-1.335571000	1.164842000
6	0.954360000	-0.271573000	0.276677000
6	2.577603000	-1.490195000	1.531966000
6	3.575375000	-0.662190000	1.046208000
6	1.927501000	0.566237000	-0.221063000
6	3.284314000	0.385361000	0.139165000
1	2.851505000	-2.286393000	2.214921000
1	1.621111000	1.371805000	-0.870178000
1	4.590641000	-0.842686000	1.365223000
1	-2.724623000	0.316077000	-0.903603000
1	-2.213168000	-3.429734000	2.279320000
1	-4.418273000	-2.904987000	1.446105000
8	-0.354474000	-0.010340000	-0.139732000
16	0.488786000	-3.564357000	2.665964000
7	-5.038366000	-0.991225000	-0.350694000
7	4.275655000	1.187674000	-0.380840000
6	-5.227757000	0.079637000	-1.332572000
1	-4.400739000	0.055508000	-2.047158000
1	-6.129694000	-0.157949000	-1.902272000
6	-5.357869000	1.474651000	-0.716860000
1	-4.505210000	1.700242000	-0.071340000
1	-6.263581000	1.552323000	-0.109133000
1	-5.410039000	2.234722000	-1.503131000
6	-6.228138000	-1.688160000	0.145414000
1	-7.013499000	-1.553417000	-0.601739000
1	-6.028112000	-2.764146000	0.187467000
6	-6.718972000	-1.186585000	1.505669000
1	-7.012039000	-0.135026000	1.448326000
1	-5.938601000	-1.278133000	2.266003000
1	-7.586336000	-1.767003000	1.834754000

Xanthione

6	3.983061000	2.197950000	-1.402172000
1	4.886478000	2.317035000	-2.006719000
1	3.218599000	1.805479000	-2.077742000
6	3.557614000	3.554609000	-0.838816000
1	4.359384000	3.996717000	-0.240807000
1	2.675905000	3.458444000	-0.201074000
1	3.317189000	4.244940000	-1.652766000
6	5.641977000	1.125799000	0.147549000
1	5.979809000	0.083789000	0.170315000
1	6.285344000	1.638957000	-0.570255000
6	5.798382000	1.765346000	1.529238000
1	5.140610000	1.291568000	2.262890000
1	5.549901000	2.829090000	1.494046000
1	6.829809000	1.663425000	1.879943000
6	1.404783000	1.470506000	3.544273000
1	1.717624000	1.643036000	4.570066000
1	1.962224000	0.654031000	3.093485000
6	-0.086462000	1.311757000	3.410762000
1	-0.637226000	2.179437000	3.761078000
1	-0.398060000	1.043411000	2.405991000
6	0.258848000	0.548280000	-3.429633000
1	0.367273000	1.628149000	-3.394781000
1	0.092411000	0.139391000	-2.437371000
6	-0.777501000	0.095314000	-4.420394000
1	-0.921620000	-0.981244000	-4.412522000
1	-0.600186000	0.464964000	-5.426179000
17	1.911298000	3.015465000	2.600446000
17	-0.617334000	-0.110253000	4.510524000
17	1.925684000	-0.112384000	-3.995576000
17	-2.422359000	0.839099000	-3.890919000
6	-0.766950000	3.228600000	-0.167487000
1	-0.048444000	3.564784000	0.574889000

Xanthione

1	-0.754631000	2.145535000	-0.259937000
6	-2.146567000	3.785454000	0.055487000
1	-2.163258000	4.868480000	0.136435000
1	-2.867841000	3.436786000	-0.678835000
6	2.097830000	-4.181713000	-0.934622000
1	1.811647000	-5.129741000	-1.381157000
1	1.609065000	-4.038968000	0.024712000
6	1.908878000	-3.015176000	-1.865816000
1	2.198867000	-2.066591000	-1.423939000
1	2.384666000	-3.154494000	-2.831893000
17	-0.139621000	3.888619000	-1.814355000
17	-2.752992000	3.138952000	1.712714000
17	3.932680000	-4.328003000	-0.559166000
17	0.067554000	-2.873509000	-2.218262000

3. L_b

6	-2.632430000	0.489374000	0.225255000
6	-3.772341000	1.291235000	-0.095005000
6	-3.570485000	2.409282000	-0.959361000
6	-2.321214000	2.726840000	-1.429061000
6	-1.166007000	1.957378000	-1.098246000
6	-1.404159000	0.814685000	-0.273524000
6	0.158044000	2.284779000	-1.528314000
6	1.217290000	1.413226000	-1.117521000
6	0.958776000	0.282541000	-0.311862000
6	2.578154000	1.605490000	-1.450710000
6	3.578882000	0.767991000	-1.002187000
6	1.932974000	-0.573831000	0.150967000
6	3.293915000	-0.343829000	-0.166105000
1	2.826684000	2.455159000	-2.072271000

Xanthione

1	1.629120000	-1.428587000	0.735136000
1	4.596487000	0.985918000	-1.289612000
1	-2.710771000	-0.381274000	0.856950000
1	-2.173324000	3.587747000	-2.065048000
1	-4.403550000	3.037119000	-1.237720000
8	-0.357288000	-0.040784000	0.061390000
16	0.437651000	3.749914000	-2.509706000
7	-5.000039000	0.999871000	0.418975000
7	4.293397000	-1.152160000	0.322312000
6	-5.186792000	-0.065073000	1.416688000
1	-4.315131000	-0.090116000	2.071444000
1	-6.036660000	0.224589000	2.038190000
6	-5.437331000	-1.440585000	0.794426000
1	-4.634344000	-1.715278000	0.106096000
1	-6.378764000	-1.458149000	0.239583000
1	-5.494760000	-2.196137000	1.583548000
6	-6.207666000	1.698432000	-0.050922000
1	-6.996393000	1.489190000	0.672155000
1	-6.033534000	2.778292000	-0.023179000
6	-6.650804000	1.258715000	-1.449123000
1	-6.902075000	0.195698000	-1.461766000
1	-5.866653000	1.433082000	-2.189709000
1	-7.536798000	1.825330000	-1.748271000
6	4.012654000	-2.233774000	1.271747000
1	4.900770000	-2.352450000	1.898490000
1	3.210554000	-1.915900000	1.942117000
6	3.665283000	-3.567745000	0.609343000
1	4.504234000	-3.935909000	0.012482000
1	2.800006000	-3.466657000	-0.049514000
1	3.430658000	-4.318041000	1.369875000
6	5.672423000	-1.013970000	-0.159586000
1	5.975566000	0.037067000	-0.103371000

Xanthione

1	6.310531000	-1.554877000	0.542058000
6	5.886744000	-1.554666000	-1.575145000
1	5.236806000	-1.051229000	-2.295486000
1	5.670030000	-2.624870000	-1.618684000
1	6.924642000	-1.399904000	-1.884750000
6	1.329701000	-1.288605000	-3.614748000
1	1.564619000	-1.355051000	-4.673090000
1	1.814178000	-0.431285000	-3.155586000
6	-0.148146000	-1.351263000	-3.337348000
1	-0.612748000	-2.253910000	-3.722719000
1	-0.384774000	-1.220862000	-2.286303000
6	0.295363000	-0.728820000	3.322139000
1	0.456478000	-1.798957000	3.232486000
1	0.137086000	-0.273610000	2.349456000
6	-0.793045000	-0.383942000	4.300241000
1	-1.003638000	0.681122000	4.337260000
1	-0.624343000	-0.790531000	5.293066000
17	2.115073000	-2.803979000	-2.824812000
17	-0.979938000	0.071681000	-4.227728000
17	1.909732000	-0.021722000	3.970830000
17	-2.374239000	-1.201732000	3.685062000
6	-0.703154000	-3.303343000	0.006533000
1	-0.063234000	-3.631744000	-0.808360000
1	-0.654447000	-2.225236000	0.133739000
6	-2.114595000	-3.809442000	-0.111442000
1	-2.175162000	-4.885612000	-0.244183000
1	-2.750698000	-3.479827000	0.705939000
6	2.047986000	4.166072000	1.033085000
1	1.704521000	5.092507000	1.484775000
1	1.613318000	4.026811000	0.045559000
6	1.871465000	2.974995000	1.935781000
1	2.206177000	2.046007000	1.484089000

Xanthione

1	2.313656000	3.110681000	2.918274000
17	0.046747000	-4.039125000	1.565811000
17	-2.849552000	-3.054337000	-1.668466000
17	3.892626000	4.392273000	0.750457000
17	0.026559000	2.758734000	2.239202000

D. S=6

1. S_0

6	1.512712000	-1.111936000	0.730949000
6	1.778682000	-2.344307000	1.364778000
6	0.948959000	-2.699458000	2.473549000
6	-0.055570000	-1.869818000	2.904969000
6	-0.335070000	-0.631651000	2.284755000
6	0.496588000	-0.299553000	1.197017000
6	-1.400589000	0.244665000	2.705173000
6	-1.570132000	1.448767000	1.931970000
6	-0.709509000	1.748523000	0.858901000
6	-2.603156000	2.387001000	2.162538000
6	-2.772622000	3.504089000	1.384107000
6	-0.846045000	2.867748000	0.063334000
6	-1.884807000	3.788938000	0.302046000
1	-3.285137000	2.188512000	2.978471000
1	-0.151788000	2.995874000	-0.748968000
1	-3.596234000	4.166814000	1.601277000
1	2.068226000	-0.783662000	-0.134211000
1	-0.669711000	-2.156109000	3.748304000
1	1.109977000	-3.631106000	2.994002000
8	0.337906000	0.903552000	0.526562000
16	-2.426927000	-0.137167000	4.045221000

Xanthione

7	2.791940000	-3.161932000	0.953534000
7	-2.033252000	4.905800000	-0.473454000
6	3.728990000	-2.755893000	-0.102230000
1	3.886271000	-1.677778000	-0.032547000
1	4.689922000	-3.224660000	0.123232000
6	3.287145000	-3.139315000	-1.513031000
1	2.340088000	-2.661734000	-1.774094000
1	3.159781000	-4.221482000	-1.603249000
1	4.044080000	-2.825847000	-2.236918000
6	2.936966000	-4.522482000	1.492438000
1	3.931792000	-4.869591000	1.207851000
1	2.925462000	-4.487846000	2.586585000
6	1.881753000	-5.501399000	0.975005000
1	1.936376000	-5.592167000	-0.112443000
1	0.871131000	-5.174936000	1.234596000
1	2.040787000	-6.491354000	1.412376000
6	-1.088128000	5.198113000	-1.560333000
1	-1.156026000	6.268567000	-1.763678000
1	-0.070112000	5.016273000	-1.203834000
6	-1.350416000	4.414268000	-2.848139000
1	-2.347311000	4.631037000	-3.241224000
1	-1.272010000	3.335293000	-2.688069000
1	-0.617220000	4.692373000	-3.611093000
6	-3.177526000	5.811152000	-0.295812000
1	-3.323307000	6.005592000	0.770638000
1	-2.900878000	6.766979000	-0.744715000
6	-4.469639000	5.293082000	-0.928456000
1	-4.754111000	4.321408000	-0.516274000
1	-4.354480000	5.180878000	-2.010037000
1	-5.288651000	5.994632000	-0.743925000
6	-1.930237000	-2.746530000	0.003086000
1	-1.268277000	-3.233035000	0.712937000

Xanthione

1	-1.662776000	-1.702436000	-0.125330000
6	-2.044620000	-3.477507000	-1.306120000
1	-2.341084000	-4.515939000	-1.187313000
1	-2.684537000	-2.964212000	-2.018225000
6	2.558844000	2.423160000	-1.551084000
1	2.066469000	2.620834000	-2.499660000
1	1.947179000	1.783009000	-0.920369000
6	3.969994000	1.928057000	-1.710693000
1	4.485703000	1.806120000	-0.763440000
1	4.559774000	2.523827000	-2.400598000
17	-3.627007000	-2.743831000	0.814872000
17	-0.336818000	-3.516176000	-2.079104000
17	2.647440000	4.077539000	-0.659907000
17	3.889501000	0.203889000	-2.455003000
6	-6.977909000	-2.064128000	-0.795628000
1	-6.281279000	-1.543812000	-0.145496000
1	-7.633948000	-1.361822000	-1.301451000
6	-6.298205000	-3.021452000	-1.737439000
1	-5.628957000	-3.706428000	-1.225165000
1	-6.994690000	-3.553031000	-2.379078000
6	2.777387000	0.831540000	3.684706000
1	3.542169000	0.746192000	4.451318000
1	2.346951000	-0.138657000	3.452515000
6	3.254893000	1.569304000	2.462598000
1	2.496269000	1.636173000	1.688427000
1	3.665416000	2.548425000	2.691282000
17	-8.075351000	-3.074592000	0.342189000
17	-5.205762000	-2.020766000	-2.898536000
17	1.392761000	1.841900000	4.443996000
17	4.676129000	0.590675000	1.712691000
6	7.133632000	-1.038773000	-0.840046000
1	6.110119000	-0.869939000	-0.517882000

Xanthione

1	7.820264000	-1.003751000	0.000848000
6	7.541509000	-0.134781000	-1.971925000
1	8.556313000	-0.315634000	-2.314119000
1	6.835404000	-0.155400000	-2.796871000
6	-3.959265000	0.666242000	-0.728023000
1	-4.125618000	-0.403918000	-0.661451000
1	-3.351789000	1.019144000	0.097988000
6	-3.432332000	1.085901000	-2.071927000
1	-3.226638000	2.150580000	-2.133990000
1	-4.063003000	0.756088000	-2.892160000
17	-5.642957000	1.459375000	-0.488970000
17	-1.780353000	0.225140000	-2.323604000
17	7.185916000	-2.805754000	-1.474049000
17	7.524545000	1.630921000	-1.330315000

2. *L_a*

6	1.516465000	-1.161147000	0.664389000
6	1.788901000	-2.405299000	1.282439000
6	0.961974000	-2.780126000	2.370315000
6	-0.056228000	-1.954930000	2.818018000
6	-0.331849000	-0.709107000	2.220374000
6	0.501715000	-0.358016000	1.135533000
6	-1.376895000	0.176887000	2.618015000
6	-1.586003000	1.389619000	1.896286000
6	-0.736607000	1.704319000	0.813883000
6	-2.620007000	2.315153000	2.146473000
6	-2.801421000	3.449272000	1.373358000
6	-0.898491000	2.821386000	0.027640000
6	-1.939638000	3.741514000	0.289150000
1	-3.310346000	2.128236000	2.961229000

Xanthione

1	-0.220826000	2.957538000	-0.798475000
1	-3.625262000	4.105229000	1.610866000
1	2.075690000	-0.819766000	-0.193740000
1	-0.661305000	-2.284995000	3.654735000
1	1.119834000	-3.720526000	2.876681000
8	0.330582000	0.864529000	0.477554000
16	-2.429560000	-0.236849000	3.996685000
7	2.821340000	-3.209877000	0.853749000
7	-2.096244000	4.873412000	-0.484331000
6	3.761240000	-2.766664000	-0.179664000
1	3.915116000	-1.690532000	-0.073029000
1	4.724194000	-3.238735000	0.033492000
6	3.332104000	-3.099933000	-1.607974000
1	2.377451000	-2.628860000	-1.853193000
1	3.221304000	-4.179856000	-1.740731000
1	4.085474000	-2.746504000	-2.317580000
6	2.950318000	-4.586683000	1.340996000
1	3.945234000	-4.933331000	1.053879000
1	2.927684000	-4.595042000	2.436606000
6	1.891980000	-5.540234000	0.781171000
1	1.960859000	-5.598039000	-0.308040000
1	0.882126000	-5.208346000	1.037566000
1	2.032572000	-6.545427000	1.189786000
6	-1.159672000	5.179765000	-1.569884000
1	-1.214186000	6.256372000	-1.747688000
1	-0.139402000	4.977619000	-1.229386000
6	-1.441149000	4.431859000	-2.875523000
1	-2.436581000	4.676069000	-3.257015000
1	-1.383371000	3.348418000	-2.738385000
1	-0.707683000	4.712627000	-3.637742000
6	-3.243241000	5.764759000	-0.288514000
1	-3.372871000	5.960766000	0.781056000

Xanthione

1	-2.986896000	6.723479000	-0.744648000
6	-4.546483000	5.237192000	-0.892510000
1	-4.812942000	4.262724000	-0.474690000
1	-4.453979000	5.127567000	-1.976770000
1	-5.368315000	5.930610000	-0.689102000
6	-1.751573000	-2.581499000	-0.282310000
1	-0.988173000	-3.037129000	0.341419000
1	-1.576969000	-1.516469000	-0.395816000
6	-1.946445000	-3.278096000	-1.600860000
1	-2.149199000	-4.339997000	-1.493073000
1	-2.694060000	-2.795242000	-2.223969000
6	2.553608000	2.474215000	-1.412690000
1	2.081692000	2.719974000	-2.360381000
1	1.933582000	1.795745000	-0.831733000
6	3.974128000	2.004828000	-1.566445000
1	4.469175000	1.836629000	-0.615387000
1	4.572323000	2.644293000	-2.208355000
17	-3.354366000	-2.736014000	0.692073000
17	-0.332639000	-3.157988000	-2.548279000
17	2.604210000	4.078466000	-0.430047000
17	3.935678000	0.322892000	-2.406969000
6	-6.854584000	-2.203677000	-0.537120000
1	-6.119847000	-1.643927000	0.033427000
1	-7.620040000	-1.544472000	-0.935918000
6	-6.233518000	-3.089252000	-1.583763000
1	-5.461654000	-3.736258000	-1.177417000
1	-6.966507000	-3.654121000	-2.152184000
6	2.685634000	0.677534000	3.744409000
1	3.450951000	0.546139000	4.503990000
1	2.235682000	-0.272932000	3.470841000
6	3.173131000	1.459687000	2.554374000
1	2.423366000	1.549293000	1.773796000

Xanthione

1	3.577589000	2.431537000	2.821682000
17	-7.719036000	-3.315647000	0.702832000
17	-5.362023000	-1.987383000	-2.836234000
17	1.323391000	1.680418000	4.554555000
17	4.608624000	0.512745000	1.790281000
6	7.136612000	-0.964909000	-0.786848000
1	6.098292000	-0.827784000	-0.499100000
1	7.786622000	-0.977456000	0.083265000
6	7.580639000	0.014433000	-1.839774000
1	8.610350000	-0.135613000	-2.150978000
1	6.908818000	0.041804000	-2.692703000
6	-4.004728000	0.688741000	-0.732643000
1	-4.119496000	-0.389899000	-0.706529000
1	-3.339299000	1.032439000	0.051565000
6	-3.623769000	1.195715000	-2.095567000
1	-3.448124000	2.267518000	-2.116120000
1	-4.323962000	0.893138000	-2.868696000
17	-5.692744000	1.386977000	-0.299446000
17	-1.986266000	0.395336000	-2.554936000
17	7.238679000	-2.686238000	-1.530495000
17	7.518907000	1.734687000	-1.086806000

3. L_b

6	1.513684000	-1.131830000	0.699632000
6	1.780064000	-2.376313000	1.320572000
6	0.943234000	-2.749502000	2.404404000
6	-0.079064000	-1.929006000	2.844448000
6	-0.353108000	-0.682261000	2.243388000
6	0.494159000	-0.333601000	1.167860000
6	-1.407056000	0.192814000	2.637135000

Xanthione

6	-1.605520000	1.397482000	1.919675000
6	-0.732352000	1.731786000	0.837566000
6	-2.657600000	2.327440000	2.171898000
6	-2.830411000	3.459428000	1.407809000
6	-0.886372000	2.852133000	0.071284000
6	-1.949668000	3.765957000	0.331153000
1	-3.333276000	2.107197000	2.985770000
1	-0.194382000	3.013090000	-0.737310000
1	-3.654725000	4.116497000	1.640549000
1	2.080833000	-0.789332000	-0.152876000
1	-0.701445000	-2.237878000	3.673311000
1	1.100332000	-3.689570000	2.911982000
8	0.329697000	0.897058000	0.515035000
16	-2.504818000	-0.197408000	4.026874000
7	2.813826000	-3.183004000	0.902693000
7	-2.103891000	4.886915000	-0.434343000
6	3.766166000	-2.746285000	-0.122192000
1	3.909512000	-1.667671000	-0.029207000
1	4.729628000	-3.207213000	0.112551000
6	3.361488000	-3.105295000	-1.551285000
1	2.410286000	-2.640051000	-1.819933000
1	3.254802000	-4.187549000	-1.666845000
1	4.126097000	-2.763233000	-2.254295000
6	2.941009000	-4.557675000	1.398258000
1	3.938192000	-4.904252000	1.119088000
1	2.911378000	-4.560180000	2.493554000
6	1.887926000	-5.515737000	0.836508000
1	1.960845000	-5.576303000	-0.252239000
1	0.875930000	-5.187087000	1.088276000
1	2.030633000	-6.519171000	1.248686000
6	-1.165287000	5.204288000	-1.522133000
1	-1.246293000	6.276234000	-1.708900000

Xanthione

1	-0.144397000	5.024630000	-1.175426000
6	-1.434896000	4.433820000	-2.817748000
1	-2.436321000	4.647351000	-3.200164000
1	-1.343537000	3.354202000	-2.671352000
1	-0.709922000	4.731422000	-3.580828000
6	-3.258252000	5.779091000	-0.248929000
1	-3.401794000	5.961946000	0.819622000
1	-2.995591000	6.739098000	-0.695621000
6	-4.545035000	5.242291000	-0.880684000
1	-4.809075000	4.261478000	-0.477578000
1	-4.436902000	5.147177000	-1.964221000
1	-5.372257000	5.928995000	-0.679583000
6	-1.750231000	-2.649675000	-0.209848000
1	-1.032040000	-3.129082000	0.448474000
1	-1.517315000	-1.597549000	-0.336317000
6	-1.935072000	-3.361188000	-1.521651000
1	-2.192028000	-4.410120000	-1.402923000
1	-2.638148000	-2.855537000	-2.177641000
6	2.536638000	2.430078000	-1.532311000
1	2.075466000	2.621641000	-2.497600000
1	1.918111000	1.773062000	-0.925633000
6	3.964566000	1.971262000	-1.640879000
1	4.443113000	1.843030000	-0.675068000
1	4.568148000	2.593315000	-2.294707000
17	-3.390149000	-2.703060000	0.710327000
17	-0.286069000	-3.335461000	-2.414729000
17	2.551548000	4.083162000	-0.631778000
17	3.954615000	0.260978000	-2.419595000
6	-6.859498000	-2.191834000	-0.579318000
1	-6.129239000	-1.635068000	-0.000163000
1	-7.595379000	-1.525215000	-1.019254000
6	-6.227850000	-3.119783000	-1.582059000

Xanthione

1	-5.480201000	-3.768941000	-1.135977000
1	-6.956153000	-3.686904000	-2.154237000
6	2.671560000	0.761064000	3.722388000
1	3.426357000	0.650637000	4.495661000
1	2.236277000	-0.198641000	3.457663000
6	3.166664000	1.531236000	2.527610000
1	2.421516000	1.610676000	1.741583000
1	3.566500000	2.506890000	2.788060000
17	-7.790049000	-3.249777000	0.659871000
17	-5.300682000	-2.073671000	-2.842674000
17	1.284627000	1.761032000	4.494148000
17	4.609270000	0.581976000	1.780581000
6	7.151425000	-0.958225000	-0.740759000
1	6.111335000	-0.822031000	-0.459066000
1	7.800011000	-0.935881000	0.130212000
6	7.587693000	-0.009815000	-1.824777000
1	8.620205000	-0.158058000	-2.127427000
1	6.918672000	-0.019098000	-2.680248000
6	-3.984320000	0.699451000	-0.825903000
1	-4.078291000	-0.374905000	-0.703490000
1	-3.377894000	1.129312000	-0.036625000
6	-3.526244000	1.088072000	-2.204136000
1	-3.399165000	2.160215000	-2.323640000
1	-4.155633000	0.676383000	-2.987433000
17	-5.706302000	1.397954000	-0.566920000
17	-1.824950000	0.334594000	-2.469951000
17	7.271319000	-2.701970000	-1.427413000
17	7.503088000	1.734169000	-1.130796000

VI. OPTIMISED GEOMETRIES - DIMETHYL SULFOXIDE

A. Implicit

1. S_0

6	2.308116000	-0.785929000	-0.357273000
6	3.581965000	-0.205386000	-0.292901000
6	3.639071000	1.206044000	-0.111248000
6	2.502321000	1.952050000	-0.029919000
6	1.214897000	1.387390000	-0.105182000
6	1.175856000	-0.003207000	-0.271681000
6	0.000000000	2.152654000	-0.016748000
6	-1.214898000	1.387343000	-0.104950000
6	-1.175833000	-0.003253000	-0.271522000
6	-2.502317000	1.951949000	-0.029430000
6	-3.639070000	1.205904000	-0.110659000
6	-2.308068000	-0.786009000	-0.357008000
6	-3.581912000	-0.205466000	-0.292535000
1	-2.571481000	3.021987000	0.104386000
1	-2.166198000	-1.848061000	-0.470884000
1	-4.590449000	1.706351000	-0.034257000
1	2.166220000	-1.847982000	-0.471079000
1	2.571442000	3.022107000	0.103756000
1	4.590437000	1.706556000	-0.035185000
8	0.000029000	-0.676470000	-0.351681000
16	-0.000025000	3.832402000	0.176946000
7	4.712456000	-0.948352000	-0.411839000
7	-4.712406000	-0.948431000	-0.411447000
6	4.642312000	-2.396996000	-0.561719000
1	3.860136000	-2.640380000	-1.281404000
1	5.578799000	-2.717743000	-1.014281000
6	4.417000000	-3.153618000	0.741462000

Xanthione

1	3.505418000	-2.825833000	1.240582000
1	5.250373000	-3.007366000	1.427564000
1	4.327688000	-4.221795000	0.539729000
6	6.034477000	-0.346029000	-0.274160000
1	6.740493000	-1.017406000	-0.759327000
1	6.065367000	0.583981000	-0.841585000
6	6.465816000	-0.102442000	1.166362000
1	6.537161000	-1.039264000	1.717720000
1	5.761664000	0.545087000	1.688525000
1	7.445159000	0.377474000	1.184653000
6	-4.642358000	-2.396970000	-0.562265000
1	-5.578795000	-2.717325000	-1.015220000
1	-3.860067000	-2.639986000	-1.281954000
6	-4.417322000	-3.154404000	0.740482000
1	-5.250658000	-3.008290000	1.426660000
1	-3.505630000	-2.827144000	1.239750000
1	-4.328310000	-4.222501000	0.538172000
6	-6.034332000	-0.345878000	-0.274024000
1	-6.065137000	0.583883000	-0.841908000
1	-6.740453000	-1.017403000	-0.758848000
6	-6.465676000	-0.101726000	1.166391000
1	-5.761449000	0.545813000	1.688435000
1	-6.537206000	-1.038402000	1.717988000
1	-7.444950000	0.378343000	1.184491000

2. L_a

6	2.322712000	-0.779010000	-0.359803000
6	3.602498000	-0.195142000	-0.306347000
6	3.658215000	1.202359000	-0.140987000
6	2.507784000	1.955999000	-0.058500000

Xanthione

6	1.229142000	1.387329000	-0.120085000
6	1.189488000	-0.006978000	-0.274832000
6	-0.000009000	2.111563000	-0.026088000
6	-1.229152000	1.387311000	-0.120040000
6	-1.189481000	-0.006995000	-0.274787000
6	-2.507798000	1.955972000	-0.058408000
6	-3.658224000	1.202324000	-0.140841000
6	-2.322704000	-0.779037000	-0.359707000
6	-3.602495000	-0.195181000	-0.306187000
1	-2.601203000	3.028686000	0.061960000
1	-2.186285000	-1.843172000	-0.466602000
1	-4.606811000	1.710422000	-0.076901000
1	2.186285000	-1.843146000	-0.466692000
1	2.601199000	3.028712000	0.061870000
1	4.606795000	1.710475000	-0.077084000
8	0.000004000	-0.684126000	-0.339473000
16	-0.000006000	3.811560000	0.249928000
7	4.737602000	-0.955256000	-0.432476000
7	-4.737583000	-0.955318000	-0.432209000
6	4.659014000	-2.403826000	-0.535138000
1	3.876712000	-2.669638000	-1.247865000
1	5.594187000	-2.745262000	-0.976837000
6	4.428166000	-3.125711000	0.788124000
1	3.518344000	-2.778069000	1.277242000
1	5.261580000	-2.965559000	1.471358000
1	4.331796000	-4.198956000	0.616940000
6	6.055383000	-0.353815000	-0.300441000
1	6.764658000	-1.029201000	-0.776955000
1	6.086151000	0.570635000	-0.879087000
6	6.493265000	-0.086412000	1.135452000
1	6.578918000	-1.015524000	1.698079000
1	5.783276000	0.558340000	1.653132000

Xanthione

1	7.466903000	0.405965000	1.144138000
6	-4.658965000	-2.403878000	-0.535065000
1	-5.594228000	-2.745285000	-0.976588000
1	-3.876800000	-2.669604000	-1.247981000
6	-4.427873000	-3.125867000	0.788095000
1	-5.261118000	-2.965633000	1.471520000
1	-3.517905000	-2.778352000	1.277027000
1	-4.331664000	-4.199115000	0.616846000
6	-6.055382000	-0.353840000	-0.300577000
1	-6.085984000	0.570567000	-0.879304000
1	-6.764562000	-1.029250000	-0.777200000
6	-6.493589000	-0.086337000	1.135197000
1	-5.783705000	0.558432000	1.653001000
1	-6.579354000	-1.015430000	1.697839000
1	-7.467229000	0.406040000	1.143666000

3. L_b

6	2.323034000	-0.777434000	-0.372759000
6	3.601157000	-0.191516000	-0.297071000
6	3.647263000	1.208029000	-0.111781000
6	2.497050000	1.954537000	-0.031646000
6	1.214630000	1.388170000	-0.115499000
6	1.192709000	-0.003341000	-0.290169000
6	-0.008732000	2.132103000	-0.027174000
6	-1.215952000	1.385750000	-0.110768000
6	-1.177658000	-0.033247000	-0.292156000
6	-2.517637000	1.957588000	-0.023083000
6	-3.653453000	1.206328000	-0.109972000
6	-2.299214000	-0.795014000	-0.380701000
6	-3.593188000	-0.202488000	-0.297148000

Xanthione

1	-2.577435000	3.025616000	0.118924000
1	-2.170100000	-1.856770000	-0.511265000
1	-4.606547000	1.703670000	-0.032078000
1	2.187740000	-1.839853000	-0.495828000
1	2.563885000	3.024154000	0.108455000
1	4.594187000	1.716865000	-0.027915000
8	0.005748000	-0.693175000	-0.382516000
16	-0.066616000	3.858221000	0.184900000
7	4.737865000	-0.943959000	-0.415378000
7	-4.708555000	-0.955828000	-0.400420000
6	4.666729000	-2.391703000	-0.543559000
1	3.893948000	-2.647681000	-1.269805000
1	5.608971000	-2.721820000	-0.978080000
6	4.421810000	-3.133137000	0.766267000
1	3.503519000	-2.797291000	1.247514000
1	5.245177000	-2.979003000	1.462750000
1	4.333631000	-4.203921000	0.576991000
6	6.053772000	-0.339319000	-0.269248000
1	6.767339000	-1.006076000	-0.750942000
1	6.082893000	0.593110000	-0.834251000
6	6.480179000	-0.091823000	1.173562000
1	6.562780000	-1.028530000	1.723738000
1	5.765823000	0.545461000	1.694324000
1	7.453256000	0.400999000	1.194958000
6	-4.638287000	-2.406563000	-0.566077000
1	-5.579003000	-2.719355000	-1.012631000
1	-3.859394000	-2.641494000	-1.289546000
6	-4.405856000	-3.163834000	0.736501000
1	-5.230932000	-3.014606000	1.430882000
1	-3.485175000	-2.844731000	1.223555000
1	-4.328776000	-4.230382000	0.524080000
6	-6.037936000	-0.364661000	-0.257890000

Xanthione

1	-6.072677000	0.564005000	-0.825834000
1	-6.739121000	-1.045799000	-0.733754000
6	-6.451334000	-0.122959000	1.189580000
1	-5.746127000	0.530664000	1.701381000
1	-6.514820000	-1.058741000	1.742344000
1	-7.432853000	0.351092000	1.209090000

B. S=2

1. S_0

c6	-3.280660000	-0.467021000	0.008526000
6	-4.231671000	-1.502862000	-0.073826000
6	-3.741869000	-2.838148000	-0.224108000
6	-2.396580000	-3.094483000	-0.309185000
6	-1.423370000	-2.068725000	-0.251559000
6	-1.938701000	-0.765564000	-0.090829000
6	-0.006296000	-2.294035000	-0.344545000
6	0.833845000	-1.128899000	-0.284231000
6	0.275830000	0.155506000	-0.131032000
6	2.247147000	-1.162388000	-0.375817000
6	3.025019000	-0.035035000	-0.317030000
6	1.022688000	1.315669000	-0.069858000
6	2.426813000	1.257054000	-0.167592000
1	2.711934000	-2.131392000	-0.505558000
1	0.488370000	2.242198000	0.069376000
1	4.101491000	-0.138990000	-0.411188000
1	-3.554056000	0.575003000	0.143188000
1	-2.047377000	-4.111998000	-0.425718000
1	-4.430821000	-3.668388000	-0.270746000
8	-1.090001000	0.327119000	-0.029719000

Xanthione

16	0.660640000	-3.897971000	-0.524724000
7	-5.571527000	-1.230913000	-0.025231000
7	3.189489000	2.391538000	-0.137079000
6	-6.048713000	0.147163000	0.171570000
1	-5.435705000	0.832632000	-0.418814000
1	-7.063598000	0.195252000	-0.230124000
6	-6.041093000	0.595629000	1.633555000
1	-5.037976000	0.512556000	2.058820000
1	-6.725428000	-0.006479000	2.237362000
1	-6.347179000	1.643226000	1.703288000
6	-6.571324000	-2.305707000	-0.057767000
1	-7.514787000	-1.855019000	-0.372781000
1	-6.300470000	-3.025679000	-0.835834000
6	-6.760579000	-3.017700000	1.283149000
1	-7.121278000	-2.322968000	2.045832000
1	-5.821641000	-3.452105000	1.637330000
1	-7.492682000	-3.824462000	1.181864000
6	2.561811000	3.713193000	-0.017287000
1	3.275641000	4.444065000	-0.403276000
1	1.691516000	3.751368000	-0.680172000
6	2.158147000	4.089989000	1.410244000
1	3.035106000	4.160441000	2.058398000
1	1.477442000	3.350713000	1.840719000
1	1.653094000	5.060580000	1.413570000
6	4.658996000	2.323896000	-0.087706000
1	5.020778000	1.549448000	-0.765892000
1	5.035207000	3.280418000	-0.458012000
6	5.204253000	2.048211000	1.313170000
1	4.779555000	1.123894000	1.713642000
1	4.968146000	2.862709000	2.003123000
1	6.290470000	1.929618000	1.273660000
16	6.847101000	-1.719910000	0.065428000

Xanthione

8	6.147568000	-0.422130000	-0.769194000
6	6.015351000	-3.230615000	-0.640986000
6	6.029495000	-1.709278000	1.740739000
1	6.326672000	-3.306493000	-1.680373000
1	6.387932000	-0.825231000	2.262842000
1	4.936369000	-3.101112000	-0.571548000
1	6.347393000	-4.099418000	-0.074944000
1	6.324304000	-2.615060000	2.268389000
1	4.950293000	-1.661698000	1.600986000
16	-2.370539000	3.324714000	-0.297269000
8	-3.854184000	2.630270000	0.149879000
6	-2.078568000	2.708726000	-2.029706000
6	-2.819471000	5.086769000	-0.700629000
1	-1.843082000	1.651454000	-1.944026000
1	-3.097410000	5.566658000	0.235131000
1	-1.239673000	3.257686000	-2.454933000
1	-2.991002000	2.863707000	-2.603833000
1	-3.660373000	5.076156000	-1.392701000
1	-1.948214000	5.572862000	-1.137128000

2. L_a

6	3.290947000	-0.472291000	0.004315000
6	4.246351000	-1.510571000	0.100294000
6	3.761742000	-2.829939000	0.270660000
6	2.402490000	-3.087607000	0.361056000
6	1.437013000	-2.064529000	0.282812000
6	1.950731000	-0.761709000	0.101449000
6	0.021786000	-2.249922000	0.358654000
6	-0.846084000	-1.115334000	0.311811000
6	-0.289360000	0.168328000	0.137425000

Xanthione

6	-2.252127000	-1.155382000	0.418148000
6	-3.039601000	-0.018314000	0.352489000
6	-1.043011000	1.319618000	0.072648000
6	-2.450776000	1.260047000	0.183019000
1	-2.735082000	-2.115280000	0.574432000
1	-0.515716000	2.248611000	-0.081808000
1	-4.114182000	-0.125080000	0.460134000
1	3.568893000	0.566644000	-0.142319000
1	2.075158000	-4.112004000	0.504492000
1	4.447081000	-3.662263000	0.338598000
8	1.089268000	0.336977000	0.014444000
16	-0.663626000	-3.896132000	0.408941000
7	5.597141000	-1.225925000	0.050814000
7	-3.216130000	2.409548000	0.149409000
6	6.064709000	0.148513000	-0.165546000
1	5.451017000	0.840199000	0.417668000
1	7.081139000	0.209164000	0.232807000
6	6.053697000	0.585122000	-1.632173000
1	5.050404000	0.490861000	-2.055192000
1	6.740496000	-0.019698000	-2.230971000
1	6.353379000	1.634055000	-1.714054000
6	6.592580000	-2.299028000	0.073289000
1	7.544875000	-1.849470000	0.364837000
1	6.335647000	-3.012656000	0.863658000
6	6.756849000	-3.030923000	-1.261676000
1	7.111480000	-2.348186000	-2.038230000
1	5.808022000	-3.459946000	-1.595497000
1	7.482965000	-3.843794000	-1.162878000
6	-2.585548000	3.726343000	0.042573000
1	-3.293177000	4.457390000	0.442344000
1	-1.710726000	3.754071000	0.700876000
6	-2.184778000	4.127661000	-1.380331000

Xanthione

1	-3.064374000	4.213074000	-2.023650000
1	-1.510521000	3.391020000	-1.825769000
1	-1.674292000	5.095789000	-1.371115000
6	-4.678349000	2.333574000	0.047387000
1	-5.063541000	1.580295000	0.738279000
1	-5.073701000	3.300139000	0.370419000
6	-5.177054000	2.005940000	-1.361060000
1	-4.724225000	1.077095000	-1.717790000
1	-4.930148000	2.802515000	-2.068279000
1	-6.262042000	1.869305000	-1.353469000
16	-6.833428000	-1.737619000	-0.119498000
8	-6.213587000	-0.416375000	0.741299000
6	-6.111948000	-3.226968000	0.737198000
6	-5.835992000	-1.804669000	-1.694007000
1	-6.554063000	-3.264449000	1.730342000
1	-6.133140000	-0.946912000	-2.292951000
1	-5.032041000	-3.102939000	0.803182000
1	-6.375795000	-4.114834000	0.164774000
1	-6.075529000	-2.734281000	-2.208194000
1	-4.777946000	-1.746542000	-1.442243000
16	2.362736000	3.329724000	0.271163000
8	3.857095000	2.651484000	-0.164363000
6	2.049984000	2.693571000	1.992477000
6	2.796353000	5.089780000	0.701155000
1	1.813375000	1.638417000	1.887109000
1	3.086100000	5.581598000	-0.224809000
1	1.206804000	3.238860000	2.414142000
1	2.956045000	2.840741000	2.578693000
1	3.626917000	5.075694000	1.405616000
1	1.916642000	5.567336000	1.130108000

Xanthione

3. L_b

6	-3.251153000	-0.474371000	0.011378000
6	-4.201131000	-1.534547000	-0.059514000
6	-3.698594000	-2.863079000	-0.208389000
6	-2.348195000	-3.102320000	-0.295688000
6	-1.376570000	-2.058119000	-0.248677000
6	-1.916277000	-0.750271000	-0.087986000
6	0.038293000	-2.270725000	-0.352564000
6	0.864954000	-1.099662000	-0.284197000
6	0.300226000	0.195995000	-0.128972000
6	2.284158000	-1.121214000	-0.367906000
6	3.058104000	0.013826000	-0.300405000
6	1.038263000	1.350059000	-0.060262000
6	2.454891000	1.298280000	-0.143440000
1	2.748669000	-2.089702000	-0.500758000
1	0.502624000	2.276892000	0.078603000
1	4.136391000	-0.082876000	-0.391418000
1	-3.545436000	0.566061000	0.129500000
1	-1.978697000	-4.112426000	-0.407911000
1	-4.377027000	-3.702656000	-0.251007000
8	-1.079186000	0.365855000	-0.036562000
16	0.725619000	-3.907486000	-0.560475000
7	-5.537396000	-1.264819000	0.003550000
7	3.210454000	2.438800000	-0.084331000
6	-6.028268000	0.108881000	0.214680000
1	-5.427271000	0.808170000	-0.371108000
1	-7.048082000	0.144196000	-0.172907000
6	-6.008007000	0.536485000	1.684646000
1	-4.994110000	0.486254000	2.088454000
1	-6.660243000	-0.094044000	2.293787000
1	-6.351010000	1.571308000	1.766094000

Xanthione

6	-6.533953000	-2.344009000	-0.041554000
1	-7.478550000	-1.895106000	-0.352454000
1	-6.250826000	-3.055017000	-0.822450000
6	-6.715338000	-3.065184000	1.297560000
1	-7.092386000	-2.382647000	2.062529000
1	-5.771390000	-3.487802000	1.650955000
1	-7.434701000	-3.880848000	1.182150000
6	2.579888000	3.761645000	-0.001931000
1	3.296293000	4.484613000	-0.397659000
1	1.714592000	3.780796000	-0.670380000
6	2.166438000	4.167078000	1.416345000
1	3.038443000	4.253760000	2.068884000
1	1.483177000	3.435445000	1.855469000
1	1.660973000	5.136947000	1.392244000
6	4.675843000	2.370614000	0.032544000
1	5.071745000	1.625632000	-0.660513000
1	5.066295000	3.344097000	-0.270387000
6	5.149227000	2.025418000	1.446186000
1	4.706158000	1.083035000	1.777608000
1	4.880233000	2.806335000	2.161882000
1	6.235586000	1.905351000	1.449496000
16	6.819687000	-1.698313000	0.113617000
8	6.188361000	-0.325968000	-0.654821000
6	6.153026000	-3.130989000	-0.874639000
6	5.786481000	-1.904732000	1.651891000
1	6.625084000	-3.086162000	-1.853629000
1	6.028045000	-1.071290000	2.307591000
1	5.073214000	-3.022307000	-0.964083000
1	6.418172000	-4.053980000	-0.361574000
1	6.056653000	-2.851089000	2.117322000
1	4.733191000	-1.882432000	1.375669000
16	-2.484005000	3.313242000	-0.343460000

Xanthione

8	-3.946829000	2.537025000	0.036167000
6	-2.067612000	2.699689000	-2.051135000
6	-3.023748000	5.039203000	-0.786827000
1	-1.760032000	1.663991000	-1.935115000
1	-3.367295000	5.511072000	0.130998000
1	-1.251221000	3.303766000	-2.444724000
1	-2.960918000	2.781561000	-2.668792000
1	-3.834668000	4.967427000	-1.510198000
1	-2.168328000	5.573717000	-1.196740000

C. S=4

1. S_0

6	-1.441200000	-0.091921000	0.222479000
6	-2.705428000	-0.590157000	0.600850000
6	-2.854198000	-2.011639000	0.686479000
6	-1.795511000	-2.843539000	0.431388000
6	-0.511800000	-2.364207000	0.074950000
6	-0.395419000	-0.962120000	-0.018000000
6	0.610719000	-3.217061000	-0.197078000
6	1.845229000	-2.562530000	-0.535967000
6	1.932063000	-1.155659000	-0.590118000
6	3.048381000	-3.252339000	-0.810243000
6	4.218898000	-2.601650000	-1.100956000
6	3.097680000	-0.461232000	-0.851430000
6	4.285339000	-1.172974000	-1.131496000
1	3.024292000	-4.333503000	-0.781141000
1	3.068839000	0.623305000	-0.794329000
1	5.100549000	-3.191181000	-1.300476000
1	-1.257172000	0.964638000	0.119989000

Xanthione

1	-1.926259000	-3.916032000	0.497504000
1	-3.821536000	-2.447711000	0.901493000
8	0.809266000	-0.374550000	-0.360168000
16	0.485390000	-4.956101000	-0.114963000
7	-3.751443000	0.245967000	0.869261000
7	5.458320000	-0.533703000	-1.423978000
6	-3.581604000	1.704708000	0.801489000
1	-3.018995000	1.978675000	-0.094701000
1	-4.578784000	2.134572000	0.678796000
6	-2.907988000	2.305797000	2.037893000
1	-1.946352000	1.824199000	2.237338000
1	-3.533240000	2.195101000	2.927274000
1	-2.721734000	3.371923000	1.879343000
6	-5.014361000	-0.267501000	1.429685000
1	-5.769002000	0.503564000	1.258304000
1	-5.335287000	-1.156130000	0.878501000
6	-4.930153000	-0.592572000	2.922655000
1	-4.698467000	0.296257000	3.515116000
1	-4.157278000	-1.342184000	3.115942000
1	-5.886133000	-0.995646000	3.270878000
6	5.559694000	0.932964000	-1.441761000
1	6.254606000	1.199911000	-2.243828000
1	4.592951000	1.366854000	-1.693446000
6	6.034926000	1.525574000	-0.115341000
1	7.070622000	1.250839000	0.103071000
1	5.402583000	1.178970000	0.705480000
1	5.962517000	2.615327000	-0.151402000
6	6.707284000	-1.285183000	-1.613992000
1	6.531248000	-2.114076000	-2.306706000
1	7.406584000	-0.611694000	-2.113781000
6	7.329580000	-1.800200000	-0.313894000
1	6.642028000	-2.455621000	0.227151000

Xanthione

1	7.594775000	-0.971393000	0.346417000
1	8.238523000	-2.368350000	-0.532971000
16	2.018032000	0.044267000	2.760072000
8	1.554030000	-0.016678000	4.383965000
6	3.319348000	-1.281292000	2.603451000
6	3.124011000	1.542312000	2.620660000
1	2.807125000	-2.240893000	2.617830000
1	2.597752000	2.343878000	3.136928000
1	3.992070000	-1.187669000	3.455119000
1	3.841854000	-1.142995000	1.659345000
1	3.247202000	1.793677000	1.562592000
1	4.072541000	1.336646000	3.115603000
16	-2.497141000	5.133658000	-1.551429000
8	-1.847336000	3.613649000	-1.154366000
6	-4.133753000	5.169313000	-0.663002000
6	-1.544591000	6.315357000	-0.471831000
1	-4.761232000	4.412826000	-1.128915000
1	-0.515008000	6.300359000	-0.823172000
1	-4.567757000	6.159851000	-0.788162000
1	-3.963701000	4.937625000	0.387181000
1	-1.605522000	5.967120000	0.558288000
1	-1.975922000	7.308599000	-0.584696000
16	-5.877926000	-3.258072000	-1.498279000
8	-5.848985000	-3.165277000	0.196827000
6	-4.389078000	-4.287194000	-1.946450000
6	-5.265580000	-1.598430000	-2.095430000
1	-4.560131000	-5.281541000	-1.539952000
1	-6.014471000	-0.862990000	-1.809761000
1	-3.504541000	-3.835073000	-1.501638000
1	-4.317093000	-4.323078000	-3.032340000
1	-5.169228000	-1.647422000	-3.178965000
1	-4.311017000	-1.381654000	-1.619521000

Xanthione

16	1.665923000	3.462774000	-0.762966000
8	3.136071000	2.658093000	-0.441978000
6	0.837038000	2.454901000	-2.092598000
6	0.557222000	3.028220000	0.666804000
1	1.394659000	2.621055000	-3.011882000
1	0.878580000	3.622662000	1.519453000
1	-0.188545000	2.828024000	-2.151682000
1	0.866432000	1.409706000	-1.791288000
1	0.671002000	1.963823000	0.865243000
1	-0.456367000	3.286222000	0.349224000

2. L_a

6	-1.371519000	-0.180458000	0.337085000
6	-2.582265000	-0.855953000	0.616344000
6	-2.594283000	-2.263757000	0.458167000
6	-1.453655000	-2.943290000	0.064646000
6	-0.232493000	-2.287994000	-0.193572000
6	-0.251174000	-0.885403000	-0.043450000
6	0.984327000	-2.932593000	-0.568443000
6	2.158917000	-2.154404000	-0.799460000
6	2.104389000	-0.752603000	-0.634476000
6	3.418128000	-2.673417000	-1.157588000
6	4.530669000	-1.865134000	-1.320893000
6	3.189413000	0.081199000	-0.792641000
6	4.451093000	-0.462117000	-1.144208000
1	3.526975000	-3.742782000	-1.303168000
1	3.040090000	1.149622000	-0.657384000
1	5.468239000	-2.332329000	-1.582812000
1	-1.292267000	0.891046000	0.423272000
1	-1.507544000	-4.020902000	-0.052934000

Xanthione

1	-3.513304000	-2.821209000	0.585682000
8	0.903606000	-0.130174000	-0.269759000
16	1.063236000	-4.713036000	-0.649475000
7	-3.710682000	-0.164218000	1.010659000
7	5.551694000	0.352596000	-1.313582000
6	-3.684874000	1.296779000	1.136185000
1	-3.169914000	1.738252000	0.278186000
1	-4.721804000	1.639554000	1.075189000
6	-3.053729000	1.807291000	2.435941000
1	-2.042000000	1.411921000	2.566011000
1	-3.645323000	1.515761000	3.307733000
1	-2.986477000	2.899777000	2.419403000
6	-4.871376000	-0.880454000	1.560565000
1	-5.703063000	-0.171241000	1.568014000
1	-5.158414000	-1.696791000	0.889797000
6	-4.644558000	-1.429249000	2.972047000
1	-4.446428000	-0.625847000	3.686567000
1	-3.794764000	-2.117826000	2.989878000
1	-5.530168000	-1.976858000	3.309762000
6	5.476469000	1.788467000	-1.014951000
1	6.286889000	2.273547000	-1.565905000
1	4.538250000	2.198791000	-1.395212000
6	5.590682000	2.117006000	0.475640000
1	6.574345000	1.850093000	0.871872000
1	4.832215000	1.577219000	1.049329000
1	5.427939000	3.186348000	0.634482000
6	6.869195000	-0.219935000	-1.600002000
1	6.771398000	-0.969341000	-2.392381000
1	7.484768000	0.582622000	-2.013190000
6	7.569333000	-0.829575000	-0.381619000
1	6.952422000	-1.603615000	0.083294000
1	7.776543000	-0.065095000	0.371410000

Xanthione

1	8.519997000	-1.283482000	-0.677956000
16	1.992909000	-1.352993000	2.687191000
8	1.662974000	-1.827845000	4.272474000
6	3.781964000	-1.796604000	2.414485000
6	2.189665000	0.496829000	2.783497000
1	3.837879000	-2.882371000	2.377966000
1	1.203321000	0.909423000	2.984881000
1	4.363660000	-1.408258000	3.249686000
1	4.102081000	-1.367612000	1.466736000
1	2.566680000	0.853092000	1.826842000
1	2.876174000	0.727782000	3.597093000
16	-3.077623000	5.001630000	-0.977495000
8	-2.261166000	3.521158000	-0.801386000
6	-4.599496000	4.811599000	0.079248000
6	-2.112578000	6.167908000	0.108244000
1	-5.223141000	4.057943000	-0.396254000
1	-1.129572000	6.278047000	-0.344823000
1	-5.111967000	5.771570000	0.112715000
1	-4.292599000	4.488402000	1.072782000
1	-2.032776000	5.727691000	1.101301000
1	-2.634405000	7.122958000	0.135494000
16	-5.590188000	-3.121514000	-1.957886000
8	-5.557216000	-3.518478000	-0.307552000
6	-3.973305000	-3.751615000	-2.640090000
6	-5.237798000	-1.289793000	-2.040932000
1	-3.997905000	-4.836065000	-2.557347000
1	-6.098395000	-0.782596000	-1.610210000
1	-3.158982000	-3.337339000	-2.048404000
1	-3.906900000	-3.447730000	-3.683565000
1	-5.119092000	-1.020786000	-3.089540000
1	-4.339249000	-1.073251000	-1.465990000
16	1.182363000	3.771712000	-0.822312000

Xanthione

8	2.801107000	3.264143000	-0.700669000
6	0.389915000	2.620205000	-2.053562000
6	0.379148000	3.134544000	0.728537000
1	0.815439000	2.854434000	-3.027221000
1	0.766953000	3.722692000	1.557875000
1	-0.680483000	2.831779000	-2.006458000
1	0.615594000	1.598220000	-1.753954000
1	0.650457000	2.085701000	0.827219000
1	-0.695264000	3.272328000	0.590983000

3. *L_b*

6	-1.388305000	0.050463000	0.250619000
6	-2.661396000	-0.520785000	0.556526000
6	-2.784411000	-1.943290000	0.480465000
6	-1.703085000	-2.722844000	0.148073000
6	-0.416973000	-2.178775000	-0.140486000
6	-0.328599000	-0.755069000	-0.063933000
6	0.724079000	-2.972904000	-0.491672000
6	1.950879000	-2.269807000	-0.723657000
6	2.024860000	-0.858314000	-0.615454000
6	3.177193000	-2.903927000	-1.044330000
6	4.351386000	-2.206676000	-1.218761000
6	3.176618000	-0.125350000	-0.776895000
6	4.394416000	-0.789039000	-1.088895000
1	3.168756000	-3.981517000	-1.137863000
1	3.120131000	0.956470000	-0.675705000
1	5.249141000	-2.760607000	-1.450102000
1	-1.235365000	1.117664000	0.265778000
1	-1.799849000	-3.798840000	0.099397000
1	-3.745373000	-2.421429000	0.634655000

Xanthione

8	0.878402000	-0.107957000	-0.311768000
16	0.592531000	-4.749916000	-0.614597000
7	-3.711102000	0.267794000	0.920568000
7	5.560167000	-0.086821000	-1.262000000
6	-3.568882000	1.728990000	1.020667000
1	-3.024371000	2.120696000	0.156499000
1	-4.576415000	2.146342000	0.970732000
6	-2.884251000	2.188796000	2.313131000
1	-1.899327000	1.727824000	2.426989000
1	-3.481782000	1.941881000	3.193649000
1	-2.746797000	3.273446000	2.281956000
6	-4.988163000	-0.322216000	1.359266000
1	-5.746952000	0.455006000	1.254240000
1	-5.265111000	-1.148766000	0.696499000
6	-4.945944000	-0.822400000	2.807367000
1	-4.756064000	-0.006836000	3.509331000
1	-4.166221000	-1.577841000	2.936144000
1	-5.907131000	-1.277983000	3.062268000
6	5.611501000	1.366076000	-1.043515000
1	6.448027000	1.749603000	-1.633024000
1	4.701472000	1.832072000	-1.426523000
6	5.784735000	1.754968000	0.426969000
1	6.755867000	1.438401000	0.816634000
1	5.002318000	1.300680000	1.040616000
1	5.701048000	2.839656000	0.530275000
6	6.827773000	-0.780823000	-1.517455000
1	6.671061000	-1.542928000	-2.286811000
1	7.513621000	-0.046327000	-1.944162000
6	7.454498000	-1.408142000	-0.268228000
1	6.770007000	-2.116133000	0.206566000
1	7.712902000	-0.641329000	0.465832000
1	8.368597000	-1.945201000	-0.538096000

Xanthione

16	1.758610000	-1.257514000	2.725610000
8	1.306622000	-1.651235000	4.302444000
6	3.429544000	-2.048743000	2.490163000
6	2.321384000	0.515619000	2.824076000
1	3.265567000	-3.118989000	2.385637000
1	1.443580000	1.113088000	3.060797000
1	4.032953000	-1.832278000	3.370851000
1	3.879070000	-1.643834000	1.585320000
1	2.730284000	0.800660000	1.856407000
1	3.066590000	0.600781000	3.614018000
16	-2.581557000	5.254051000	-1.140135000
8	-1.947919000	3.710328000	-0.810666000
6	-4.195478000	5.286175000	-0.210666000
6	-1.581620000	6.376938000	-0.040811000
1	-4.844512000	4.550689000	-0.680669000
1	-0.562634000	6.363412000	-0.422012000
1	-4.619071000	6.285177000	-0.299504000
1	-4.002744000	5.026323000	0.829021000
1	-1.619863000	5.984748000	0.974637000
1	-2.001286000	7.379827000	-0.099203000
16	-5.731163000	-3.329049000	-1.710438000
8	-5.686971000	-3.105288000	-0.026832000
6	-4.278323000	-4.438053000	-2.078630000
6	-5.067176000	-1.737789000	-2.423644000
1	-4.475034000	-5.387479000	-1.585586000
1	-5.781903000	-0.957050000	-2.172521000
1	-3.374014000	-3.978072000	-1.683973000
1	-4.218805000	-4.571004000	-3.157666000
1	-4.991826000	-1.855933000	-3.503455000
1	-4.096229000	-1.531681000	-1.976070000
16	1.550151000	3.723968000	-0.857567000
8	3.107720000	3.051866000	-0.734327000

Xanthione

6	0.650874000	2.666703000	-2.099989000
6	0.684374000	3.162354000	0.688947000
1	1.074096000	2.893181000	-3.076366000
1	1.130394000	3.702927000	1.521279000
1	-0.401044000	2.952263000	-2.025759000
1	0.805322000	1.623805000	-1.829263000
1	0.847261000	2.090615000	0.781611000
1	-0.370857000	3.408823000	0.554427000

D. S=6

1. S_0

6	-3.234753000	-0.212265000	-1.485240000
6	-4.646621000	-0.159947000	-1.481331000
6	-5.259715000	1.086235000	-1.136724000
6	-4.507710000	2.175313000	-0.789744000
6	-3.092224000	2.142118000	-0.759533000
6	-2.512398000	0.905751000	-1.123999000
6	-2.276804000	3.259596000	-0.382035000
6	-0.854339000	3.050191000	-0.393505000
6	-0.314915000	1.798945000	-0.755192000
6	0.095227000	4.042754000	-0.051716000
6	1.445258000	3.805721000	-0.060178000
6	1.038160000	1.523964000	-0.767744000
6	1.968907000	2.520072000	-0.406509000
1	-0.279663000	5.019463000	0.224542000
1	1.345236000	0.537586000	-1.077614000
1	2.116937000	4.607891000	0.206289000
1	-2.687223000	-1.120344000	-1.714748000
1	-4.991341000	3.105476000	-0.522421000

Xanthione

1	-6.334586000	1.163657000	-1.118674000
8	-1.135263000	0.751810000	-1.124437000
16	-2.973915000	4.795560000	0.081589000
7	-5.402180000	-1.252659000	-1.786322000
7	3.307553000	2.261749000	-0.369568000
6	-4.767087000	-2.510436000	-2.207555000
1	-3.881526000	-2.702179000	-1.598100000
1	-5.481027000	-3.312369000	-2.005061000
6	-4.369069000	-2.523521000	-3.683818000
1	-3.697104000	-1.690814000	-3.910168000
1	-5.243408000	-2.447307000	-4.336547000
1	-3.839914000	-3.451834000	-3.916622000
6	-6.872884000	-1.192272000	-1.775213000
1	-7.226781000	-2.221502000	-1.678896000
1	-7.198204000	-0.671195000	-0.869230000
6	-7.472698000	-0.564113000	-3.034814000
1	-7.217416000	-1.145149000	-3.925266000
1	-7.109519000	0.456897000	-3.183004000
1	-8.563713000	-0.527057000	-2.953244000
6	3.825127000	0.923021000	-0.686879000
1	4.810377000	0.849713000	-0.222161000
1	3.181454000	0.179903000	-0.202789000
6	3.943915000	0.629581000	-2.182909000
1	4.651952000	1.318893000	-2.652566000
1	2.982240000	0.734622000	-2.693968000
1	4.311186000	-0.393689000	-2.319038000
6	4.293844000	3.317158000	-0.087185000
1	3.965854000	3.895965000	0.782320000
1	5.224629000	2.815160000	0.188885000
6	4.548965000	4.240694000	-1.280139000
1	3.630950000	4.730710000	-1.617287000
1	4.960189000	3.676223000	-2.121715000

Xanthione

1	5.269342000	5.017999000	-1.006953000
16	7.787002000	-0.854160000	3.144771000
8	7.938761000	-1.887864000	1.800976000
6	6.122784000	-0.032990000	2.945255000
6	8.848645000	0.620764000	2.724418000
1	5.364086000	-0.799434000	3.085956000
1	9.852758000	0.242447000	2.542759000
1	6.100701000	0.396785000	1.941256000
1	6.038787000	0.732303000	3.716191000
1	8.840166000	1.291676000	3.582667000
1	8.413723000	1.084807000	1.835235000
16	-0.648772000	-3.497999000	-0.553790000
8	-1.723541000	-3.074997000	-1.799201000
6	-0.664186000	-2.042708000	0.610608000
6	-1.599132000	-4.698301000	0.512868000
1	-0.367678000	-1.170599000	0.032917000
1	-1.901405000	-5.519157000	-0.134461000
1	0.043798000	-2.249368000	1.412423000
1	-1.687473000	-1.949758000	0.980594000
1	-2.455982000	-4.167963000	0.939233000
1	-0.921927000	-5.053612000	1.289327000
16	-8.105829000	0.897986000	2.163599000
8	-7.246582000	-0.256095000	1.253562000
6	-9.797365000	0.879167000	1.383849000
6	-7.501789000	2.536503000	1.514295000
1	-10.251295000	-0.076026000	1.637511000
1	-6.446690000	2.608303000	1.768114000
1	-9.672168000	0.974125000	0.306071000
1	-10.377487000	1.703795000	1.794695000
1	-8.071100000	3.325171000	2.003126000
1	-7.639615000	2.556710000	0.434591000
16	4.779737000	-3.470149000	-0.828890000

Xanthione

8	5.306425000	-2.400221000	-2.039835000
6	4.967678000	-2.533724000	0.768511000
6	2.922412000	-3.419730000	-0.970448000
1	6.034138000	-2.423400000	0.990152000
1	2.658400000	-3.878656000	-1.920351000
1	4.468464000	-3.109384000	1.548124000
1	4.501761000	-1.557151000	0.642595000
1	2.601652000	-2.379102000	-0.945341000
1	2.500299000	-3.984977000	-0.141962000
16	-3.962897000	-1.662520000	2.908614000
8	-3.717552000	-2.605845000	1.512781000
6	-4.198382000	0.068093000	2.256149000
6	-5.725993000	-2.023954000	3.400865000
1	-3.294330000	0.325977000	1.708909000
1	-5.793384000	-3.101669000	3.534976000
1	-4.332137000	0.729542000	3.111916000
1	-5.079844000	0.069467000	1.612523000
1	-6.383402000	-1.655785000	2.607842000
1	-5.917724000	-1.508276000	4.341618000
16	7.599484000	1.011979000	-1.239485000
8	6.962807000	1.399467000	0.299846000
6	9.358104000	1.613952000	-1.123083000
6	7.894504000	-0.828199000	-1.182482000
1	9.312137000	2.683595000	-0.931116000
1	6.972095000	-1.321803000	-1.507125000
1	9.848841000	1.413357000	-2.074258000
1	9.853716000	1.093837000	-0.305352000
1	8.126784000	-1.093992000	-0.144921000
1	8.714632000	-1.047939000	-1.866317000

Xanthione

2. *L_a*

6	-3.252335000	-0.154289000	-1.501506000
6	-4.669391000	-0.106496000	-1.479439000
6	-5.275133000	1.112928000	-1.088337000
6	-4.513929000	2.205397000	-0.716280000
6	-3.104124000	2.174298000	-0.720013000
6	-2.523206000	0.950983000	-1.126871000
6	-2.257943000	3.267417000	-0.362249000
6	-0.841656000	3.084650000	-0.329027000
6	-0.301289000	1.843513000	-0.730942000
6	0.097876000	4.058592000	0.066458000
6	1.460719000	3.814973000	0.067899000
6	1.048501000	1.572609000	-0.731726000
6	1.980955000	2.556053000	-0.321917000
1	-0.258358000	5.030515000	0.391138000
1	1.361722000	0.597137000	-1.071000000
1	2.126169000	4.603977000	0.386150000
1	-2.709888000	-1.056768000	-1.762337000
1	-5.018319000	3.112250000	-0.399120000
1	-6.349770000	1.186829000	-1.037114000
8	-1.130781000	0.809220000	-1.163713000
16	-2.960102000	4.885840000	-0.094707000
7	-5.424286000	-1.211407000	-1.802694000
7	3.329435000	2.280501000	-0.279238000
6	-4.785132000	-2.450329000	-2.259526000
1	-3.892315000	-2.652877000	-1.663484000
1	-5.490528000	-3.263931000	-2.069536000
6	-4.399786000	-2.432928000	-3.740330000
1	-3.733320000	-1.592584000	-3.955375000
1	-5.281116000	-2.344672000	-4.382577000
1	-3.870403000	-3.354511000	-3.999791000

Xanthione

6	-6.890397000	-1.150055000	-1.790755000
1	-7.248686000	-2.181524000	-1.737322000
1	-7.218573000	-0.665513000	-0.864828000
6	-7.494974000	-0.468116000	-3.021417000
1	-7.247054000	-1.015529000	-3.935191000
1	-7.124186000	0.555055000	-3.130465000
1	-8.585634000	-0.426158000	-2.933741000
6	3.839274000	0.955808000	-0.646851000
1	4.821244000	0.855351000	-0.179275000
1	3.189590000	0.194278000	-0.198882000
6	3.967828000	0.717564000	-2.153053000
1	4.685513000	1.418648000	-2.590052000
1	3.010560000	0.853839000	-2.665295000
1	4.325300000	-0.303044000	-2.329583000
6	4.311854000	3.322843000	0.039482000
1	3.983810000	3.870684000	0.930438000
1	5.244610000	2.814598000	0.297917000
6	4.567563000	4.296199000	-1.114629000
1	3.646275000	4.789939000	-1.436984000
1	4.987796000	3.768065000	-1.975379000
1	5.279458000	5.069275000	-0.808059000
16	7.801539000	-1.009925000	3.118303000
8	7.938980000	-1.986932000	1.731238000
6	6.150610000	-0.154764000	2.953588000
6	8.887295000	0.464181000	2.761819000
1	5.379859000	-0.914779000	3.059467000
1	9.884213000	0.077084000	2.560037000
1	6.136916000	0.319897000	1.969609000
1	6.077382000	0.576451000	3.758017000
1	8.892636000	1.095506000	3.649555000
1	8.458250000	0.975136000	1.895698000
16	-0.638578000	-3.441996000	-0.632148000

Xanthione

8	-1.729455000	-3.041025000	-1.870610000
6	-0.679727000	-1.990332000	0.536108000
6	-1.555564000	-4.667195000	0.435518000
1	-0.421103000	-1.107470000	-0.044279000
1	-1.843483000	-5.491965000	-0.213444000
1	0.046299000	-2.177491000	1.326535000
1	-1.699574000	-1.929067000	0.921145000
1	-2.421413000	-4.157677000	0.869374000
1	-0.866217000	-5.010722000	1.206608000
16	-8.175176000	0.764466000	2.245357000
8	-7.320598000	-0.388373000	1.330141000
6	-9.864613000	0.762261000	1.460786000
6	-7.558931000	2.403414000	1.608622000
1	-10.328940000	-0.188476000	1.712478000
1	-6.499154000	2.456044000	1.847555000
1	-9.734402000	0.856174000	0.383472000
1	-10.437953000	1.592555000	1.869729000
1	-8.110102000	3.193338000	2.115809000
1	-7.711361000	2.439522000	0.531302000
16	4.775443000	-3.441186000	-0.947108000
8	5.308215000	-2.339545000	-2.126395000
6	4.969926000	-2.553926000	0.677147000
6	2.918304000	-3.375151000	-1.082251000
1	6.037062000	-2.458749000	0.902883000
1	2.649580000	-3.791834000	-2.050155000
1	4.465763000	-3.148912000	1.438841000
1	4.510993000	-1.570727000	0.579707000
1	2.602425000	-2.335128000	-1.011234000
1	2.495566000	-3.973221000	-0.277468000
16	-3.939394000	-1.697785000	2.866081000
8	-3.720513000	-2.635860000	1.461928000
6	-4.239615000	0.025698000	2.221647000

Xanthione

6	-5.673928000	-2.101518000	3.422214000
1	-3.364722000	0.303314000	1.637887000
1	-5.711465000	-3.180923000	3.554726000
1	-4.356173000	0.685122000	3.081477000
1	-5.142893000	0.005059000	1.609865000
1	-6.369204000	-1.746061000	2.655929000
1	-5.841675000	-1.593303000	4.371618000
16	7.626786000	1.052985000	-1.177286000
8	7.009785000	1.383356000	0.382727000
6	9.397905000	1.615124000	-1.048599000
6	7.889051000	-0.792901000	-1.206177000
1	9.374460000	2.676149000	-0.810291000
1	6.957357000	-1.257611000	-1.546325000
1	9.878584000	1.446295000	-2.011012000
1	9.888406000	1.050571000	-0.257606000
1	8.124133000	-1.109764000	-0.183688000
1	8.700728000	-0.995421000	-1.905300000

3. L_b

6	-3.256499000	-0.209289000	-1.497424000
6	-4.673103000	-0.150372000	-1.505912000
6	-5.276319000	1.093025000	-1.195387000
6	-4.513998000	2.194713000	-0.853112000
6	-3.105637000	2.146925000	-0.803729000
6	-2.526682000	0.903209000	-1.146745000
6	-2.260884000	3.231658000	-0.419568000
6	-0.842146000	3.064119000	-0.431336000
6	-0.303659000	1.805593000	-0.777470000
6	0.100349000	4.060584000	-0.105658000
6	1.463881000	3.819510000	-0.107538000

Xanthione

6	1.046817000	1.538441000	-0.784977000
6	1.981678000	2.541012000	-0.430899000
1	-0.252415000	5.054894000	0.146801000
1	1.358176000	0.548550000	-1.081323000
1	2.132021000	4.630268000	0.142923000
1	-2.715639000	-1.124597000	-1.714368000
1	-5.016595000	3.129427000	-0.627327000
1	-6.350480000	1.188898000	-1.209392000
8	-1.135917000	0.744584000	-1.131601000
16	-2.979885000	4.721609000	0.251311000
7	-5.429517000	-1.261853000	-1.802375000
7	3.330829000	2.269142000	-0.389456000
6	-4.792192000	-2.506912000	-2.246063000
1	-3.909797000	-2.714978000	-1.636334000
1	-5.507234000	-3.313972000	-2.065472000
6	-4.383220000	-2.496010000	-3.720669000
1	-3.707353000	-1.661014000	-3.926899000
1	-5.253415000	-2.403175000	-4.377068000
1	-3.856075000	-3.421818000	-3.969387000
6	-6.895117000	-1.203032000	-1.774475000
1	-7.250795000	-2.229558000	-1.649473000
1	-7.209694000	-0.661854000	-0.876016000
6	-7.519849000	-0.602475000	-3.037214000
1	-7.288279000	-1.209317000	-3.917126000
1	-7.149929000	0.410479000	-3.220159000
1	-8.608895000	-0.552991000	-2.934476000
6	3.838541000	0.927424000	-0.692615000
1	4.824123000	0.851602000	-0.227948000
1	3.192822000	0.190436000	-0.200265000
6	3.956790000	0.609995000	-2.184918000
1	4.667467000	1.290207000	-2.664189000
1	2.995164000	0.713333000	-2.696705000

Xanthione

1	4.318783000	-0.416709000	-2.309305000
6	4.314993000	3.325457000	-0.127498000
1	3.989315000	3.919458000	0.734142000
1	5.247739000	2.830060000	0.154637000
6	4.569501000	4.236257000	-1.331939000
1	3.648165000	4.713421000	-1.678301000
1	4.987195000	3.662686000	-2.164395000
1	5.283065000	5.023499000	-1.068303000
16	7.829783000	-0.845813000	3.141476000
8	7.957493000	-1.895906000	1.807818000
6	6.175469000	-0.003407000	2.946397000
6	8.909011000	0.609110000	2.696772000
1	5.407292000	-0.757515000	3.101615000
1	9.905235000	0.213667000	2.508215000
1	6.150867000	0.416479000	1.938102000
1	6.108732000	0.771002000	3.709969000
1	8.919862000	1.287875000	3.548855000
1	8.471724000	1.071369000	1.807728000
16	-0.662909000	-3.483310000	-0.539077000
8	-1.737853000	-3.085158000	-1.792288000
6	-0.687518000	-2.012612000	0.605734000
6	-1.608215000	-4.673970000	0.542875000
1	-0.418217000	-1.141832000	0.012183000
1	-1.907824000	-5.504026000	-0.093889000
1	0.034726000	-2.196705000	1.400368000
1	-1.707357000	-1.932666000	0.987222000
1	-2.466833000	-4.141762000	0.963094000
1	-0.929644000	-5.017182000	1.323617000
16	-8.143316000	0.879661000	2.183041000
8	-7.309722000	-0.322806000	1.313388000
6	-9.831993000	0.878625000	1.396902000
6	-7.497769000	2.483493000	1.487842000

Xanthione

1	-10.312310000	-0.054924000	1.680873000
1	-6.437928000	2.528500000	1.728143000
1	-9.700140000	0.933125000	0.317120000
1	-10.391465000	1.731997000	1.776360000
1	-8.038363000	3.300294000	1.963009000
1	-7.645143000	2.481302000	0.409307000
16	4.760619000	-3.480034000	-0.763092000
8	5.303333000	-2.453477000	-2.004110000
6	4.972881000	-2.504755000	0.807719000
6	2.904107000	-3.397781000	-0.899194000
1	6.042244000	-2.409534000	1.022486000
1	2.627035000	-3.877519000	-1.834938000
1	4.465673000	-3.049101000	1.604475000
1	4.525537000	-1.523106000	0.656795000
1	2.603689000	-2.350812000	-0.901503000
1	2.474823000	-3.931824000	-0.053868000
16	-3.942470000	-1.583887000	2.890739000
8	-3.744905000	-2.579659000	1.523864000
6	-4.231114000	0.117294000	2.184307000
6	-5.676192000	-1.950213000	3.474470000
1	-3.348824000	0.374103000	1.602406000
1	-5.719609000	-3.022338000	3.655357000
1	-4.356683000	0.804831000	3.020660000
1	-5.127413000	0.077022000	1.563238000
1	-6.372687000	-1.624983000	2.695904000
1	-5.835859000	-1.398799000	4.400913000
16	7.618280000	0.983768000	-1.255833000
8	7.009869000	1.395662000	0.288107000
6	9.386185000	1.563617000	-1.170189000
6	7.892144000	-0.859332000	-1.185860000
1	9.356682000	2.635658000	-0.988665000
1	6.960783000	-1.346870000	-1.493566000

Xanthione

1	9.861420000	1.346898000	-2.125607000
1	9.886008000	1.045507000	-0.353723000
1	8.135433000	-1.118969000	-0.149329000
1	8.700453000	-1.094613000	-1.878630000

VII. OPTIMISED GEOMETRIES - FORMAMIDE

A. Implicit

1. S_0

6	2.308129000	-0.785807000	-0.357445000
6	3.582129000	-0.205265000	-0.292813000
6	3.639165000	1.206281000	-0.110990000
6	2.502464000	1.952278000	-0.029872000
6	1.214902000	1.387667000	-0.105407000
6	1.175973000	-0.003110000	-0.272012000
6	0.000075000	2.152548000	-0.017465000
6	-1.214713000	1.387648000	-0.105236000
6	-1.175787000	-0.003127000	-0.271860000
6	-2.502302000	1.952212000	-0.029392000
6	-3.638988000	1.206196000	-0.110319000
6	-2.307933000	-0.785859000	-0.357145000
6	-3.581917000	-0.205347000	-0.292237000
1	-2.571760000	3.022216000	0.104447000
1	-2.166101000	-1.847906000	-0.471152000
1	-4.590362000	1.706620000	-0.033760000
1	2.166321000	-1.847867000	-0.471337000
1	2.571903000	3.022298000	0.103860000
1	4.590513000	1.706803000	-0.034824000
8	0.000097000	-0.676189000	-0.352167000
16	0.000073000	3.833066000	0.175778000

Xanthione

7	4.712424000	-0.948140000	-0.411522000
7	-4.712191000	-0.948139000	-0.410872000
6	4.642149000	-2.396813000	-0.562830000
1	3.860024000	-2.639333000	-1.282838000
1	5.578670000	-2.717094000	-1.015547000
6	4.416513000	-3.154597000	0.739579000
1	3.504602000	-2.827584000	1.238624000
1	5.249641000	-3.008881000	1.426071000
1	4.327500000	-4.222562000	0.536670000
6	6.034611000	-0.346206000	-0.272190000
1	6.740892000	-1.017713000	-0.756713000
1	6.066473000	0.583981000	-0.839253000
6	6.464488000	-0.103395000	1.168887000
1	6.534766000	-1.040497000	1.719896000
1	5.760315000	0.544503000	1.690573000
1	7.444118000	0.375869000	1.188151000
6	-4.642177000	-2.396703000	-0.562991000
1	-5.578526000	-2.716563000	-1.016389000
1	-3.859720000	-2.639050000	-1.282676000
6	-4.417374000	-3.155079000	0.739200000
1	-5.250795000	-3.009454000	1.425357000
1	-3.505613000	-2.828385000	1.238748000
1	-4.328482000	-4.222993000	0.535951000
6	-6.034337000	-0.345900000	-0.272535000
1	-6.065614000	0.584154000	-0.839874000
1	-6.740410000	-1.017381000	-0.757411000
6	-6.465102000	-0.102753000	1.168193000
1	-5.761187000	0.545137000	1.690240000
1	-6.535853000	-1.039738000	1.719346000
1	-7.444694000	0.376626000	1.186771000

Xanthione

2. L_a

6	2.322861000	-0.779263000	-0.358955000
6	3.602713000	-0.195189000	-0.306488000
6	3.658335000	1.202625000	-0.143172000
6	2.507805000	1.956278000	-0.061705000
6	1.229253000	1.387304000	-0.122260000
6	1.189584000	-0.007184000	-0.274843000
6	-0.000022000	2.111732000	-0.028615000
6	-1.229275000	1.387279000	-0.122319000
6	-1.189567000	-0.007218000	-0.274868000
6	-2.507842000	1.956225000	-0.061853000
6	-3.658352000	1.202541000	-0.143371000
6	-2.322822000	-0.779322000	-0.359023000
6	-3.602685000	-0.195271000	-0.306624000
1	-2.601096000	3.029250000	0.056434000
1	-2.186515000	-1.843643000	-0.464289000
1	-4.606910000	1.710866000	-0.080821000
1	2.186579000	-1.843590000	-0.464190000
1	2.601021000	3.029301000	0.056625000
1	4.606878000	1.710958000	-0.080517000
8	0.000018000	-0.684552000	-0.337964000
16	-0.000052000	3.810134000	0.253867000
7	4.737761000	-0.955264000	-0.431858000
7	-4.737715000	-0.955375000	-0.431995000
6	4.659337000	-2.403890000	-0.535147000
1	3.876602000	-2.669497000	-1.247436000
1	5.594285000	-2.744969000	-0.977535000
6	4.429559000	-3.126340000	0.787956000
1	3.519920000	-2.779239000	1.277835000
1	5.263458000	-2.966274000	1.470613000
1	4.333393000	-4.199517000	0.616309000

Xanthione

6	6.055601000	-0.353753000	-0.299505000
1	6.765136000	-1.029995000	-0.774336000
1	6.087120000	0.569907000	-0.879381000
6	6.492137000	-0.084506000	1.136419000
1	6.576471000	-1.012863000	1.700481000
1	5.782220000	0.561728000	1.652370000
1	7.466148000	0.407098000	1.145287000
6	-4.659246000	-2.404021000	-0.534795000
1	-5.594107000	-2.745255000	-0.977250000
1	-3.876386000	-2.669819000	-1.246881000
6	-4.429702000	-3.126066000	0.788569000
1	-5.263765000	-2.965857000	1.470994000
1	-3.520188000	-2.778776000	1.278546000
1	-4.333439000	-4.199288000	0.617251000
6	-6.055545000	-0.353859000	-0.299701000
1	-6.087067000	0.569740000	-0.879675000
1	-6.765079000	-1.030158000	-0.774453000
6	-6.492073000	-0.084405000	1.136197000
1	-5.782099000	0.561841000	1.652052000
1	-6.576506000	-1.012668000	1.700398000
1	-7.466039000	0.407293000	1.144983000

3. L_b

6	2.322080000	-0.776996000	-0.374267000
6	3.600742000	-0.191355000	-0.295660000
6	3.646915000	1.208474000	-0.108117000
6	2.496923000	1.954537000	-0.028907000
6	1.213731000	1.388762000	-0.115989000
6	1.191968000	-0.003206000	-0.292510000
6	-0.008718000	2.134547000	-0.029398000

Xanthione

6	-1.215610000	1.386462000	-0.112407000
6	-1.177681000	-0.032239000	-0.294810000
6	-2.517681000	1.957538000	-0.022979000
6	-3.653487000	1.206686000	-0.108834000
6	-2.299017000	-0.794295000	-0.382242000
6	-3.593263000	-0.202397000	-0.296723000
1	-2.577635000	3.025537000	0.119402000
1	-2.169718000	-1.855916000	-0.513760000
1	-4.606541000	1.703954000	-0.029965000
1	2.186918000	-1.839227000	-0.499029000
1	2.563343000	3.023952000	0.112778000
1	4.593828000	1.716941000	-0.022025000
8	0.005600000	-0.692943000	-0.388095000
16	-0.064403000	3.860656000	0.181120000
7	4.736664000	-0.943459000	-0.412773000
7	-4.708201000	-0.955780000	-0.398636000
6	4.665735000	-2.391015000	-0.546928000
1	3.893481000	-2.643908000	-1.274658000
1	5.608410000	-2.719126000	-0.981807000
6	4.419853000	-3.136878000	0.760131000
1	3.500816000	-2.803250000	1.241483000
1	5.242551000	-2.984922000	1.457828000
1	4.332416000	-4.206923000	0.566638000
6	6.052970000	-0.339545000	-0.263963000
1	6.766682000	-1.006136000	-0.745439000
1	6.083149000	0.593719000	-0.827407000
6	6.476673000	-0.094853000	1.180080000
1	6.557330000	-1.032530000	1.728818000
1	5.762145000	0.542550000	1.700456000
1	7.450223000	0.396835000	1.203656000
6	-4.637764000	-2.406455000	-0.566278000
1	-5.579144000	-2.718845000	-1.011565000

Xanthione

1	-3.859911000	-2.640121000	-1.291220000
6	-4.403038000	-3.164912000	0.735188000
1	-5.226947000	-3.016434000	1.431077000
1	-3.481441000	-2.846417000	1.220907000
1	-4.326390000	-4.231158000	0.521250000
6	-6.037764000	-0.365244000	-0.253595000
1	-6.073748000	0.563827000	-0.820726000
1	-6.739268000	-1.046461000	-0.728740000
6	-6.448600000	-0.124980000	1.194847000
1	-5.743036000	0.528938000	1.705786000
1	-6.510468000	-1.061238000	1.746958000
1	-7.430438000	0.348283000	1.216170000

B. S=2

1. S_0

6	1.602754000	-1.203072000	-0.234607000
6	2.976395000	-0.902168000	-0.333426000
6	3.337179000	0.473458000	-0.485741000
6	2.377149000	1.451800000	-0.539421000
6	0.992928000	1.171778000	-0.444576000
6	0.666160000	-0.189794000	-0.297637000
6	-0.034566000	2.177671000	-0.487701000
6	-1.390161000	1.715461000	-0.364720000
6	-1.680672000	0.343113000	-0.220070000
6	-2.516693000	2.571069000	-0.369942000
6	-3.798329000	2.101374000	-0.244754000
6	-2.954189000	-0.169065000	-0.085555000
6	-4.063523000	0.703359000	-0.106209000
1	-2.338859000	3.633141000	-0.474215000

Xanthione

1	-3.065014000	-1.239393000	0.030265000
1	-4.611814000	2.810928000	-0.247214000
1	1.246774000	-2.210859000	-0.089267000
1	2.669639000	2.486795000	-0.664546000
1	4.376314000	0.762821000	-0.571312000
8	-0.655830000	-0.594305000	-0.208649000
16	0.347586000	3.868559000	-0.673021000
7	3.926322000	-1.883612000	-0.299704000
7	-5.340730000	0.228254000	-0.006316000
6	3.544464000	-3.295316000	-0.159715000
1	2.690680000	-3.496826000	-0.814080000
1	4.373901000	-3.892093000	-0.544427000
6	3.227189000	-3.716834000	1.276708000
1	2.427602000	-3.106642000	1.705227000
1	4.107893000	-3.617984000	1.915903000
1	2.906271000	-4.762446000	1.298018000
6	5.361941000	-1.559704000	-0.279548000
1	5.892474000	-2.440144000	-0.648357000
1	5.565719000	-0.751743000	-0.984814000
6	5.880979000	-1.177279000	1.106465000
1	5.792081000	-2.012492000	1.805832000
1	5.324319000	-0.328902000	1.512795000
1	6.933750000	-0.888655000	1.044704000
6	-5.597832000	-1.207073000	0.190706000
1	-6.618362000	-1.398438000	-0.148068000
1	-4.933745000	-1.788402000	-0.452368000
6	-5.432796000	-1.666244000	1.639761000
1	-6.158627000	-1.178289000	2.295644000
1	-4.429005000	-1.440314000	2.009180000
1	-5.579118000	-2.747771000	1.705925000
6	-6.494235000	1.138361000	0.021071000
1	-6.368922000	1.901733000	-0.752218000

Xanthione

1	-7.369180000	0.552324000	-0.266996000
6	-6.734688000	1.791906000	1.383154000
1	-5.860543000	2.361563000	1.710407000
1	-6.952330000	1.038278000	2.144123000
1	-7.586211000	2.476491000	1.327049000
8	6.334960000	1.745797000	-0.400792000
6	6.381374000	2.475025000	0.615166000
1	7.294722000	3.016975000	0.880985000
8	-3.219709000	-3.457514000	-0.191190000
6	-2.331046000	-3.948132000	-0.926238000
1	-2.430105000	-4.960800000	-1.331078000
7	5.358856000	2.671326000	1.465356000
1	5.457727000	3.272958000	2.267634000
1	4.467710000	2.219571000	1.314144000
7	-1.205751000	-3.312127000	-1.285273000
1	-1.019090000	-2.370557000	-0.951824000
1	-0.532959000	-3.754633000	-1.891119000

2. L_a

6	1.823361000	1.443078000	0.179260000
6	3.204961000	1.176922000	0.326268000
6	3.576293000	-0.161169000	0.600839000
6	2.627203000	-1.165450000	0.712653000
6	1.250135000	-0.916860000	0.544804000
6	0.901375000	0.426650000	0.297018000
6	0.224565000	-1.909488000	0.592605000
6	-1.143797000	-1.520447000	0.455266000
6	-1.459537000	-0.163927000	0.228071000
6	-2.246405000	-2.395728000	0.506409000
6	-3.549293000	-1.953534000	0.349983000

Xanthione

6	-2.741566000	0.307648000	0.063680000
6	-3.838336000	-0.585093000	0.131558000
1	-2.073701000	-3.453321000	0.674169000
1	-2.876758000	1.367784000	-0.105762000
1	-4.345619000	-2.681699000	0.392380000
1	1.454884000	2.433533000	-0.041082000
1	2.961681000	-2.174644000	0.930310000
1	4.615397000	-0.422448000	0.739124000
8	-0.440410000	0.797599000	0.156641000
16	0.645742000	-3.640036000	0.712144000
7	4.142114000	2.185000000	0.225165000
7	-5.133084000	-0.125382000	0.001034000
6	3.737146000	3.562429000	-0.068353000
1	2.858765000	3.813793000	0.534920000
1	4.540914000	4.216671000	0.277321000
6	3.457689000	3.829374000	-1.549599000
1	2.682672000	3.160350000	-1.933779000
1	4.360547000	3.680308000	-2.147796000
1	3.120054000	4.860380000	-1.692381000
6	5.576072000	1.883112000	0.276445000
1	6.094141000	2.821371000	0.486261000
1	5.776656000	1.225591000	1.129091000
6	6.130402000	1.266172000	-1.010214000
1	6.017874000	1.955602000	-1.851063000
1	5.610879000	0.337378000	-1.261636000
1	7.194532000	1.039730000	-0.893181000
6	-5.400263000	1.275488000	-0.345023000
1	-6.435679000	1.483281000	-0.064270000
1	-4.771042000	1.932141000	0.261624000
6	-5.190866000	1.596733000	-1.826759000
1	-5.876539000	1.022755000	-2.455843000
1	-4.168051000	1.364668000	-2.136465000

Xanthione

1	-5.365784000	2.660824000	-2.010766000
6	-6.267581000	-1.051252000	0.046709000
1	-6.119702000	-1.757852000	0.869749000
1	-7.152381000	-0.463947000	0.303865000
6	-6.515483000	-1.808178000	-1.260921000
1	-5.629756000	-2.372812000	-1.564959000
1	-6.770109000	-1.117005000	-2.068613000
1	-7.344593000	-2.512225000	-1.140447000
8	3.744349000	-4.247605000	0.407141000
6	4.122114000	-4.231808000	-0.787168000
1	4.518523000	-5.134474000	-1.264108000
8	-3.068994000	3.664204000	0.240064000
6	-2.213833000	4.037611000	1.077202000
1	-2.341494000	4.976380000	1.627581000
7	4.091090000	-3.155718000	-1.590033000
1	4.411565000	-3.211680000	-2.543493000
1	3.742527000	-2.268615000	-1.252284000
7	-1.094267000	3.365541000	1.382390000
1	-0.874598000	2.480584000	0.928954000
1	-0.454354000	3.724813000	2.073224000

3. L_b

6	1.623680000	-1.218827000	-0.277691000
6	3.005854000	-0.912948000	-0.355128000
6	3.360461000	0.457544000	-0.515133000
6	2.393914000	1.436410000	-0.586591000
6	1.004878000	1.156536000	-0.507432000
6	0.687968000	-0.216200000	-0.359035000
6	-0.020591000	2.157568000	-0.560233000
6	-1.371480000	1.700501000	-0.412890000

Xanthione

6	-1.671369000	0.312782000	-0.266290000
6	-2.506351000	2.567750000	-0.389593000
6	-3.788081000	2.102408000	-0.238336000
6	-2.933991000	-0.184197000	-0.106913000
6	-4.052883000	0.705574000	-0.093127000
1	-2.316954000	3.626599000	-0.496140000
1	-3.056100000	-1.256042000	-0.004492000
1	-4.599293000	2.815025000	-0.225628000
1	1.271977000	-2.229081000	-0.134448000
1	2.681589000	2.471998000	-0.718335000
1	4.399318000	0.752948000	-0.588774000
8	-0.646485000	-0.636075000	-0.292690000
16	0.342085000	3.895333000	-0.759081000
7	3.958373000	-1.897723000	-0.291262000
7	-5.318518000	0.223492000	0.047988000
6	3.578939000	-3.310527000	-0.170918000
1	2.734766000	-3.507526000	-0.838248000
1	4.415868000	-3.901878000	-0.547999000
6	3.241407000	-3.746008000	1.258137000
1	2.433362000	-3.141417000	1.678511000
1	4.112020000	-3.651335000	1.911477000
1	2.923592000	-4.792741000	1.262974000
6	5.388077000	-1.564700000	-0.209484000
1	5.941939000	-2.450567000	-0.526567000
1	5.620586000	-0.772024000	-0.924098000
6	5.834004000	-1.141043000	1.191752000
1	5.718215000	-1.959339000	1.906697000
1	5.250416000	-0.287965000	1.546384000
1	6.885951000	-0.843879000	1.172344000
6	-5.572844000	-1.213702000	0.255565000
1	-6.606901000	-1.397808000	-0.039301000
1	-4.932930000	-1.798556000	-0.406898000

Xanthione

6	-5.349484000	-1.663500000	1.701768000
1	-6.042821000	-1.168994000	2.386137000
1	-4.328014000	-1.448155000	2.024699000
1	-5.503857000	-2.743257000	1.770231000
6	-6.477778000	1.128487000	0.094715000
1	-6.365317000	1.889680000	-0.681485000
1	-7.355067000	0.536947000	-0.169583000
6	-6.681978000	1.782220000	1.464769000
1	-5.800010000	2.351554000	1.768807000
1	-6.886193000	1.031089000	2.231050000
1	-7.533873000	2.466340000	1.420583000
8	6.324738000	1.792052000	-0.290980000
6	6.270184000	2.517904000	0.727109000
1	7.152488000	3.059610000	1.083406000
8	-3.260137000	-3.386934000	-0.241141000
6	-2.377224000	-3.926733000	-0.950445000
1	-2.504792000	-4.947458000	-1.325074000
7	5.169105000	2.710590000	1.473907000
1	5.188707000	3.310539000	2.283201000
1	4.296667000	2.259782000	1.234287000
7	-1.229781000	-3.336952000	-1.313087000
1	-1.015116000	-2.391126000	-1.004329000
1	-0.564652000	-3.820291000	-1.895883000

C. S=4

1. S_0

6	-2.251140000	-0.305668000	0.200248000
6	-3.418940000	0.470442000	0.361487000
6	-3.253204000	1.885043000	0.471831000

Xanthione

6	-2.008292000	2.457851000	0.440472000
6	-0.825383000	1.699310000	0.286381000
6	-1.011773000	0.307087000	0.168182000
6	0.489807000	2.278429000	0.234736000
6	1.582735000	1.368315000	0.042155000
6	1.363706000	-0.020595000	-0.079041000
6	2.934252000	1.777019000	-0.048112000
6	3.962547000	0.891502000	-0.244010000
6	2.368730000	-0.945048000	-0.281641000
6	3.711129000	-0.512509000	-0.364791000
1	3.142781000	2.835794000	0.036738000
1	2.101454000	-1.991014000	-0.363048000
1	4.972726000	1.271940000	-0.297810000
1	-2.295817000	-1.377437000	0.084633000
1	-1.922614000	3.532617000	0.528585000
1	-4.102829000	2.542232000	0.576342000
8	0.076181000	-0.537074000	0.003730000
16	0.739568000	3.997981000	0.392437000
7	-4.659962000	-0.097769000	0.413869000
7	4.735008000	-1.395021000	-0.548809000
6	-4.835282000	-1.550281000	0.275808000
1	-4.083076000	-2.068435000	0.876430000
1	-5.804358000	-1.796927000	0.715353000
6	-4.787682000	-2.044534000	-1.171347000
1	-3.833847000	-1.798617000	-1.646126000
1	-5.588029000	-1.595842000	-1.765437000
1	-4.912269000	-3.130983000	-1.199221000
6	-5.871289000	0.729665000	0.516928000
1	-6.661935000	0.087791000	0.910060000
1	-5.711284000	1.511592000	1.264277000
6	-6.316233000	1.343084000	-0.811649000
1	-6.563782000	0.563637000	-1.536869000

Xanthione

1	-5.534269000	1.973054000	-1.243933000
1	-7.205136000	1.962631000	-0.659756000
6	4.468034000	-2.825255000	-0.768427000
1	5.385175000	-3.362796000	-0.517620000
1	3.695052000	-3.166130000	-0.077639000
6	4.042281000	-3.155508000	-2.199650000
1	4.835680000	-2.923803000	-2.915045000
1	3.149206000	-2.590677000	-2.480352000
1	3.802922000	-4.219280000	-2.278464000
6	6.128346000	-0.935059000	-0.665054000
1	6.320780000	-0.155867000	0.075817000
1	6.764208000	-1.783566000	-0.403005000
6	6.493784000	-0.424341000	-2.060193000
1	5.844683000	0.404002000	-2.358239000
1	6.401874000	-1.215233000	-2.808976000
1	7.527260000	-0.065064000	-2.067932000
8	-4.229130000	4.922491000	0.065788000
6	-4.302838000	5.037117000	-1.177812000
1	-4.829575000	5.878753000	-1.640432000
8	1.657684000	-4.195103000	-0.167134000
6	0.497323000	-4.409250000	0.267162000
1	0.154441000	-5.433858000	0.453545000
7	-3.766157000	4.173975000	-2.058730000
1	-3.855236000	4.318277000	-3.051801000
1	-3.254382000	3.366057000	-1.731661000
7	-0.409510000	-3.468225000	0.539843000
1	-0.185560000	-2.490339000	0.383685000
1	-1.346186000	-3.718522000	0.872585000
8	-3.024437000	-4.105556000	1.300532000
6	-3.669762000	-5.068256000	0.815429000
1	-3.185175000	-5.814096000	0.180670000
8	6.929660000	2.009459000	0.842914000

Xanthione

6	6.637793000	2.358702000	2.009582000
1	7.375783000	2.849273000	2.653001000
7	5.437926000	2.181204000	2.586041000
1	4.683110000	1.743571000	2.075053000
1	5.264304000	2.486935000	3.530105000
7	-4.975842000	-5.266664000	1.018736000
1	-5.455726000	-6.053446000	0.610390000
1	-5.505232000	-4.633751000	1.601769000

2. *L_a*

6	2.418042000	0.209713000	0.161414000
6	3.541075000	-0.633716000	0.342736000
6	3.288811000	-2.014776000	0.520768000
6	1.998465000	-2.518475000	0.512140000
6	0.874822000	-1.694316000	0.305510000
6	1.143879000	-0.317641000	0.150824000
6	-0.471892000	-2.159537000	0.229101000
6	-1.534303000	-1.224136000	0.045309000
6	-1.236404000	0.149820000	-0.091595000
6	-2.899660000	-1.559535000	-0.038002000
6	-3.887083000	-0.607201000	-0.236628000
6	-2.191402000	1.121547000	-0.292447000
6	-3.561665000	0.765996000	-0.361658000
1	-3.193920000	-2.600205000	0.050476000
1	-1.868239000	2.150848000	-0.386685000
1	-4.915464000	-0.936058000	-0.284662000
1	2.532565000	1.272687000	0.011541000
1	1.854794000	-3.583570000	0.663387000
1	4.105663000	-2.703375000	0.680471000
8	0.094858000	0.591944000	-0.032614000

Xanthione

16	-0.827352000	-3.909408000	0.259928000
7	4.825179000	-0.128415000	0.360733000
7	-4.538352000	1.723392000	-0.534640000
6	5.076796000	1.307103000	0.205078000
1	4.351420000	1.870994000	0.797821000
1	6.056393000	1.510306000	0.646075000
6	5.061471000	1.792843000	-1.246683000
1	4.098969000	1.587957000	-1.723989000
1	5.841775000	1.299163000	-1.832402000
1	5.239524000	2.871863000	-1.285552000
6	5.981977000	-1.028105000	0.404759000
1	6.844230000	-0.426004000	0.698980000
1	5.840040000	-1.767149000	1.200685000
6	6.275773000	-1.731538000	-0.922812000
1	6.509386000	-1.003213000	-1.703858000
1	5.419349000	-2.323901000	-1.256701000
1	7.132259000	-2.403813000	-0.813768000
6	-4.182621000	3.124556000	-0.788422000
1	-5.061821000	3.726377000	-0.544343000
1	-3.385145000	3.433992000	-0.109697000
6	-3.745796000	3.404003000	-2.228251000
1	-4.556400000	3.206810000	-2.935046000
1	-2.890921000	2.779803000	-2.502607000
1	-3.442179000	4.449548000	-2.330651000
6	-5.951933000	1.343921000	-0.636018000
1	-6.181064000	0.583275000	0.115188000
1	-6.539715000	2.228154000	-0.376187000
6	-6.366399000	0.839691000	-2.021302000
1	-5.761535000	-0.020424000	-2.322545000
1	-6.247555000	1.620007000	-2.777695000
1	-7.416240000	0.530267000	-2.012134000
8	1.754425000	-5.769498000	0.082737000

Xanthione

6	2.210534000	-5.886626000	-1.078237000
1	2.253797000	-6.864281000	-1.569778000
8	-1.255973000	4.325318000	-0.229697000
6	-0.089556000	4.455373000	0.221586000
1	0.319456000	5.453113000	0.421872000
7	2.682330000	-4.875261000	-1.825514000
1	3.036006000	-5.038432000	-2.754710000
1	2.688276000	-3.928990000	-1.469131000
7	0.748287000	3.453470000	0.497788000
1	0.464742000	2.491332000	0.330391000
1	1.693517000	3.642948000	0.844947000
8	3.386455000	3.953985000	1.296776000
6	4.101382000	4.845710000	0.775584000
1	3.675149000	5.596446000	0.105458000
8	-6.894464000	-1.523648000	1.051581000
6	-6.523654000	-1.748246000	2.226359000
1	-7.228005000	-2.117525000	2.979718000
7	-5.273446000	-1.570603000	2.684032000
1	-4.544035000	-1.240051000	2.065337000
1	-5.038165000	-1.770855000	3.642941000
7	5.417756000	4.958226000	0.980145000
1	5.955171000	5.690353000	0.542823000
1	5.896911000	4.313111000	1.592457000

3. L_b

6	-2.191750000	-0.343932000	0.168843000
6	-3.378138000	0.425805000	0.287983000
6	-3.230522000	1.838254000	0.400327000
6	-1.984564000	2.423455000	0.387218000
6	-0.784280000	1.678483000	0.258138000

Xanthione

6	-0.963983000	0.275521000	0.157630000
6	0.520293000	2.269924000	0.220390000
6	1.625320000	1.375717000	0.040278000
6	1.429336000	-0.034531000	-0.057863000
6	2.982993000	1.806993000	-0.060766000
6	4.026920000	0.932905000	-0.237948000
6	2.444199000	-0.934757000	-0.235425000
6	3.794994000	-0.474404000	-0.328029000
1	3.162789000	2.872110000	-0.001497000
1	2.202728000	-1.990733000	-0.282075000
1	5.032854000	1.326620000	-0.295184000
1	-2.225974000	-1.418222000	0.068223000
1	-1.889207000	3.497672000	0.484762000
1	-4.097897000	2.477707000	0.501409000
8	0.143499000	-0.574525000	0.038013000
16	0.781158000	4.032298000	0.357892000
7	-4.615962000	-0.163868000	0.302032000
7	4.822006000	-1.353154000	-0.491933000
6	-4.765196000	-1.622140000	0.222341000
1	-4.019855000	-2.101598000	0.862028000
1	-5.742629000	-1.864865000	0.646137000
6	-4.670864000	-2.182400000	-1.200248000
1	-3.709578000	-1.939322000	-1.660948000
1	-5.463992000	-1.780736000	-1.835572000
1	-4.773546000	-3.271165000	-1.176499000
6	-5.840891000	0.648370000	0.264319000
1	-6.651098000	0.021816000	0.642462000
1	-5.744021000	1.490744000	0.952792000
6	-6.187076000	1.164447000	-1.134276000
1	-6.411045000	0.339382000	-1.814877000
1	-5.360046000	1.742643000	-1.553418000
1	-7.061836000	1.818270000	-1.082605000

Xanthione

6	4.575169000	-2.790336000	-0.701699000
1	5.504440000	-3.308891000	-0.461516000
1	3.810343000	-3.140409000	-0.006959000
6	4.140818000	-3.123856000	-2.132011000
1	4.918821000	-2.870949000	-2.856129000
1	3.228120000	-2.584842000	-2.398019000
1	3.931859000	-4.194152000	-2.203616000
6	6.216286000	-0.885965000	-0.573964000
1	6.373845000	-0.082888000	0.148561000
1	6.849143000	-1.723798000	-0.276543000
6	6.611046000	-0.403376000	-1.973219000
1	5.958529000	0.407689000	-2.306839000
1	6.557577000	-1.212946000	-2.704860000
1	7.637877000	-0.027899000	-1.951130000
8	-5.530031000	4.134175000	0.206686000
6	-5.279601000	4.742576000	-0.857982000
1	-5.934668000	5.541730000	-1.220035000
8	1.814192000	-4.117592000	-0.031185000
6	0.652178000	-4.372023000	0.380676000
1	0.348168000	-5.407078000	0.574521000
7	-4.222264000	4.497568000	-1.651319000
1	-4.074506000	5.024281000	-2.497554000
1	-3.550565000	3.781522000	-1.410483000
7	-0.294890000	-3.463378000	0.616949000
1	-0.103398000	-2.478979000	0.449951000
1	-1.230939000	-3.740304000	0.929873000
8	-2.921870000	-4.120064000	1.321102000
6	-3.538107000	-5.128997000	0.895832000
1	-3.022696000	-5.915832000	0.339799000
8	6.947596000	2.110898000	0.630327000
6	6.732957000	2.728000000	1.699202000
1	7.527728000	3.300601000	2.187894000

Xanthione

7	5.555665000	2.760519000	2.343631000
1	4.752642000	2.267869000	1.977098000
1	5.445846000	3.280801000	3.199560000
7	-4.847321000	-5.331771000	1.073031000
1	-5.302768000	-6.156173000	0.713858000
1	-5.404520000	-4.663419000	1.586419000

D. S=6

1. S_0

6	2.196479000	-0.043450000	-0.079974000
6	3.417474000	0.649495000	0.067679000
6	3.349116000	2.065686000	0.234658000
6	2.149114000	2.725494000	0.212933000
6	0.914981000	2.053948000	0.046753000
6	1.002752000	0.653097000	-0.088875000
6	-0.358714000	2.719807000	0.023332000
6	-1.518401000	1.884507000	-0.116978000
6	-1.396602000	0.483059000	-0.239441000
6	-2.843106000	2.380862000	-0.133966000
6	-3.931117000	1.557689000	-0.254801000
6	-2.469457000	-0.377553000	-0.361115000
6	-3.782726000	0.142694000	-0.378223000
1	-3.001350000	3.446848000	-0.044329000
1	-2.274594000	-1.438903000	-0.448660000
1	-4.913067000	2.002030000	-0.248214000
1	2.163459000	-1.116792000	-0.180746000
1	2.142065000	3.800369000	0.329287000
1	4.240423000	2.651119000	0.393885000
8	-0.141726000	-0.114662000	-0.244678000

Xanthione

16	-0.486363000	4.456077000	0.160587000
7	4.618230000	-0.001502000	0.042225000
7	-4.872598000	-0.669848000	-0.515541000
6	4.674240000	-1.464189000	-0.077963000
1	3.987233000	-1.791119000	-0.863226000
1	5.679062000	-1.720577000	-0.419886000
6	4.375296000	-2.199751000	1.230508000
1	3.427918000	-1.868189000	1.664072000
1	5.174595000	-2.034041000	1.956044000
1	4.297017000	-3.276287000	1.046532000
6	5.883127000	0.732812000	0.188808000
1	6.665308000	0.112671000	-0.254011000
1	5.835525000	1.644972000	-0.413325000
6	6.246439000	1.058816000	1.637842000
1	6.458699000	0.142263000	2.192091000
1	5.437366000	1.599845000	2.136321000
1	7.143018000	1.686977000	1.665850000
6	-4.720284000	-2.132367000	-0.561288000
1	-5.615178000	-2.530720000	-1.045697000
1	-3.870525000	-2.391811000	-1.195133000
6	-4.535769000	-2.774012000	0.814588000
1	-5.422764000	-2.644454000	1.440100000
1	-3.675603000	-2.338193000	1.330379000
1	-4.340852000	-3.843779000	0.696039000
6	-6.235569000	-0.118667000	-0.514157000
1	-6.261304000	0.774157000	-1.145928000
1	-6.877770000	-0.857035000	-0.999164000
6	-6.779839000	0.198834000	0.880183000
1	-6.111939000	0.876069000	1.420750000
1	-6.902848000	-0.713605000	1.467745000
1	-7.759957000	0.680027000	0.795982000
8	4.427031000	5.055158000	0.370987000

Xanthione

6	5.104070000	5.238670000	-0.663380000
1	4.718198000	5.823438000	-1.504757000
8	-1.922316000	-3.607528000	-0.982854000
6	-0.720493000	-3.852002000	-1.258699000
1	-0.415949000	-4.857202000	-1.573467000
7	6.342277000	4.749646000	-0.858373000
1	6.847546000	4.927507000	-1.711829000
1	6.800338000	4.217037000	-0.132901000
7	0.278638000	-2.967574000	-1.206127000
1	0.092531000	-2.012703000	-0.915156000
1	1.237118000	-3.244545000	-1.437429000
8	2.936119000	-3.680213000	-1.758960000
6	3.572725000	-4.711284000	-1.431867000
1	3.068380000	-5.579407000	-1.000673000
8	-5.045334000	4.749360000	-0.091356000
6	-6.066534000	4.459799000	-0.748179000
1	-6.206232000	4.808158000	-1.776919000
7	-7.080391000	3.701708000	-0.286382000
1	-7.065174000	3.353016000	0.661380000
1	-7.888665000	3.504189000	-0.855046000
7	4.897478000	-4.842660000	-1.570018000
1	5.368558000	-5.695376000	-1.310591000
1	5.444511000	-4.099606000	-1.981706000
8	-7.781427000	-2.845595000	2.098502000
6	-8.101588000	-3.553713000	1.118972000
1	-7.356671000	-3.887076000	0.389043000
8	7.624075000	-1.980534000	2.202236000
6	8.139936000	-2.529440000	1.205462000
1	9.087011000	-2.175286000	0.784944000
7	7.611752000	-3.581176000	0.551766000
1	8.079316000	-3.991381000	-0.241085000
1	6.745172000	-3.995217000	0.863549000

Xanthione

7	-9.351760000	-3.972016000	0.857306000
1	-10.111968000	-3.717197000	1.470949000
1	-9.556183000	-4.544417000	0.053675000

2. L_a

6	2.225884000	-0.035767000	-0.093606000
6	3.445364000	0.668903000	0.056247000
6	3.365661000	2.070542000	0.225684000
6	2.148668000	2.727747000	0.209808000
6	0.928950000	2.043636000	0.043309000
6	1.025654000	0.642721000	-0.100440000
6	-0.357984000	2.659610000	0.021207000
6	-1.529815000	1.858360000	-0.122263000
6	-1.401337000	0.458265000	-0.255006000
6	-2.846611000	2.355948000	-0.133378000
6	-3.943613000	1.523066000	-0.263510000
6	-2.474677000	-0.396230000	-0.381800000
6	-3.793261000	0.123086000	-0.397239000
1	-3.029126000	3.420377000	-0.031511000
1	-2.281961000	-1.457667000	-0.476259000
1	-4.925179000	1.969388000	-0.254242000
1	2.206922000	-1.109462000	-0.201845000
1	2.156517000	3.805436000	0.331471000
1	4.252309000	2.663357000	0.384592000
8	-0.128235000	-0.135996000	-0.265114000
16	-0.503902000	4.430646000	0.177672000
7	4.659837000	0.014054000	0.022791000
7	-4.887121000	-0.705842000	-0.549656000
6	4.718992000	-1.446664000	-0.070094000
1	4.036059000	-1.792569000	-0.851689000

Xanthione

1	5.726085000	-1.708050000	-0.403680000
6	4.417375000	-2.165485000	1.248196000
1	3.464133000	-1.833690000	1.668966000
1	5.209293000	-1.980087000	1.977478000
1	4.348730000	-3.246281000	1.083501000
6	5.914628000	0.759674000	0.150353000
1	6.700979000	0.147789000	-0.298966000
1	5.849036000	1.669704000	-0.455541000
6	6.297928000	1.104544000	1.591341000
1	6.534461000	0.196473000	2.150413000
1	5.486092000	1.633309000	2.098673000
1	7.182693000	1.750310000	1.601611000
6	-4.725942000	-2.163693000	-0.586168000
1	-5.617484000	-2.571693000	-1.070847000
1	-3.874030000	-2.423968000	-1.217742000
6	-4.539376000	-2.805350000	0.790594000
1	-5.426949000	-2.677657000	1.416022000
1	-3.681758000	-2.363888000	1.306231000
1	-4.339936000	-3.875107000	0.675844000
6	-6.245856000	-0.157176000	-0.537135000
1	-6.275695000	0.738486000	-1.167005000
1	-6.894309000	-0.893042000	-1.019534000
6	-6.787697000	0.163472000	0.859094000
1	-6.109482000	0.829611000	1.400512000
1	-6.920250000	-0.749229000	1.444479000
1	-7.761471000	0.658775000	0.779002000
8	4.244472000	5.196145000	0.284605000
6	4.883304000	5.228727000	-0.788744000
1	4.464398000	5.683844000	-1.692275000
8	-1.897967000	-3.634090000	-1.009394000
6	-0.688926000	-3.855263000	-1.273602000
1	-0.364503000	-4.853082000	-1.593291000

Xanthione

7	6.117545000	4.718318000	-0.955486000
1	6.592120000	4.774386000	-1.842693000
1	6.603461000	4.293839000	-0.178485000
7	0.296125000	-2.956532000	-1.203320000
1	0.098227000	-2.004079000	-0.906567000
1	1.258519000	-3.223521000	-1.428225000
8	2.956960000	-3.679493000	-1.749025000
6	3.594193000	-4.697064000	-1.384105000
1	3.089589000	-5.552590000	-0.928502000
8	-4.954675000	4.788023000	0.016809000
6	-6.004580000	4.516854000	-0.601914000
1	-6.188877000	4.894647000	-1.613108000
7	-6.998874000	3.747787000	-0.117446000
1	-6.939511000	3.365690000	0.815553000
1	-7.830301000	3.564522000	-0.656782000
7	4.920523000	-4.828760000	-1.508722000
1	5.392033000	-5.671764000	-1.220081000
1	5.468313000	-4.095988000	-1.937490000
8	-7.808332000	-2.899166000	2.080812000
6	-8.115252000	-3.570795000	1.071929000
1	-7.360496000	-3.878267000	0.340790000
8	7.665390000	-1.953151000	2.282170000
6	8.179561000	-2.507208000	1.287451000
1	9.137165000	-2.169581000	0.877142000
7	7.637684000	-3.546036000	0.624447000
1	8.103550000	-3.960864000	-0.166985000
1	6.757281000	-3.940158000	0.922975000
7	-9.362270000	-3.977210000	0.777530000
1	-10.130722000	-3.743695000	1.389359000
1	-9.555956000	-4.519698000	-0.049140000

Xanthione

3. L_b

6	2.204414000	0.067902000	-0.242010000
6	3.444182000	0.754246000	-0.132046000
6	3.398718000	2.177911000	-0.059554000
6	2.198433000	2.846936000	-0.107479000
6	0.945808000	2.186313000	-0.222871000
6	1.025078000	0.769762000	-0.284572000
6	-0.314731000	2.866465000	-0.269865000
6	-1.485830000	2.040420000	-0.330851000
6	-1.389489000	0.618174000	-0.378339000
6	-2.817084000	2.552997000	-0.343867000
6	-3.926732000	1.746185000	-0.384516000
6	-2.470775000	-0.219931000	-0.413574000
6	-3.792820000	0.323508000	-0.413074000
1	-2.927438000	3.628398000	-0.320579000
1	-2.297186000	-1.288972000	-0.452690000
1	-4.903281000	2.211322000	-0.409118000
1	2.160122000	-1.009545000	-0.281479000
1	2.181093000	3.927377000	-0.057142000
1	4.309421000	2.756554000	0.018792000
8	-0.136159000	-0.004632000	-0.399030000
16	-0.440516000	4.650552000	-0.225664000
7	4.630232000	0.070969000	-0.103573000
7	-4.882355000	-0.495460000	-0.441912000
6	4.655186000	-1.394385000	-0.183620000
1	3.961535000	-1.726658000	-0.960140000
1	5.655817000	-1.679985000	-0.514901000
6	4.336953000	-2.092406000	1.143256000
1	3.400440000	-1.723771000	1.569946000
1	5.142596000	-1.937233000	1.863441000
1	4.226664000	-3.169189000	0.978290000

Xanthione

6	5.904765000	0.770797000	0.116779000
1	6.695208000	0.118658000	-0.260038000
1	5.927563000	1.686482000	-0.477824000
6	6.168256000	1.104229000	1.587151000
1	6.302319000	0.192049000	2.171306000
1	5.344608000	1.688393000	2.006668000
1	7.082585000	1.699787000	1.670975000
6	-4.736189000	-1.960370000	-0.419114000
1	-5.655255000	-2.376060000	-0.836958000
1	-3.914948000	-2.256600000	-1.072748000
6	-4.491244000	-2.526305000	0.983315000
1	-5.356182000	-2.379685000	1.634564000
1	-3.616443000	-2.056374000	1.440644000
1	-4.287298000	-3.597587000	0.902680000
6	-6.247100000	0.053504000	-0.379603000
1	-6.317306000	0.928642000	-1.028641000
1	-6.914041000	-0.709995000	-0.784288000
6	-6.686045000	0.431576000	1.038717000
1	-5.988966000	1.147036000	1.482902000
1	-6.756916000	-0.449269000	1.679760000
1	-7.672366000	0.903595000	0.996371000
8	6.024741000	4.057814000	-0.162946000
6	6.954674000	4.557937000	0.506283000
1	6.780083000	5.399874000	1.183668000
8	-1.986395000	-3.426380000	-0.905940000
6	-0.799439000	-3.719734000	-1.202824000
1	-0.532907000	-4.749830000	-1.466512000
7	8.229397000	4.135008000	0.472575000
1	8.942939000	4.575998000	1.030941000
1	8.495711000	3.365192000	-0.124388000
7	0.221369000	-2.860947000	-1.231323000
1	0.067036000	-1.885605000	-0.988936000

Xanthione

1	1.166840000	-3.173155000	-1.470786000
8	2.867804000	-3.614636000	-1.791719000
6	3.504033000	-4.653149000	-1.487965000
1	3.000042000	-5.528079000	-1.070430000
8	-6.718488000	3.248660000	-0.863822000
6	-7.760834000	3.695314000	-0.336845000
1	-7.741138000	4.588326000	0.295521000
7	-8.979662000	3.150092000	-0.480770000
1	-9.106201000	2.325706000	-1.050332000
1	-9.789201000	3.553110000	-0.036152000
7	4.827641000	-4.784915000	-1.636102000
1	5.298218000	-5.643053000	-1.394218000
1	5.374159000	-4.036670000	-2.038982000
8	-7.620682000	-2.548335000	2.422053000
6	-8.084946000	-3.262485000	1.506501000
1	-7.456907000	-3.608527000	0.678946000
8	7.575489000	-1.908481000	2.086478000
6	8.096319000	-2.503617000	1.119468000
1	9.060816000	-2.188142000	0.707844000
7	7.553877000	-3.562252000	0.488572000
1	8.026327000	-4.008981000	-0.281347000
1	6.672164000	-3.946152000	0.796491000
7	-9.362584000	-3.672776000	1.435089000
1	-10.022280000	-3.406400000	2.151535000
1	-9.687056000	-4.250682000	0.676214000

Xanthione

VIII. OPTIMISED GEOMETRIES - FORMIC ACID

A. Implicit

1. S_0

6	2.308115000	-0.785951000	-0.357239000
6	3.581978000	-0.205402000	-0.292908000
6	3.639085000	1.206057000	-0.111291000
6	2.502350000	1.952063000	-0.029982000
6	1.214899000	1.387402000	-0.105218000
6	1.175869000	-0.003222000	-0.271676000
6	0.000016000	2.152612000	-0.016833000
6	-1.214870000	1.387357000	-0.104931000
6	-1.175826000	-0.003265000	-0.271495000
6	-2.502311000	1.951960000	-0.029362000
6	-3.639055000	1.205921000	-0.110551000
6	-2.308049000	-0.786016000	-0.356957000
6	-3.581909000	-0.205470000	-0.292468000
1	-2.571511000	3.021993000	0.104461000
1	-2.166189000	-1.848069000	-0.470832000
1	-4.590431000	1.706364000	-0.034109000
1	2.166215000	-1.848008000	-0.471001000
1	2.571514000	3.022122000	0.103646000
1	4.590459000	1.706561000	-0.035278000
8	0.000038000	-0.676460000	-0.351654000
16	-0.000003000	3.832481000	0.176742000
7	4.712447000	-0.948331000	-0.411819000
7	-4.712374000	-0.948407000	-0.411368000
6	4.642321000	-2.396968000	-0.561904000
1	3.860126000	-2.640241000	-1.281595000
1	5.578796000	-2.717634000	-1.014536000
6	4.417058000	-3.153749000	0.741191000

Xanthione

1	3.505474000	-2.826051000	1.240370000
1	5.250456000	-3.007553000	1.427274000
1	4.327772000	-4.221899000	0.539323000
6	6.034479000	-0.346046000	-0.273886000
1	6.740556000	-1.017507000	-0.758836000
1	6.065551000	0.583926000	-0.841362000
6	6.465502000	-0.102409000	1.166719000
1	6.536589000	-1.039200000	1.718160000
1	5.761338000	0.545280000	1.688669000
1	7.444910000	0.377364000	1.185171000
6	-4.642334000	-2.396948000	-0.562340000
1	-5.578759000	-2.717242000	-1.015352000
1	-3.860019000	-2.639881000	-1.282024000
6	-4.417345000	-3.154504000	0.740338000
1	-5.250691000	-3.008423000	1.426507000
1	-3.505635000	-2.827352000	1.239646000
1	-4.328382000	-4.222580000	0.537907000
6	-6.034325000	-0.345867000	-0.273922000
1	-6.065155000	0.583889000	-0.841805000
1	-6.740436000	-1.017413000	-0.758721000
6	-6.465626000	-0.101731000	1.166505000
1	-5.761431000	0.545871000	1.688521000
1	-6.537086000	-1.038409000	1.718106000
1	-7.444927000	0.378275000	1.184618000

2. L_a

6	2.322797000	-0.779047000	-0.359760000
6	3.602569000	-0.195098000	-0.306559000
6	3.658239000	1.202427000	-0.141357000
6	2.507769000	1.956018000	-0.058822000

Xanthione

6	1.229161000	1.387255000	-0.120165000
6	1.189530000	-0.007076000	-0.274711000
6	-0.000023000	2.111451000	-0.026070000
6	-1.229170000	1.387185000	-0.120080000
6	-1.189474000	-0.007152000	-0.274600000
6	-2.507809000	1.955868000	-0.058687000
6	-3.658241000	1.202202000	-0.141107000
6	-2.322681000	-0.779202000	-0.359561000
6	-3.602504000	-0.195328000	-0.306239000
1	-2.601228000	3.028614000	0.061475000
1	-2.186275000	-1.843348000	-0.466372000
1	-4.606827000	1.710326000	-0.077340000
1	2.186432000	-1.843210000	-0.466484000
1	2.601125000	3.028765000	0.061371000
1	4.606793000	1.710617000	-0.077681000
8	0.000056000	-0.684286000	-0.338979000
16	-0.000071000	3.811258000	0.250700000
7	4.737685000	-0.955166000	-0.432892000
7	-4.737553000	-0.955481000	-0.432335000
6	4.659134000	-2.403776000	-0.535236000
1	3.876678000	-2.669732000	-1.247730000
1	5.594215000	-2.745244000	-0.977104000
6	4.428601000	-3.125479000	0.788177000
1	3.519022000	-2.777578000	1.277570000
1	5.262302000	-2.965520000	1.471098000
1	4.331890000	-4.198709000	0.617091000
6	6.055435000	-0.353688000	-0.300608000
1	6.764821000	-1.029095000	-0.776925000
1	6.086294000	0.570725000	-0.879309000
6	6.493075000	-0.086086000	1.135329000
1	6.578819000	-1.015103000	1.698084000
1	5.782935000	0.558647000	1.652835000

Xanthione

1	7.466629000	0.406451000	1.144049000
6	-4.658939000	-2.404117000	-0.534318000
1	-5.594261000	-2.745768000	-0.975509000
1	-3.876865000	-2.670310000	-1.247167000
6	-4.427728000	-3.125299000	0.789257000
1	-5.260979000	-2.964757000	1.472600000
1	-3.517769000	-2.777440000	1.277963000
1	-4.331420000	-4.198633000	0.618605000
6	-6.055382000	-0.353844000	-0.301499000
1	-6.085550000	0.570435000	-0.880441000
1	-6.764379000	-1.029293000	-0.778331000
6	-6.494300000	-0.085977000	1.133990000
1	-5.784550000	0.558722000	1.652068000
1	-6.580630000	-1.014947000	1.696751000
1	-7.467822000	0.406643000	1.141823000

3. *L_b*

6	2.322955000	-0.777368000	-0.372799000
6	3.601144000	-0.191448000	-0.296948000
6	3.647243000	1.208152000	-0.111516000
6	2.497043000	1.954585000	-0.031491000
6	1.214531000	1.388258000	-0.115552000
6	1.192647000	-0.003327000	-0.290325000
6	-0.008729000	2.132394000	-0.027424000
6	-1.215901000	1.385827000	-0.110917000
6	-1.177637000	-0.033164000	-0.292285000
6	-2.517642000	1.957560000	-0.023039000
6	-3.653449000	1.206346000	-0.109850000
6	-2.299158000	-0.794953000	-0.380837000
6	-3.593174000	-0.202509000	-0.297061000

Xanthione

1	-2.577481000	3.025586000	0.118990000
1	-2.170032000	-1.856697000	-0.511499000
1	-4.606546000	1.703662000	-0.031815000
1	2.187686000	-1.839789000	-0.495874000
1	2.563834000	3.024196000	0.108663000
1	4.594154000	1.716991000	-0.027548000
8	0.005766000	-0.693199000	-0.382821000
16	-0.066345000	3.858543000	0.184355000
7	4.737752000	-0.943831000	-0.415165000
7	-4.708471000	-0.955872000	-0.400176000
6	4.666655000	-2.391515000	-0.544441000
1	3.893853000	-2.646919000	-1.270844000
1	5.608901000	-2.721241000	-0.979231000
6	4.421794000	-3.133881000	0.764861000
1	3.503474000	-2.798446000	1.246335000
1	5.245177000	-2.980248000	1.461429000
1	4.333678000	-4.204520000	0.574755000
6	6.053672000	-0.339329000	-0.268210000
1	6.767410000	-1.006188000	-0.749474000
1	6.083247000	0.593125000	-0.833152000
6	6.479144000	-0.092005000	1.174904000
1	6.561279000	-1.028764000	1.725050000
1	5.764553000	0.545355000	1.695246000
1	7.452265000	0.400690000	1.196914000
6	-4.638152000	-2.406633000	-0.565831000
1	-5.579009000	-2.719484000	-1.012024000
1	-3.859489000	-2.641488000	-1.289571000
6	-4.405174000	-3.163831000	0.736696000
1	-5.229977000	-3.014615000	1.431399000
1	-3.484300000	-2.844705000	1.223369000
1	-4.328150000	-4.230370000	0.524241000
6	-6.037890000	-0.364736000	-0.257670000

Xanthione

1	-6.072567000	0.564026000	-0.825439000
1	-6.739015000	-1.045811000	-0.733702000
6	-6.451351000	-0.123308000	1.189828000
1	-5.746116000	0.530153000	1.701804000
1	-6.515008000	-1.059200000	1.742385000
1	-7.432821000	0.350842000	1.209320000

B. S=2

1. S_0

6	-1.523316000	1.183624000	-0.319783000
6	-2.896871000	0.874451000	-0.280448000
6	-3.259167000	-0.503755000	-0.177438000
6	-2.303886000	-1.485192000	-0.129982000
6	-0.917321000	-1.199368000	-0.173868000
6	-0.588262000	0.168964000	-0.270172000
6	0.111824000	-2.200562000	-0.117955000
6	1.470744000	-1.727768000	-0.164566000
6	1.752755000	-0.350315000	-0.262527000
6	2.603845000	-2.573192000	-0.110567000
6	3.886937000	-2.090018000	-0.149354000
6	3.030531000	0.171185000	-0.307741000
6	4.144446000	-0.688262000	-0.257217000
1	2.430833000	-3.638221000	-0.030843000
1	3.131445000	1.242994000	-0.376605000
1	4.706353000	-2.790614000	-0.091529000
1	-1.159085000	2.197403000	-0.377335000
1	-2.618602000	-2.516672000	-0.050025000
1	-4.296848000	-0.796368000	-0.133296000
8	0.731677000	0.579701000	-0.313552000

Xanthione

16	-0.263132000	-3.900684000	-0.000385000
7	-3.848690000	1.854820000	-0.346817000
7	5.420137000	-0.196398000	-0.319728000
6	-3.463844000	3.271463000	-0.403729000
1	-2.644513000	3.390418000	-1.119264000
1	-4.314638000	3.820106000	-0.812942000
6	-3.067752000	3.861323000	0.951136000
1	-2.234736000	3.309691000	1.393917000
1	-3.908041000	3.833471000	1.649537000
1	-2.755979000	4.902852000	0.830220000
6	-5.280352000	1.533809000	-0.249100000
1	-5.825678000	2.367302000	-0.697023000
1	-5.496314000	0.653330000	-0.859170000
6	-5.763716000	1.305051000	1.183550000
1	-5.620876000	2.200875000	1.793483000
1	-5.222535000	0.479545000	1.653117000
1	-6.828955000	1.055704000	1.185837000
6	5.662569000	1.252358000	-0.375877000
1	6.669757000	1.393885000	-0.772848000
1	4.976720000	1.702866000	-1.099535000
6	5.533681000	1.955160000	0.976698000
1	6.273719000	1.576097000	1.686147000
1	4.539507000	1.808175000	1.405983000
1	5.692735000	3.030647000	0.856140000
6	6.587078000	-1.083959000	-0.226893000
1	6.417924000	-1.966474000	-0.850413000
1	7.430020000	-0.552155000	-0.672887000
6	6.934335000	-1.498902000	1.203754000
1	6.094037000	-2.008428000	1.683440000
1	7.193178000	-0.627119000	1.810166000
1	7.790440000	-2.180088000	1.201954000
8	-4.968804000	-3.418617000	0.261145000

Xanthione

6	-6.123728000	-3.024620000	0.195048000
1	-6.991753000	-3.640139000	0.443176000
8	2.528625000	3.461605000	-0.459938000
6	1.449130000	3.447629000	0.123063000
1	1.259474000	2.977820000	1.084221000
8	-6.411087000	-1.758098000	-0.203330000
1	-7.364281000	-1.544938000	-0.217306000
8	0.315892000	4.020312000	-0.353247000
1	0.438291000	4.446172000	-1.229152000

2. *L_a*

6	1.545792000	1.206715000	0.338618000
6	2.924806000	0.898156000	0.311875000
6	3.286017000	-0.466943000	0.225815000
6	2.322800000	-1.459953000	0.181031000
6	0.943896000	-1.170592000	0.211016000
6	0.609133000	0.197760000	0.291978000
6	-0.103509000	-2.140379000	0.153038000
6	-1.467750000	-1.717595000	0.188869000
6	-1.756433000	-0.338039000	0.273614000
6	-2.587784000	-2.570222000	0.135506000
6	-3.887066000	-2.088733000	0.163406000
6	-3.036577000	0.167403000	0.307275000
6	-4.150201000	-0.701769000	0.258068000
1	-2.432821000	-3.641817000	0.066218000
1	-3.148847000	1.239111000	0.368201000
1	-4.698809000	-2.799023000	0.107768000
1	1.183896000	2.222483000	0.385524000
1	2.662433000	-2.488012000	0.114928000
1	4.323201000	-0.764197000	0.188904000

Xanthione

8	-0.727542000	0.604418000	0.320170000
16	0.295155000	-3.872752000	0.006013000
7	3.878543000	1.896595000	0.382009000
7	-5.437980000	-0.202728000	0.316922000
6	3.484930000	3.307591000	0.406758000
1	2.658285000	3.438710000	1.112861000
1	4.328426000	3.871576000	0.812436000
6	3.094655000	3.875246000	-0.960449000
1	2.270118000	3.308493000	-1.400442000
1	3.940753000	3.842167000	-1.651996000
1	2.772955000	4.916362000	-0.860419000
6	5.305709000	1.576913000	0.287363000
1	5.853819000	2.417549000	0.720208000
1	5.523617000	0.706854000	0.914272000
6	5.796596000	1.320268000	-1.139527000
1	5.657645000	2.205795000	-1.765556000
1	5.254876000	0.487901000	-1.595815000
1	6.861942000	1.069126000	-1.134378000
6	-5.680066000	1.242889000	0.346018000
1	-6.690461000	1.392276000	0.734067000
1	-5.000463000	1.709134000	1.066571000
6	-5.543815000	1.929876000	-1.015085000
1	-6.280087000	1.541227000	-1.723646000
1	-4.547426000	1.774497000	-1.436783000
1	-5.701875000	3.007638000	-0.911502000
6	-6.595027000	-1.094577000	0.213583000
1	-6.430065000	-1.972048000	0.847391000
1	-7.450178000	-0.565776000	0.641417000
6	-6.922712000	-1.530764000	-1.216814000
1	-6.068749000	-2.031083000	-1.681838000
1	-7.187881000	-0.668704000	-1.834696000
1	-7.768224000	-2.225563000	-1.218611000

Xanthione

8	4.862123000	-3.544766000	-0.158814000
6	6.012927000	-3.151034000	-0.279609000
1	6.835935000	-3.780008000	-0.627935000
8	-2.536023000	3.486516000	0.441021000
6	-1.453501000	3.441077000	-0.134925000
1	-1.258214000	2.920214000	-1.067662000
8	6.353356000	-1.871275000	0.020319000
1	7.296187000	-1.659343000	-0.123005000
8	-0.323440000	4.040464000	0.316186000
1	-0.451106000	4.508797000	1.169209000

3. L_b

6	-1.512986000	1.166357000	-0.335225000
6	-2.901772000	0.847284000	-0.270808000
6	-3.258188000	-0.531104000	-0.154636000
6	-2.296448000	-1.508887000	-0.105210000
6	-0.899635000	-1.216711000	-0.166584000
6	-0.577441000	0.171393000	-0.283445000
6	0.133188000	-2.210700000	-0.111874000
6	1.486343000	-1.735469000	-0.162191000
6	1.775331000	-0.352571000	-0.279005000
6	2.628362000	-2.574560000	-0.093831000
6	3.915996000	-2.087028000	-0.136389000
6	3.044857000	0.167105000	-0.327788000
6	4.170291000	-0.691875000	-0.260095000
1	2.456442000	-3.638439000	0.000550000
1	3.145615000	1.238183000	-0.413665000
1	4.735654000	-2.787358000	-0.068269000
1	-1.160500000	2.183311000	-0.415694000
1	-2.595006000	-2.543078000	-0.013282000

Xanthione

1	-4.294190000	-0.829503000	-0.101997000
8	0.746439000	0.590646000	-0.348572000
16	-0.273617000	-3.945508000	0.025243000
7	-3.844667000	1.830420000	-0.320709000
7	5.444688000	-0.185300000	-0.322110000
6	-3.463233000	3.250649000	-0.387187000
1	-2.657766000	3.369889000	-1.116284000
1	-4.324989000	3.794744000	-0.775883000
6	-3.042613000	3.833216000	0.965333000
1	-2.190967000	3.290760000	1.382177000
1	-3.865163000	3.792626000	1.682945000
1	-2.748880000	4.878579000	0.836832000
6	-5.278200000	1.514493000	-0.207111000
1	-5.825342000	2.348555000	-0.649006000
1	-5.498043000	0.628552000	-0.806060000
6	-5.734815000	1.292415000	1.237304000
1	-5.595294000	2.193524000	1.839056000
1	-5.179441000	0.474379000	1.702257000
1	-6.796663000	1.032158000	1.249271000
6	5.678083000	1.263904000	-0.384903000
1	6.688408000	1.409155000	-0.772001000
1	4.995642000	1.706861000	-1.116190000
6	5.531668000	1.973370000	0.963831000
1	6.263391000	1.599361000	1.684225000
1	4.531969000	1.827691000	1.380162000
1	5.691555000	3.047979000	0.837172000
6	6.615724000	-1.063699000	-0.216670000
1	6.450958000	-1.953525000	-0.831054000
1	7.458576000	-0.531682000	-0.662316000
6	6.956850000	-1.464485000	1.221017000
1	6.113519000	-1.967927000	1.701226000
1	7.215932000	-0.587447000	1.819420000

Xanthione

1	7.811781000	-2.146989000	1.226766000
8	-5.061852000	-3.318914000	0.252702000
6	-6.214005000	-2.933667000	0.121827000
1	-7.090436000	-3.564926000	0.285714000
8	2.519414000	3.460631000	-0.469388000
6	1.433970000	3.416795000	0.100441000
1	1.244718000	2.942446000	1.059126000
8	-6.486302000	-1.654241000	-0.247283000
1	-7.438538000	-1.447768000	-0.318769000
8	0.291274000	3.959820000	-0.390899000
1	0.415023000	4.391279000	-1.263877000

C. S=4

1. S_0

6	-2.083606000	0.483039000	-0.285775000
6	-3.290462000	-0.240132000	-0.206807000
6	-3.204760000	-1.666592000	-0.232405000
6	-1.996577000	-2.302025000	-0.345538000
6	-0.771840000	-1.595892000	-0.432378000
6	-0.882734000	-0.189471000	-0.392257000
6	0.511424000	-2.228958000	-0.546817000
6	1.653439000	-1.352612000	-0.615928000
6	1.496276000	0.046833000	-0.559737000
6	2.986530000	-1.805686000	-0.727582000
6	4.059368000	-0.944275000	-0.760897000
6	2.548171000	0.941910000	-0.596389000
6	3.871321000	0.473180000	-0.700486000
1	3.159987000	-2.872850000	-0.777566000
1	2.309452000	1.992373000	-0.533572000

Xanthione

1	5.049933000	-1.364401000	-0.858882000
1	-2.049444000	1.559379000	-0.243839000
1	-1.975218000	-3.383030000	-0.365537000
1	-4.096202000	-2.270648000	-0.163435000
8	0.242104000	0.613084000	-0.450027000
16	0.687676000	-3.962222000	-0.596931000
7	-4.499030000	0.392543000	-0.117415000
7	4.929152000	1.337431000	-0.746794000
6	-4.582739000	1.858580000	-0.076312000
1	-3.902550000	2.274132000	-0.825458000
1	-5.592783000	2.130089000	-0.390525000
6	-4.288812000	2.463306000	1.298532000
1	-3.273591000	2.231047000	1.629712000
1	-4.991098000	2.086239000	2.046394000
1	-4.386482000	3.552469000	1.258462000
6	-5.746502000	-0.370430000	0.042352000
1	-6.562081000	0.282189000	-0.275846000
1	-5.741433000	-1.222488000	-0.641726000
6	-5.992547000	-0.852427000	1.472485000
1	-6.076510000	-0.008184000	2.161725000
1	-5.178454000	-1.496323000	1.815002000
1	-6.921638000	-1.428058000	1.520846000
6	4.718235000	2.785788000	-0.604103000
1	5.601111000	3.280213000	-1.013889000
1	3.873398000	3.088000000	-1.229687000
6	4.490702000	3.236481000	0.839776000
1	5.371061000	3.035966000	1.455445000
1	3.635809000	2.721220000	1.284250000
1	4.287387000	4.310585000	0.868989000
6	6.314152000	0.851219000	-0.816132000
1	6.367456000	0.018317000	-1.522098000
1	6.914101000	1.654893000	-1.248087000

Xanthione

6	6.894436000	0.443842000	0.539491000
1	6.284975000	-0.323598000	1.023960000
1	6.950608000	1.302529000	1.213325000
1	7.904903000	0.044650000	0.411532000
8	-3.887832000	-5.004980000	-0.144190000
6	-5.106110000	-4.994860000	-0.048657000
1	-5.709174000	-5.887889000	0.132382000
8	1.077709000	3.798537000	0.069924000
6	0.442543000	3.320219000	1.012948000
1	0.900345000	2.765508000	1.831452000
8	-5.808900000	-3.838114000	-0.165459000
1	-6.775693000	-3.937772000	-0.065584000
8	-0.884010000	3.413148000	1.174010000
1	-1.376524000	3.860584000	0.417499000
8	-2.226087000	4.360501000	-0.883716000
6	-3.047854000	5.277035000	-0.948747000
1	-3.512350000	5.579316000	-1.887346000
8	5.475960000	-3.774979000	0.406029000
6	5.450108000	-3.405120000	1.577578000
1	5.982896000	-3.891325000	2.393130000
8	4.758280000	-2.334461000	2.031539000
1	4.255915000	-1.871719000	1.317844000
8	-3.413204000	5.944636000	0.153386000
1	-4.066911000	6.657641000	0.012217000

2. L_a

6	1.993389000	0.593646000	0.350480000
6	3.239023000	-0.071309000	0.281105000
6	3.225427000	-1.485896000	0.333816000
6	2.039657000	-2.186339000	0.461262000

Xanthione

6	0.790385000	-1.538497000	0.537604000
6	0.827356000	-0.129302000	0.471747000
6	-0.471965000	-2.196352000	0.652540000
6	-1.667557000	-1.422622000	0.748912000
6	-1.586157000	-0.013697000	0.657092000
6	-2.962825000	-1.947448000	0.907778000
6	-4.095049000	-1.136610000	0.928304000
6	-2.686533000	0.812103000	0.683108000
6	-3.987571000	0.271663000	0.815620000
1	-3.095712000	-3.019738000	0.987253000
1	-2.513534000	1.873242000	0.587286000
1	-5.059048000	-1.605890000	1.065014000
1	1.909406000	1.666951000	0.288966000
1	2.095123000	-3.268914000	0.501202000
1	4.143829000	-2.049900000	0.276052000
8	-0.353592000	0.617389000	0.510332000
16	-0.564043000	-3.975857000	0.572677000
7	4.421985000	0.634112000	0.178342000
7	-5.094493000	1.092412000	0.843738000
6	4.422272000	2.096280000	0.084661000
1	3.725262000	2.503350000	0.824243000
1	5.417712000	2.436785000	0.380386000
6	4.085844000	2.640872000	-1.306627000
1	3.081930000	2.342648000	-1.619538000
1	4.801064000	2.275339000	-2.048291000
1	4.124851000	3.734625000	-1.305333000
6	5.700246000	-0.068404000	0.027965000
1	6.487915000	0.630559000	0.319669000
1	5.742305000	-0.900161000	0.737523000
6	5.966176000	-0.585296000	-1.387875000
1	6.014472000	0.240316000	-2.102949000
1	5.177925000	-1.271320000	-1.708226000

Xanthione

1	6.918160000	-1.124150000	-1.422032000
6	-4.959564000	2.538710000	0.635533000
1	-5.864152000	3.005949000	1.031393000
1	-4.128152000	2.914202000	1.239794000
6	-4.764792000	2.942508000	-0.827751000
1	-5.637346000	2.670362000	-1.427301000
1	-3.887170000	2.453010000	-1.257241000
1	-4.617564000	4.023687000	-0.904630000
6	-6.445904000	0.529648000	0.917313000
1	-6.464669000	-0.261379000	1.673676000
1	-7.104231000	1.319331000	1.286649000
6	-6.976670000	0.000158000	-0.417914000
1	-6.311979000	-0.757071000	-0.842626000
1	-7.072466000	0.809735000	-1.146177000
1	-7.963231000	-0.452602000	-0.279816000
8	3.984509000	-4.845103000	0.273377000
6	5.195364000	-4.786371000	0.117396000
1	5.816826000	-5.647985000	-0.138507000
8	-1.363603000	3.719095000	-0.125481000
6	-0.711791000	3.203104000	-1.036570000
1	-1.144262000	2.556621000	-1.799334000
8	5.865655000	-3.613711000	0.256605000
1	6.828245000	-3.676526000	0.102310000
8	0.604479000	3.364577000	-1.228176000
1	1.076520000	3.890767000	-0.511250000
8	1.886956000	4.524249000	0.761452000
6	2.679674000	5.467257000	0.785593000
1	3.110981000	5.844615000	1.713027000
8	-3.874414000	-4.291469000	-1.271251000
6	-4.156108000	-3.507417000	-2.174668000
1	-4.391248000	-3.803491000	-3.196397000
8	-4.218352000	-2.164163000	-2.045392000

Xanthione

1	-4.011555000	-1.845451000	-1.130129000
8	3.054191000	6.074835000	-0.348330000
1	3.685459000	6.812604000	-0.235685000

3. L_b

6	-2.059296000	0.500403000	-0.315471000
6	-3.286824000	-0.222730000	-0.214472000
6	-3.217277000	-1.649608000	-0.235431000
6	-2.014264000	-2.299566000	-0.350783000
6	-0.774124000	-1.598537000	-0.452064000
6	-0.874065000	-0.170085000	-0.422291000
6	0.503493000	-2.235506000	-0.571863000
6	1.655237000	-1.385249000	-0.642619000
6	1.524317000	0.022394000	-0.601464000
6	2.985545000	-1.851438000	-0.743479000
6	4.076230000	-0.997124000	-0.785597000
6	2.582942000	0.899103000	-0.644986000
6	3.908334000	0.413480000	-0.739992000
1	3.147045000	-2.920912000	-0.783045000
1	2.359376000	1.953920000	-0.593405000
1	5.061329000	-1.434024000	-0.869572000
1	-2.024932000	1.577310000	-0.289318000
1	-1.993586000	-3.379267000	-0.364506000
1	-4.114649000	-2.245114000	-0.161749000
8	0.264521000	0.619017000	-0.499253000
16	0.615109000	-4.019185000	-0.615613000
7	-4.476114000	0.431182000	-0.102944000
7	4.977363000	1.276957000	-0.789461000
6	-4.544658000	1.901251000	-0.059520000
1	-3.870829000	2.311007000	-0.815637000

Xanthione

1	-5.557433000	2.179787000	-0.353961000
6	-4.221850000	2.489867000	1.317870000
1	-3.199641000	2.255967000	1.624262000
1	-4.910167000	2.109738000	2.076048000
1	-4.321520000	3.578524000	1.281767000
6	-5.736904000	-0.310944000	0.070449000
1	-6.543952000	0.356642000	-0.233845000
1	-5.747861000	-1.163902000	-0.611142000
6	-5.962455000	-0.788270000	1.507638000
1	-6.029659000	0.055432000	2.198259000
1	-5.151530000	-1.442734000	1.835456000
1	-6.897483000	-1.352318000	1.562230000
6	4.779562000	2.724980000	-0.649519000
1	5.669732000	3.211099000	-1.054337000
1	3.941214000	3.036116000	-1.279850000
6	4.547448000	3.182909000	0.792607000
1	5.420797000	2.972302000	1.414921000
1	3.682660000	2.679242000	1.231128000
1	4.357755000	4.259759000	0.817864000
6	6.354006000	0.772828000	-0.832799000
1	6.406626000	-0.063408000	-1.535824000
1	6.975414000	1.566424000	-1.253546000
6	6.903613000	0.355441000	0.534140000
1	6.258808000	-0.385267000	1.014203000
1	6.980957000	1.216817000	1.202488000
1	7.900903000	-0.080411000	0.422795000
8	-4.025081000	-4.913637000	-0.163620000
6	-5.244986000	-4.881352000	-0.103201000
1	-5.872717000	-5.767234000	0.017457000
8	1.120669000	3.771696000	0.076306000
6	0.482169000	3.234838000	0.984248000
1	0.934031000	2.622835000	1.763844000

Xanthione

8	-5.918939000	-3.703770000	-0.185082000
1	-6.890227000	-3.786942000	-0.119266000
8	-0.845120000	3.327823000	1.150441000
1	-1.332235000	3.824938000	0.422895000
8	-2.185525000	4.378158000	-0.859020000
6	-2.972524000	5.326201000	-0.903170000
1	-3.401128000	5.686897000	-1.838320000
8	5.482220000	-3.774839000	0.606743000
6	5.341485000	-3.330142000	1.743879000
1	5.816365000	-3.743142000	2.632643000
8	4.579859000	-2.259776000	2.063695000
1	4.125434000	-1.863158000	1.278909000
8	-3.343968000	5.956213000	0.218596000
1	-3.969399000	6.697129000	0.093693000

D. S=6

1. S_0

6	-1.914583000	1.059580000	-0.212891000
6	-2.835328000	2.127518000	-0.245569000
6	-2.304181000	3.443263000	-0.413248000
6	-0.955656000	3.650876000	-0.551174000
6	-0.015572000	2.593774000	-0.527023000
6	-0.565580000	1.310199000	-0.346868000
6	1.404940000	2.779384000	-0.668212000
6	2.213324000	1.588822000	-0.647945000
6	1.635727000	0.318357000	-0.456002000
6	3.616317000	1.588344000	-0.822452000
6	4.361811000	0.437871000	-0.784498000
6	2.347497000	-0.860791000	-0.417028000

Xanthione

6	3.751357000	-0.832716000	-0.552796000
1	4.103065000	2.539094000	-0.992476000
1	1.803899000	-1.784652000	-0.279034000
1	5.424835000	0.501923000	-0.960778000
1	-2.233662000	0.038629000	-0.076174000
1	-0.575470000	4.655484000	-0.680468000
1	-2.964518000	4.297137000	-0.426882000
8	0.261440000	0.193907000	-0.277910000
16	2.109406000	4.359798000	-0.855017000
7	-4.176785000	1.903401000	-0.133107000
7	4.493996000	-1.973726000	-0.454679000
6	-5.147694000	3.008068000	-0.167911000
1	-4.854066000	3.717778000	-0.946008000
1	-6.104785000	2.584598000	-0.477520000
6	-5.310484000	3.720156000	1.175593000
1	-4.357243000	4.119773000	1.533002000
1	-5.697038000	3.034253000	1.934229000
1	-6.014778000	4.551739000	1.078454000
6	-4.699917000	0.551236000	0.107445000
1	-5.757819000	0.578659000	-0.151465000
1	-4.223523000	-0.151050000	-0.584771000
6	-4.542307000	0.064643000	1.549093000
1	-5.040302000	0.745728000	2.244021000
1	-3.491899000	-0.014249000	1.840267000
1	-5.000428000	-0.922736000	1.653106000
6	3.845469000	-3.287731000	-0.327312000
1	4.592333000	-3.974250000	0.075996000
1	3.040682000	-3.227312000	0.410680000
6	3.297975000	-3.828630000	-1.648097000
1	4.101812000	-3.974919000	-2.374418000
1	2.563880000	-3.143664000	-2.079903000
1	2.802022000	-4.788729000	-1.480821000

Xanthione

6	5.960355000	-1.930719000	-0.520273000
1	6.309445000	-1.054403000	0.029422000
1	6.332643000	-2.801706000	0.024074000
6	6.525550000	-1.924545000	-1.940345000
1	6.119949000	-1.093604000	-2.524403000
1	6.285333000	-2.854499000	-2.462217000
1	7.613224000	-1.816779000	-1.904839000
8	-3.453081000	-4.312811000	1.577418000
6	-4.230342000	-3.795328000	0.771533000
1	-5.282684000	-3.608180000	1.004339000
8	0.402810000	-3.473957000	0.163436000
6	-0.664867000	-3.141837000	0.688814000
1	-1.425303000	-3.830914000	1.055422000
8	-3.841225000	-3.416293000	-0.451274000
1	-4.574929000	-3.012987000	-1.014467000
8	-1.046944000	-1.864145000	0.874859000
1	-0.433211000	-1.179032000	0.490377000
8	-8.063743000	1.041500000	-0.313224000
6	-8.831123000	0.732194000	0.582617000
1	-9.538923000	1.404341000	1.066726000
8	6.552988000	1.132641000	2.023774000
6	6.779741000	1.908290000	1.085581000
1	7.577561000	1.757367000	0.362526000
8	6.065286000	3.012149000	0.845038000
1	5.326277000	3.157662000	1.475906000
8	-8.840478000	-0.554597000	1.050617000
1	-9.478486000	-0.757412000	1.761928000
8	8.843509000	-0.194448000	0.181531000
6	8.963285000	-0.957379000	1.147981000
1	9.692359000	-1.765615000	1.189280000
8	-5.760842000	-2.294933000	-1.832659000
6	-6.735380000	-1.814502000	-1.237433000

Xanthione

1	-7.466943000	-1.184053000	-1.742458000
8	-6.924495000	-2.035351000	0.059814000
1	-7.703212000	-1.554995000	0.475190000
8	8.218881000	-0.902779000	2.257562000
1	7.544071000	-0.150340000	2.247597000

2. L_a

6	1.912487000	-1.036242000	-0.356135000
6	2.841959000	-2.100838000	-0.415797000
6	2.319796000	-3.407379000	-0.569823000
6	0.956359000	-3.627218000	-0.667646000
6	0.016858000	-2.578385000	-0.606807000
6	0.563427000	-1.289399000	-0.443568000
6	-1.401174000	-2.733940000	-0.686786000
6	-2.246047000	-1.582389000	-0.662896000
6	-1.674149000	-0.304139000	-0.493086000
6	-3.647603000	-1.600384000	-0.801805000
6	-4.408646000	-0.444257000	-0.754652000
6	-2.398785000	0.862947000	-0.449777000
6	-3.809580000	0.822053000	-0.554681000
1	-4.153133000	-2.546613000	-0.959462000
1	-1.863716000	1.794569000	-0.328249000
1	-5.474896000	-0.524098000	-0.905503000
1	2.233181000	-0.013704000	-0.231208000
1	0.601910000	-4.644448000	-0.793834000
1	2.981641000	-4.259858000	-0.608825000
8	-0.283470000	-0.172790000	-0.340070000
16	-2.121671000	-4.363150000	-0.755011000
7	4.196392000	-1.855662000	-0.342380000
7	-4.558585000	1.974330000	-0.450629000

Xanthione

6	5.169744000	-2.950078000	-0.415022000
1	4.855495000	-3.655824000	-1.189914000
1	6.116891000	-2.521113000	-0.748022000
6	5.384374000	-3.678185000	0.913877000
1	4.443446000	-4.073938000	1.306620000
1	5.806755000	-3.000998000	1.661558000
1	6.078751000	-4.513664000	0.781571000
6	4.705162000	-0.510746000	-0.056484000
1	5.763796000	-0.520398000	-0.316147000
1	4.224609000	0.216806000	-0.720687000
6	4.548378000	-0.073897000	1.402318000
1	5.066568000	-0.767401000	2.070165000
1	3.497842000	-0.032394000	1.701150000
1	4.982466000	0.920034000	1.539967000
6	-3.914140000	3.291678000	-0.408075000
1	-4.652035000	3.995221000	-0.015903000
1	-3.085635000	3.275350000	0.306707000
6	-3.409211000	3.774770000	-1.768803000
1	-4.234918000	3.873484000	-2.478713000
1	-2.679207000	3.076443000	-2.186710000
1	-2.922173000	4.748865000	-1.666234000
6	-6.021322000	1.916651000	-0.461034000
1	-6.339085000	1.044660000	0.115933000
1	-6.386315000	2.792339000	0.082501000
6	-6.645775000	1.878470000	-1.857187000
1	-6.246502000	1.047117000	-2.445091000
1	-6.443877000	2.805075000	-2.401302000
1	-7.729282000	1.750876000	-1.778576000
8	3.455466000	4.318694000	1.420680000
6	4.321937000	3.824672000	0.695468000
1	5.350857000	3.667326000	1.031365000
8	-0.435266000	3.492031000	0.085645000

Xanthione

6	0.657775000	3.150646000	0.551015000
1	1.438549000	3.836677000	0.880690000
8	4.067073000	3.435545000	-0.559123000
1	4.866029000	3.060998000	-1.048763000
8	1.045760000	1.873338000	0.710409000
1	0.416250000	1.179381000	0.355485000
8	8.077861000	-1.105232000	-0.044247000
6	8.651495000	-0.879191000	1.007529000
1	9.193120000	-1.614191000	1.602028000
8	-6.545528000	-1.102101000	2.120140000
6	-6.775103000	-1.894273000	1.196546000
1	-7.587756000	-1.766652000	0.485601000
8	-6.047169000	-2.990729000	0.961436000
1	-5.294050000	-3.110599000	1.580909000
8	8.641943000	0.389460000	1.524354000
1	9.120165000	0.523399000	2.365222000
8	-8.916441000	0.133275000	0.311968000
6	-9.004000000	0.934562000	1.250219000
1	-9.733224000	1.743049000	1.284306000
8	6.145341000	2.373759000	-1.749970000
6	7.013154000	1.840939000	-1.044194000
1	7.789197000	1.202364000	-1.465695000
8	7.025669000	2.016201000	0.273393000
1	7.704751000	1.479884000	0.784416000
8	-8.221871000	0.925651000	2.334992000
1	-7.543548000	0.176167000	2.330740000

3. L_b

6	1.640461000	-0.921588000	0.216289000
6	2.382486000	-2.125102000	0.118931000

Xanthione

6	1.653540000	-3.342963000	0.161717000
6	0.279039000	-3.350091000	0.288816000
6	-0.476954000	-2.159955000	0.393262000
6	0.275192000	-0.964432000	0.356034000
6	-1.893547000	-2.097166000	0.555232000
6	-2.543119000	-0.827167000	0.488741000
6	-1.770218000	0.358080000	0.461502000
6	-3.947172000	-0.636783000	0.446506000
6	-4.518302000	0.615879000	0.394326000
6	-2.301928000	1.619434000	0.392579000
6	-3.714015000	1.787444000	0.378251000
1	-4.594463000	-1.504194000	0.417659000
1	-1.622252000	2.459013000	0.342082000
1	-5.595492000	0.677516000	0.342609000
1	2.122089000	0.043846000	0.200082000
1	-0.246985000	-4.297343000	0.303128000
1	2.168949000	-4.289133000	0.088214000
8	-0.368469000	0.275846000	0.503417000
16	-2.829831000	-3.522710000	1.087040000
7	3.746726000	-2.095828000	-0.033959000
7	-4.268343000	3.039486000	0.332729000
6	4.529813000	-3.334708000	-0.119707000
1	4.012785000	-4.039739000	-0.777804000
1	5.473524000	-3.086294000	-0.609288000
6	4.807822000	-3.980900000	1.239579000
1	3.878481000	-4.203100000	1.771287000
1	5.408951000	-3.318700000	1.868393000
1	5.358132000	-4.917213000	1.106565000
6	4.482132000	-0.826263000	-0.021372000
1	5.455863000	-1.026244000	-0.468299000
1	3.977606000	-0.107205000	-0.675628000
6	4.676562000	-0.224582000	1.372680000

Xanthione

1	5.200835000	-0.926016000	2.026905000
1	3.723092000	0.033596000	1.840720000
1	5.278162000	0.685524000	1.297565000
6	-3.424471000	4.239731000	0.243843000
1	-4.025663000	5.079647000	0.598138000
1	-2.581941000	4.141243000	0.933373000
6	-2.906246000	4.529029000	-1.166988000
1	-3.730794000	4.724654000	-1.856984000
1	-2.325827000	3.687249000	-1.552978000
1	-2.255110000	5.407488000	-1.149672000
6	-5.724905000	3.216194000	0.247747000
1	-6.214473000	2.516034000	0.928837000
1	-5.947021000	4.221926000	0.610744000
6	-6.285151000	3.034913000	-1.165055000
1	-6.052334000	2.040212000	-1.552929000
1	-5.873858000	3.779747000	-1.850904000
1	-7.373221000	3.143907000	-1.149248000
8	4.183702000	4.231388000	1.341061000
6	4.679387000	3.574219000	0.422919000
1	5.727556000	3.260701000	0.419895000
8	0.098297000	3.856671000	0.479416000
6	1.135294000	3.424063000	0.995024000
1	2.016897000	4.024183000	1.216383000
8	3.966948000	3.187388000	-0.642470000
1	4.494048000	2.665424000	-1.326596000
8	1.325774000	2.140030000	1.345726000
1	0.587575000	1.514222000	1.087305000
8	7.577890000	-1.766166000	-1.154669000
6	8.580742000	-1.510174000	-0.510010000
1	9.315143000	-2.239386000	-0.170036000
8	-6.202199000	-2.778799000	-0.644210000
6	-5.830452000	-3.661184000	-1.436472000

Xanthione

1	-6.424128000	-3.984487000	-2.289157000
8	-4.681734000	-4.313977000	-1.349590000
1	-4.082171000	-4.054763000	-0.578280000
8	8.849019000	-0.213019000	-0.162630000
1	9.667159000	-0.057513000	0.347613000
8	-7.710721000	0.349224000	0.309241000
6	-8.581310000	-0.246398000	-0.331341000
1	-9.571493000	0.168884000	-0.520273000
8	5.363233000	1.767161000	-2.339765000
6	6.395010000	1.196122000	-1.959860000
1	6.908272000	0.460383000	-2.578733000
8	6.917994000	1.446283000	-0.763768000
1	7.718153000	0.892104000	-0.513526000
8	-8.454890000	-1.458383000	-0.876531000
1	-7.564295000	-1.921533000	-0.752075000

IX. OPTIMISED GEOMETRIES - TOLUENE

A. Implicit

1. S_0

6	2.309759000	-0.784494000	-0.356784000
6	3.580778000	-0.203926000	-0.296448000
6	3.639265000	1.204098000	-0.117477000
6	2.501073000	1.951083000	-0.033206000
6	1.217075000	1.385914000	-0.104550000
6	1.175393000	-0.001345000	-0.269510000
6	-0.000012000	2.159903000	-0.010331000
6	-1.217092000	1.385852000	-0.104486000
6	-1.175375000	-0.001393000	-0.269363000
6	-2.501098000	1.950998000	-0.033044000

Xanthione

6	-3.639282000	1.203980000	-0.117144000
6	-2.309728000	-0.784601000	-0.356486000
6	-3.580734000	-0.204054000	-0.296025000
1	-2.564733000	3.021917000	0.099374000
1	-2.166345000	-1.846573000	-0.469301000
1	-4.590647000	1.705179000	-0.043875000
1	2.166397000	-1.846480000	-0.469530000
1	2.564724000	3.021991000	0.099292000
1	4.590624000	1.705347000	-0.044343000
8	0.000024000	-0.678232000	-0.346657000
16	-0.000052000	3.822768000	0.192374000
7	4.716128000	-0.951526000	-0.418536000
7	-4.716061000	-0.951776000	-0.417860000
6	4.643440000	-2.398517000	-0.553970000
1	3.860940000	-2.647791000	-1.272091000
1	5.579281000	-2.726898000	-1.004370000
6	4.415090000	-3.145852000	0.755009000
1	3.507122000	-2.806585000	1.252605000
1	5.248952000	-2.998804000	1.440638000
1	4.318717000	-4.215786000	0.564226000
6	6.034881000	-0.347983000	-0.286703000
1	6.741985000	-1.017827000	-0.774269000
1	6.061848000	0.581083000	-0.856734000
6	6.474449000	-0.097759000	1.150948000
1	6.556781000	-1.032664000	1.704375000
1	5.766048000	0.542302000	1.676108000
1	7.449643000	0.391355000	1.165446000
6	-4.643199000	-2.398626000	-0.553512000
1	-5.579276000	-2.727159000	-1.003342000
1	-3.861144000	-2.647747000	-1.272205000
6	-4.414066000	-3.146180000	0.755220000
1	-5.247839000	-2.999700000	1.441082000

Xanthione

1	-3.506118000	-2.806581000	1.252621000
1	-4.317273000	-4.216029000	0.564149000
6	-6.034840000	-0.348103000	-0.287184000
1	-6.061207000	0.580859000	-0.857452000
1	-6.741641000	-1.018004000	-0.775158000
6	-6.475689000	-0.097663000	1.150030000
1	-5.767958000	0.542669000	1.675753000
1	-6.558185000	-1.032506000	1.703522000
1	-7.451058000	0.391120000	1.163607000

2. *L_a*

6	2.319889000	-0.776656000	-0.364385000
6	3.598059000	-0.194677000	-0.303530000
6	3.654951000	1.200018000	-0.125267000
6	2.506445000	1.954347000	-0.037886000
6	1.226201000	1.389046000	-0.107788000
6	1.187193000	-0.004720000	-0.275649000
6	-0.000031000	2.111351000	-0.013646000
6	-1.226262000	1.389038000	-0.107800000
6	-1.187242000	-0.004720000	-0.275695000
6	-2.506510000	1.954331000	-0.037862000
6	-3.655005000	1.199992000	-0.125271000
6	-2.319934000	-0.776662000	-0.364496000
6	-3.598095000	-0.194687000	-0.303644000
1	-2.601500000	3.024278000	0.095161000
1	-2.180542000	-1.839174000	-0.481541000
1	-4.604119000	1.706041000	-0.052755000
1	2.180509000	-1.839175000	-0.481397000
1	2.601431000	3.024305000	0.095058000
1	4.604054000	1.706099000	-0.052820000

Xanthione

8	-0.000022000	-0.679810000	-0.350710000
16	-0.000037000	3.826385000	0.223219000
7	4.735157000	-0.956498000	-0.432765000
7	-4.735198000	-0.956512000	-0.432999000
6	4.654253000	-2.402859000	-0.537726000
1	3.875169000	-2.665706000	-1.255517000
1	5.590998000	-2.746400000	-0.975917000
6	4.413798000	-3.127361000	0.782984000
1	3.504711000	-2.773853000	1.268651000
1	5.244008000	-2.972434000	1.471474000
1	4.312137000	-4.200270000	0.611109000
6	6.051237000	-0.356065000	-0.302019000
1	6.759849000	-1.025691000	-0.788958000
1	6.077591000	0.573102000	-0.873589000
6	6.497068000	-0.099345000	1.133893000
1	6.592553000	-1.032958000	1.687579000
1	5.783570000	0.532494000	1.662120000
1	7.467115000	0.400593000	1.143924000
6	-4.654227000	-2.402884000	-0.537661000
1	-5.591005000	-2.746580000	-0.975667000
1	-3.875220000	-2.665824000	-1.255506000
6	-4.413486000	-3.127120000	0.783149000
1	-5.243581000	-2.972150000	1.471763000
1	-3.504348000	-2.773430000	1.268588000
1	-4.311737000	-4.200049000	0.611457000
6	-6.051261000	-0.356120000	-0.302012000
1	-6.077655000	0.573135000	-0.873436000
1	-6.759910000	-1.025649000	-0.789029000
6	-6.497027000	-0.099641000	1.133960000
1	-5.783472000	0.532028000	1.662312000
1	-6.592618000	-1.033350000	1.687469000
1	-7.467030000	0.400383000	1.144092000

Xanthione

3. L_b

6	2.318980000	-0.798951000	-0.366953000
6	3.595585000	-0.197901000	-0.298834000
6	3.643688000	1.201412000	-0.117232000
6	2.498357000	1.953831000	-0.030951000
6	1.216149000	1.369874000	-0.108044000
6	1.187932000	-0.034692000	-0.280116000
6	0.000011000	2.077846000	-0.020513000
6	-1.216192000	1.369873000	-0.108161000
6	-1.187999000	-0.034603000	-0.280179000
6	-2.498346000	1.953816000	-0.031175000
6	-3.643700000	1.201391000	-0.117476000
6	-2.319040000	-0.798929000	-0.367015000
6	-3.595605000	-0.197911000	-0.298980000
1	-2.553430000	3.023543000	0.106664000
1	-2.192266000	-1.862363000	-0.486775000
1	-4.592593000	1.707172000	-0.040233000
1	2.192240000	-1.862384000	-0.486751000
1	2.553374000	3.023551000	0.106931000
1	4.592584000	1.707168000	-0.039879000
8	-0.000009000	-0.697704000	-0.359100000
16	0.000188000	3.842376000	0.211046000
7	4.733134000	-0.947359000	-0.420021000
7	-4.733201000	-0.947394000	-0.420298000
6	4.668787000	-2.396006000	-0.535373000
1	3.893816000	-2.662444000	-1.255364000
1	5.610394000	-2.724857000	-0.973115000
6	4.435261000	-3.126115000	0.783037000
1	3.518853000	-2.788384000	1.265938000

Xanthione

1	5.260663000	-2.960339000	1.474478000
1	4.351542000	-4.199622000	0.606504000
6	6.048174000	-0.335838000	-0.302553000
1	6.756193000	-1.001861000	-0.794250000
1	6.060616000	0.592298000	-0.875038000
6	6.499764000	-0.076674000	1.130592000
1	6.604374000	-1.009273000	1.684097000
1	5.786634000	0.552454000	1.662236000
1	7.467015000	0.428208000	1.132594000
6	-4.668790000	-2.396096000	-0.534884000
1	-5.610371000	-2.725214000	-0.972481000
1	-3.893802000	-2.662855000	-1.254753000
6	-4.435198000	-3.125559000	0.783875000
1	-5.260634000	-2.959578000	1.475226000
1	-3.518855000	-2.787461000	1.266644000
1	-4.351303000	-4.199138000	0.607860000
6	-6.048243000	-0.335905000	-0.302806000
1	-6.060676000	0.592255000	-0.875239000
1	-6.756258000	-1.001896000	-0.794563000
6	-6.499912000	-0.076746000	1.130318000
1	-5.786817000	0.552390000	1.662005000
1	-6.604571000	-1.009330000	1.683843000
1	-7.467163000	0.428145000	1.132257000

B. S=2

1. S_0

6	2.335613000	0.807924000	-0.884367000
6	3.608222000	1.033142000	-0.334146000
6	3.723906000	0.992722000	1.086807000

Xanthione

6	2.635940000	0.721580000	1.878475000
6	1.357106000	0.461920000	1.341795000
6	1.261972000	0.526678000	-0.059765000
6	0.213705000	0.104864000	2.145832000
6	-1.012832000	-0.127028000	1.426255000
6	-1.056395000	-0.029388000	0.023706000
6	-2.230353000	-0.472715000	2.050583000
6	-3.385123000	-0.686003000	1.339920000
6	-2.197785000	-0.249603000	-0.720706000
6	-3.407832000	-0.560219000	-0.080838000
1	-2.228725000	-0.566917000	3.128693000
1	-2.116800000	-0.142147000	-1.789697000
1	-4.283379000	-0.950453000	1.877547000
1	2.154561000	0.800091000	-1.947209000
1	2.740661000	0.684614000	2.954843000
1	4.678376000	1.166765000	1.560434000
8	0.069285000	0.287565000	-0.705171000
16	0.315796000	-0.047655000	3.865677000
7	4.699783000	1.285983000	-1.132196000
7	-4.570736000	-0.722673000	-0.795208000
6	4.575708000	1.269956000	-2.592601000
1	3.679221000	1.828901000	-2.879054000
1	5.425074000	1.826256000	-2.996229000
6	4.540757000	-0.136086000	-3.197776000
1	3.727685000	-0.727659000	-2.769625000
1	5.478973000	-0.664880000	-3.009826000
1	4.392248000	-0.080817000	-4.280619000
6	6.042863000	1.419166000	-0.558532000
1	6.659775000	1.931673000	-1.300416000
1	6.000102000	2.081656000	0.311251000
6	6.689024000	0.084252000	-0.179573000
1	6.830302000	-0.545144000	-1.062119000

Xanthione

1	6.066872000	-0.466616000	0.531284000
1	7.667613000	0.250950000	0.280871000
6	-4.564816000	-0.626679000	-2.257747000
1	-5.591544000	-0.426194000	-2.573487000
1	-3.976285000	0.248084000	-2.551050000
6	-4.044631000	-1.884258000	-2.958435000
1	-4.687626000	-2.742679000	-2.746680000
1	-3.032731000	-2.131063000	-2.625693000
1	-4.020691000	-1.734487000	-4.042250000
6	-5.826297000	-1.086451000	-0.132229000
1	-5.940276000	-0.485946000	0.775489000
1	-6.640090000	-0.789240000	-0.798190000
6	-5.944436000	-2.576385000	0.195809000
1	-5.119920000	-2.905517000	0.833957000
1	-5.924914000	-3.178387000	-0.716622000
1	-6.884025000	-2.779257000	0.718865000
6	1.963371000	-2.875289000	-1.618244000
6	2.764232000	-2.765669000	-0.482067000
6	0.590145000	-3.074881000	-1.491016000
1	3.830711000	-2.592302000	-0.587996000
1	-0.036278000	-3.147384000	-2.373257000
6	2.209792000	-2.857869000	0.798335000
6	0.025321000	-3.169043000	-0.219612000
1	-1.044746000	-3.304509000	-0.108015000
6	0.829389000	-3.061603000	0.912340000
1	0.381284000	-3.111761000	1.899118000
1	2.411894000	-2.798767000	-2.603181000
6	-1.185620000	3.332887000	-0.540293000
6	-1.868225000	3.236546000	-1.751434000
6	-1.862349000	3.211541000	0.677596000
1	-1.323312000	3.328078000	-2.684726000
6	-3.244509000	3.017563000	-1.764036000

Xanthione

6	-3.243270000	2.981667000	0.651020000
1	-3.778490000	2.947739000	-2.705619000
1	-3.778918000	2.866217000	1.587626000
6	-3.930308000	2.887620000	-0.556171000
1	-4.999191000	2.703464000	-0.555468000
1	-0.111978000	3.491514000	-0.537015000
6	3.069644000	-2.745869000	2.030459000
1	4.000857000	-2.215303000	1.815477000
1	2.548205000	-2.203445000	2.824185000
1	3.331469000	-3.735706000	2.422792000
6	-1.129558000	3.339222000	1.987801000
1	-1.484201000	2.606694000	2.717379000
1	-0.055790000	3.182207000	1.859929000
1	-1.270546000	4.335187000	2.423807000

2. L_a

6	2.240617000	0.771966000	-0.909341000
6	3.507723000	1.070680000	-0.364876000
6	3.609055000	1.131826000	1.043699000
6	2.511573000	0.887750000	1.852437000
6	1.248467000	0.557196000	1.324320000
6	1.166859000	0.523093000	-0.086020000
6	0.093439000	0.230961000	2.090817000
6	-1.127137000	-0.084476000	1.429957000
6	-1.164140000	-0.076170000	0.017628000
6	-2.338115000	-0.414632000	2.068076000
6	-3.495504000	-0.688722000	1.358538000
6	-2.296925000	-0.356029000	-0.708197000
6	-3.513515000	-0.643137000	-0.054440000
1	-2.371534000	-0.452698000	3.150861000

Xanthione

1	-2.215233000	-0.313067000	-1.782175000
1	-4.391254000	-0.931586000	1.910974000
1	2.074120000	0.687055000	-1.971626000
1	2.636869000	0.945215000	2.927793000
1	4.552211000	1.365351000	1.515346000
8	-0.026649000	0.214872000	-0.721820000
16	0.188851000	0.169312000	3.879822000
7	4.600995000	1.302872000	-1.183671000
7	-4.676846000	-0.848937000	-0.775893000
6	4.475168000	1.224166000	-2.639055000
1	3.558607000	1.739039000	-2.944633000
1	5.302769000	1.795571000	-3.068131000
6	4.488961000	-0.202545000	-3.196738000
1	3.698441000	-0.807035000	-2.745190000
1	5.445731000	-0.691120000	-2.992126000
1	4.338176000	-0.190691000	-4.280926000
6	5.940913000	1.449337000	-0.614795000
1	6.571131000	1.901841000	-1.384565000
1	5.908119000	2.167241000	0.211910000
6	6.565027000	0.131904000	-0.144082000
1	6.705313000	-0.553568000	-0.984231000
1	5.925373000	-0.363652000	0.591155000
1	7.541257000	0.312756000	0.316645000
6	-4.666500000	-0.799495000	-2.237970000
1	-5.691308000	-0.601559000	-2.563813000
1	-4.071377000	0.061448000	-2.558806000
6	-4.153888000	-2.079842000	-2.903448000
1	-4.802838000	-2.927749000	-2.667742000
1	-3.144530000	-2.323546000	-2.560667000
1	-4.126817000	-1.962188000	-3.991405000
6	-5.920638000	-1.221250000	-0.102996000
1	-6.062358000	-0.579079000	0.773206000

Xanthione

1	-6.741334000	-0.989078000	-0.786737000
6	-5.992197000	-2.695176000	0.305613000
1	-5.156536000	-2.962181000	0.958057000
1	-5.952699000	-3.343964000	-0.573590000
1	-6.924561000	-2.900467000	0.841043000
6	1.986827000	-2.936694000	-1.522382000
6	3.014124000	-2.809653000	-0.587283000
6	0.664652000	-3.041614000	-1.098468000
1	4.042273000	-2.719284000	-0.923567000
1	-0.138178000	-3.126372000	-1.822243000
6	2.738558000	-2.783243000	0.782108000
6	0.376859000	-3.022473000	0.266298000
1	-0.650706000	-3.088639000	0.605324000
6	1.405176000	-2.893229000	1.195486000
1	1.171679000	-2.859585000	2.254899000
1	2.221167000	-2.949811000	-2.581639000
6	-1.135777000	3.276124000	-0.536856000
6	-1.673246000	3.127341000	-1.813912000
6	-1.948752000	3.198312000	0.598017000
1	-1.022844000	3.179619000	-2.680126000
6	-3.038329000	2.900004000	-1.976608000
6	-3.317330000	2.961874000	0.421469000
1	-3.458879000	2.784729000	-2.969839000
1	-3.957550000	2.877434000	1.293624000
6	-3.859715000	2.816098000	-0.852059000
1	-4.920833000	2.623228000	-0.967141000
1	-0.069281000	3.436209000	-0.415944000
6	3.841843000	-2.617292000	1.794945000
1	4.826065000	-2.680335000	1.322648000
1	3.769965000	-1.642599000	2.287954000
1	3.790494000	-3.385146000	2.573549000
6	-1.371393000	3.374089000	1.978470000

Xanthione

1	-1.803617000	2.657394000	2.681788000
1	-0.289479000	3.222771000	1.978072000
1	-1.569931000	4.380266000	2.366253000

3. L_b

6	2.586476000	-0.079504000	-1.078892000
6	3.869191000	0.108766000	-0.515796000
6	3.930688000	0.540828000	0.832819000
6	2.783890000	0.775983000	1.567549000
6	1.493992000	0.589930000	1.021085000
6	1.458604000	0.157769000	-0.327555000
6	0.279583000	0.783884000	1.722345000
6	-0.947807000	0.533873000	1.072608000
6	-0.947269000	0.109884000	-0.285040000
6	-2.226875000	0.659344000	1.679685000
6	-3.386978000	0.386691000	0.990313000
6	-2.090721000	-0.158565000	-0.988285000
6	-3.357878000	-0.022508000	-0.369353000
1	-2.260182000	0.976068000	2.712920000
1	-1.980491000	-0.487977000	-2.008734000
1	-4.330064000	0.495138000	1.505587000
1	2.444550000	-0.417240000	-2.093666000
1	2.851712000	1.103142000	2.596338000
1	4.886572000	0.687835000	1.313890000
8	0.245758000	-0.060559000	-0.962362000
16	0.273456000	1.344543000	3.472142000
7	5.013154000	-0.112574000	-1.261450000
7	-4.515406000	-0.275118000	-1.070016000
6	4.927411000	-0.650772000	-2.619771000
1	4.160623000	-0.101824000	-3.176823000

Xanthione

1	5.877336000	-0.433754000	-3.114679000
6	4.647309000	-2.155463000	-2.679215000
1	3.722251000	-2.404546000	-2.152154000
1	5.460520000	-2.721188000	-2.216968000
1	4.548814000	-2.485009000	-3.718282000
6	6.341189000	0.047163000	-0.666866000
1	7.047788000	0.195921000	-1.487658000
1	6.360331000	0.969106000	-0.077079000
6	6.789072000	-1.139326000	0.191149000
1	6.874430000	-2.046721000	-0.412811000
1	6.073393000	-1.335022000	0.994084000
1	7.765133000	-0.937504000	0.643386000
6	-4.473317000	-0.655700000	-2.484261000
1	-5.439979000	-0.392047000	-2.920786000
1	-3.726746000	-0.038435000	-2.992831000
6	-4.186658000	-2.141455000	-2.718832000
1	-4.983788000	-2.763177000	-2.302388000
1	-3.245789000	-2.436829000	-2.247960000
1	-4.116635000	-2.350897000	-3.790654000
6	-5.815384000	-0.296463000	-0.392956000
1	-5.916647000	0.601921000	0.224593000
1	-6.583113000	-0.225898000	-1.166631000
6	-6.044705000	-1.551577000	0.453697000
1	-5.260961000	-1.664262000	1.207426000
1	-6.044418000	-2.448111000	-0.171849000
1	-7.008759000	-1.493222000	0.967946000
6	0.304538000	-3.263938000	-0.287851000
6	0.840725000	-2.856294000	0.931765000
6	-1.067811000	-3.466728000	-0.415883000
1	1.909139000	-2.693574000	1.025381000
1	-1.485063000	-3.790357000	-1.363063000
6	0.017566000	-2.644539000	2.044753000

Xanthione

6	-1.902631000	-3.246476000	0.681264000
1	-2.973892000	-3.386857000	0.584706000
6	-1.364325000	-2.838196000	1.896940000
1	-2.016719000	-2.662579000	2.745881000
1	0.959515000	-3.416727000	-1.138559000
6	0.453609000	3.290234000	-1.137989000
6	-0.261740000	2.916358000	-2.274178000
6	-0.206271000	3.731587000	0.014827000
1	0.269624000	2.570252000	-3.153660000
6	-1.653554000	2.975566000	-2.275650000
6	-1.608829000	3.775968000	0.002338000
1	-2.212244000	2.682495000	-3.157754000
1	-2.135343000	4.099233000	0.894308000
6	-2.325763000	3.404342000	-1.129549000
1	-3.409609000	3.437446000	-1.116807000
1	1.536918000	3.233474000	-1.138414000
6	0.594012000	-2.215021000	3.360888000
1	1.682779000	-2.307507000	3.369973000
1	0.359443000	-1.158620000	3.577560000
1	0.188502000	-2.804403000	4.189363000
6	0.559096000	4.143715000	1.237610000
1	0.357045000	3.466952000	2.084430000
1	1.637060000	4.123697000	1.060054000
1	0.280765000	5.151067000	1.565000000

X. OPTIMISED GEOMETRIES - WATER

A. Implicit

1. S_0

6	2.308060000	-0.785900000	-0.357224000

Xanthione

6	3.582024000	-0.205367000	-0.292609000
6	3.639086000	1.206128000	-0.110782000
6	2.502370000	1.952139000	-0.029646000
6	1.214847000	1.387537000	-0.105187000
6	1.175886000	-0.003185000	-0.271774000
6	0.000001000	2.152539000	-0.017139000
6	-1.214822000	1.387531000	-0.105183000
6	-1.175853000	-0.003193000	-0.271748000
6	-2.502360000	1.952125000	-0.029562000
6	-3.639064000	1.206108000	-0.110659000
6	-2.308023000	-0.785923000	-0.357184000
6	-3.581966000	-0.205388000	-0.292499000
1	-2.571735000	3.022145000	0.104222000
1	-2.166182000	-1.847973000	-0.471138000
1	-4.590444000	1.706546000	-0.034241000
1	2.166204000	-1.847944000	-0.471166000
1	2.571748000	3.022161000	0.104121000
1	4.590443000	1.706622000	-0.034476000
8	0.000016000	-0.676315000	-0.351968000
16	0.000001000	3.832855000	0.176128000
7	4.712368000	-0.948233000	-0.411372000
7	-4.712278000	-0.948240000	-0.411194000
6	4.642306000	-2.396876000	-0.562494000
1	3.860154000	-2.639666000	-1.282373000
1	5.578833000	-2.717098000	-1.015289000
6	4.416920000	-3.154505000	0.740055000
1	3.505168000	-2.827249000	1.239231000
1	5.250200000	-3.008801000	1.426363000
1	4.327715000	-4.222502000	0.537369000
6	6.034463000	-0.345982000	-0.273057000
1	6.740588000	-1.017433000	-0.757919000
1	6.065719000	0.584041000	-0.840441000

Xanthione

6	6.465269000	-0.102702000	1.167649000
1	6.536324000	-1.039668000	1.718795000
1	5.761160000	0.544945000	1.689726000
1	7.444712000	0.376985000	1.186197000
6	-4.642225000	-2.396831000	-0.562616000
1	-5.578608000	-2.716943000	-1.015790000
1	-3.859839000	-2.639513000	-1.282280000
6	-4.417287000	-3.154639000	0.739897000
1	-5.250745000	-3.008902000	1.425991000
1	-3.505636000	-2.827496000	1.239339000
1	-4.328138000	-4.222628000	0.537142000
6	-6.034385000	-0.345972000	-0.273076000
1	-6.065615000	0.583955000	-0.840638000
1	-6.740447000	-1.017537000	-0.757872000
6	-6.465265000	-0.102475000	1.167561000
1	-5.761301000	0.545421000	1.689525000
1	-6.536128000	-1.039336000	1.718915000
1	-7.444808000	0.377014000	1.186005000

2. *L_a*

6	2.322782000	-0.779263000	-0.359562000
6	3.602608000	-0.195308000	-0.306515000
6	3.658300000	1.202397000	-0.142404000
6	2.507828000	1.956072000	-0.060672000
6	1.229227000	1.387234000	-0.121751000
6	1.189533000	-0.007163000	-0.275235000
6	0.000008000	2.111633000	-0.027984000
6	-1.229229000	1.387267000	-0.121746000
6	-1.189566000	-0.007137000	-0.275256000
6	-2.507811000	1.956134000	-0.060657000

Xanthione

6	-3.658309000	1.202490000	-0.142448000
6	-2.322834000	-0.779199000	-0.359615000
6	-3.602651000	-0.195201000	-0.306609000
1	-2.601079000	3.029042000	0.058495000
1	-2.186518000	-1.843445000	-0.465620000
1	-4.606867000	1.710734000	-0.079243000
1	2.186417000	-1.843500000	-0.465587000
1	2.601114000	3.028980000	0.058467000
1	4.606870000	1.710608000	-0.079144000
8	-0.000024000	-0.684412000	-0.339154000
16	0.000025000	3.810440000	0.253114000
7	4.737645000	-0.955396000	-0.432108000
7	-4.737719000	-0.955279000	-0.432260000
6	4.659111000	-2.404050000	-0.534496000
1	3.876665000	-2.670056000	-1.246976000
1	5.594212000	-2.745495000	-0.976276000
6	4.428658000	-3.125594000	0.788995000
1	3.518965000	-2.777841000	1.278298000
1	5.262346000	-2.965315000	1.471859000
1	4.332199000	-4.198865000	0.618073000
6	6.055461000	-0.353831000	-0.300201000
1	6.764822000	-1.029803000	-0.775702000
1	6.086537000	0.570040000	-0.879751000
6	6.492755000	-0.085009000	1.135591000
1	6.577811000	-1.013539000	1.699257000
1	5.782825000	0.560673000	1.652215000
1	7.466562000	0.407017000	1.144134000
6	-4.659186000	-2.403919000	-0.534761000
1	-5.594296000	-2.745341000	-0.976556000
1	-3.876754000	-2.669830000	-1.247291000
6	-4.428656000	-3.125672000	0.788608000
1	-5.262296000	-2.965538000	1.471563000

Xanthione

1	-3.518930000	-2.778041000	1.277941000
1	-4.332226000	-4.198908000	0.617452000
6	-6.055517000	-0.353769000	-0.299928000
1	-6.086727000	0.570229000	-0.879266000
1	-6.765000000	-1.029618000	-0.775428000
6	-6.492519000	-0.085209000	1.136003000
1	-5.782463000	0.560354000	1.652598000
1	-6.577517000	-1.013834000	1.699521000
1	-7.466307000	0.406853000	1.144811000

3. L_b

6	2.322413000	-0.777232000	-0.373372000
6	3.600906000	-0.191449000	-0.296271000
6	3.647076000	1.208357000	-0.109962000
6	2.497019000	1.954555000	-0.030354000
6	1.214058000	1.388527000	-0.115784000
6	1.192233000	-0.003340000	-0.291266000
6	-0.008673000	2.133738000	-0.028434000
6	-1.215678000	1.386189000	-0.111574000
6	-1.177649000	-0.032645000	-0.293393000
6	-2.517633000	1.957541000	-0.022890000
6	-3.653453000	1.206613000	-0.109282000
6	-2.299062000	-0.794546000	-0.381540000
6	-3.593241000	-0.202405000	-0.296863000
1	-2.577529000	3.025558000	0.119314000
1	-2.169848000	-1.856222000	-0.512690000
1	-4.606519000	1.703926000	-0.030826000
1	2.187196000	-1.839564000	-0.497220000
1	2.563595000	3.024086000	0.110424000
1	4.594011000	1.717000000	-0.025125000

Xanthione

8	0.005670000	-0.693205000	-0.385167000
16	-0.065058000	3.859856000	0.182434000
7	4.737073000	-0.943693000	-0.413757000
7	-4.708306000	-0.955741000	-0.399354000
6	4.666014000	-2.391319000	-0.545823000
1	3.893468000	-2.645236000	-1.272937000
1	5.608458000	-2.720178000	-0.980701000
6	4.420591000	-3.135615000	0.762244000
1	3.501924000	-2.801133000	1.243715000
1	5.243661000	-2.983010000	1.459382000
1	4.332756000	-4.205906000	0.570210000
6	6.053268000	-0.339590000	-0.265905000
1	6.766947000	-1.006376000	-0.747234000
1	6.083237000	0.593260000	-0.830093000
6	6.477728000	-0.093595000	1.177715000
1	6.558786000	-1.030784000	1.727252000
1	5.763305000	0.544083000	1.697897000
1	7.451209000	0.398300000	1.200532000
6	-4.637941000	-2.406448000	-0.566381000
1	-5.579033000	-2.718908000	-1.012272000
1	-3.859626000	-2.640495000	-1.290720000
6	-4.404190000	-3.164578000	0.735454000
1	-5.228633000	-3.015972000	1.430692000
1	-3.483008000	-2.845860000	1.221809000
1	-4.327288000	-4.230904000	0.521952000
6	-6.037827000	-0.364953000	-0.255351000
1	-6.073256000	0.563990000	-0.822744000
1	-6.739178000	-1.046118000	-0.730834000
6	-6.449704000	-0.124185000	1.192707000
1	-5.744324000	0.529632000	1.704030000
1	-6.512222000	-1.060273000	1.745042000
1	-7.431430000	0.349358000	1.213260000

Xanthione

B. S=2

1. S_0

6	1.959811000	-0.942290000	-0.342665000
6	3.279097000	-0.447435000	-0.278146000
6	3.444361000	0.965646000	-0.125227000
6	2.358082000	1.798867000	-0.056759000
6	1.024633000	1.326239000	-0.123711000
6	0.895250000	-0.068108000	-0.270107000
6	-0.135533000	2.172068000	-0.045509000
6	-1.416378000	1.516964000	-0.121248000
6	-1.506699000	0.120088000	-0.266746000
6	-2.656903000	2.192541000	-0.048245000
6	-3.858885000	1.535537000	-0.113961000
6	-2.695403000	-0.577481000	-0.336634000
6	-3.920818000	0.116054000	-0.270044000
1	-2.636447000	3.267917000	0.067968000
1	-2.640601000	-1.650774000	-0.435916000
1	-4.767903000	2.112921000	-0.040640000
1	1.738019000	-1.994017000	-0.440271000
1	2.505962000	2.864938000	0.055019000
1	4.435179000	1.397100000	-0.070703000
8	-0.359492000	-0.652796000	-0.343601000
16	-0.001869000	3.899676000	0.133404000
7	4.356669000	-1.281666000	-0.366212000
7	-5.116374000	-0.540325000	-0.361201000
6	4.174684000	-2.733503000	-0.498371000
1	3.392842000	-2.927792000	-1.239017000
1	5.099832000	-3.136100000	-0.915300000

Xanthione

6	3.849626000	-3.441315000	0.818750000
1	2.946181000	-3.032417000	1.279112000
1	4.671214000	-3.334960000	1.531452000
1	3.687784000	-4.508564000	0.641160000
6	5.730407000	-0.774620000	-0.216647000
1	6.390974000	-1.499526000	-0.697114000
1	5.838695000	0.166030000	-0.759307000
6	6.150321000	-0.568821000	1.239121000
1	6.145865000	-1.511540000	1.792438000
1	5.474727000	0.126751000	1.745364000
1	7.161314000	-0.152677000	1.279726000
6	-5.162993000	-2.004248000	-0.481775000
1	-6.137246000	-2.261477000	-0.901566000
1	-4.417608000	-2.324994000	-1.215854000
6	-4.959552000	-2.739844000	0.844011000
1	-5.756724000	-2.495840000	1.550742000
1	-4.004337000	-2.474391000	1.304983000
1	-4.968730000	-3.821139000	0.678644000
6	-6.393621000	0.177947000	-0.243032000
1	-6.346203000	1.095440000	-0.836214000
1	-7.156477000	-0.448138000	-0.709760000
6	-6.792001000	0.492022000	1.199734000
1	-6.028693000	1.093943000	1.700487000
1	-6.930055000	-0.427215000	1.774726000
1	-7.732521000	1.050441000	1.217523000
8	-0.594176000	-3.422340000	-0.496509000
1	-0.615293000	-3.728177000	0.426781000
1	-0.505346000	-2.446702000	-0.504019000
8	6.419941000	2.437443000	-0.082800000
1	6.514882000	2.482151000	0.885207000
1	6.053434000	3.281627000	-0.401669000

Xanthione

2. L_a

6	2.161097000	-1.043598000	-0.344579000
6	3.478868000	-0.534696000	-0.287463000
6	3.621744000	0.867160000	-0.151212000
6	2.522062000	1.708038000	-0.093585000
6	1.202447000	1.213998000	-0.158916000
6	1.086806000	-0.185189000	-0.285128000
6	0.015137000	2.008642000	-0.096801000
6	-1.267532000	1.378111000	-0.156335000
6	-1.344147000	-0.023539000	-0.281773000
6	-2.508705000	2.042424000	-0.088550000
6	-3.715392000	1.364525000	-0.141623000
6	-2.526038000	-0.726698000	-0.336868000
6	-3.762970000	-0.043775000	-0.276269000
1	-2.525353000	3.121904000	0.014257000
1	-2.463763000	-1.801358000	-0.422891000
1	-4.627660000	1.938250000	-0.073186000
1	1.952589000	-2.099640000	-0.430560000
1	2.686977000	2.776910000	0.009170000
1	4.604826000	1.309997000	-0.085197000
8	-0.175354000	-0.788112000	-0.352354000
16	0.103374000	3.783606000	0.064886000
7	4.570863000	-1.375625000	-0.376311000
7	-4.958956000	-0.728753000	-0.360322000
6	4.394500000	-2.827692000	-0.465455000
1	3.609358000	-3.050212000	-1.195686000
1	5.318727000	-3.240426000	-0.876397000
6	4.079528000	-3.504062000	0.871383000
1	3.175492000	-3.087176000	1.323764000
1	4.903459000	-3.371508000	1.577523000
1	3.923479000	-4.577306000	0.725577000

Xanthione

6	5.934552000	-0.852416000	-0.258525000
1	6.602968000	-1.589573000	-0.709571000
1	6.025417000	0.054455000	-0.865073000
6	6.373242000	-0.572009000	1.180875000
1	6.381985000	-1.492406000	1.770812000
1	5.697973000	0.136718000	1.668176000
1	7.381804000	-0.147595000	1.194870000
6	-4.981530000	-2.191977000	-0.441325000
1	-5.954332000	-2.477665000	-0.847902000
1	-4.236113000	-2.523264000	-1.171866000
6	-4.757659000	-2.896582000	0.899182000
1	-5.554230000	-2.649156000	1.605787000
1	-3.804269000	-2.603395000	1.347339000
1	-4.748947000	-3.981903000	0.759906000
6	-6.238658000	-0.024528000	-0.242262000
1	-6.205985000	0.884704000	-0.851201000
1	-7.001864000	-0.664627000	-0.690958000
6	-6.633311000	0.316110000	1.196912000
1	-5.867371000	0.926923000	1.682717000
1	-6.767103000	-0.593244000	1.788828000
1	-7.574473000	0.874214000	1.210636000
8	-0.364885000	-3.532669000	-0.465129000
1	-0.380632000	-3.829887000	0.460943000
1	-0.295685000	-2.553121000	-0.481837000
8	2.901220000	4.934025000	0.191612000
1	2.534257000	5.316646000	1.008352000
1	2.579045000	5.446266000	-0.571194000

3. L_b

6	1.949570000	-0.926084000	-0.363734000

Xanthione

6	3.279688000	-0.412291000	-0.277724000
6	3.436229000	0.999397000	-0.107313000
6	2.340502000	1.823309000	-0.033571000
6	0.999574000	1.336935000	-0.116802000
6	0.883363000	-0.076920000	-0.282198000
6	-0.165768000	2.169168000	-0.043096000
6	-1.438919000	1.511428000	-0.121763000
6	-1.532720000	0.109085000	-0.277771000
6	-2.686827000	2.177376000	-0.042834000
6	-3.892105000	1.513795000	-0.113674000
6	-2.714622000	-0.587194000	-0.350560000
6	-3.950759000	0.101617000	-0.277476000
1	-2.669753000	3.251714000	0.082195000
1	-2.660427000	-1.660098000	-0.458585000
1	-4.801999000	2.090543000	-0.037050000
1	1.743788000	-1.979589000	-0.483716000
1	2.468402000	2.889394000	0.091265000
1	4.425063000	1.439155000	-0.047746000
8	-0.374012000	-0.673831000	-0.365761000
16	-0.004047000	3.938218000	0.146134000
7	4.351398000	-1.247864000	-0.357392000
7	-5.145837000	-0.568664000	-0.373230000
6	4.178246000	-2.699557000	-0.530999000
1	3.409268000	-2.877408000	-1.287037000
1	5.114407000	-3.084333000	-0.937163000
6	3.833847000	-3.435111000	0.767962000
1	2.906084000	-3.058863000	1.206156000
1	4.632067000	-3.324302000	1.505164000
1	3.705796000	-4.500902000	0.559519000
6	5.724768000	-0.745514000	-0.176539000
1	6.392760000	-1.469617000	-0.644986000
1	5.841610000	0.203727000	-0.701696000

Xanthione

6	6.102543000	-0.552292000	1.294727000
1	6.088537000	-1.498388000	1.840885000
1	5.414883000	0.141725000	1.784983000
1	7.110926000	-0.133940000	1.355705000
6	-5.184234000	-2.031351000	-0.490179000
1	-6.157124000	-2.295601000	-0.909025000
1	-4.436225000	-2.351591000	-1.221878000
6	-4.977215000	-2.763115000	0.838599000
1	-5.776635000	-2.522938000	1.543968000
1	-4.024054000	-2.489227000	1.298794000
1	-4.979092000	-3.844700000	0.674627000
6	-6.424376000	0.142705000	-0.253622000
1	-6.378274000	1.062852000	-0.843407000
1	-7.186253000	-0.484710000	-0.720456000
6	-6.824130000	0.454559000	1.190751000
1	-6.059041000	1.052808000	1.692776000
1	-6.966585000	-0.465499000	1.763220000
1	-7.762854000	1.016088000	1.207085000
8	-0.184810000	-3.436429000	-0.421723000
1	-0.163177000	-3.731815000	0.505050000
1	-0.373129000	-2.473656000	-0.444499000
8	6.367431000	2.399816000	-0.116118000
1	6.542711000	2.604202000	0.819459000
1	6.135343000	3.223051000	-0.581672000

C. S=4

1. S_0

6	-2.402083000	-0.820067000	-0.271963000
6	-3.691131000	-0.249217000	-0.242878000

Xanthione

6	-3.779772000	1.174195000	-0.138014000
6	-2.646192000	1.944642000	-0.083363000
6	-1.341550000	1.395593000	-0.115414000
6	-1.288894000	-0.007000000	-0.209037000
6	-0.134814000	2.177599000	-0.053700000
6	1.105897000	1.453480000	-0.103433000
6	1.125689000	0.047159000	-0.194246000
6	2.381114000	2.067900000	-0.068499000
6	3.550005000	1.353652000	-0.110383000
6	2.276185000	-0.711228000	-0.246364000
6	3.535938000	-0.074104000	-0.201810000
1	2.413455000	3.147375000	-0.002060000
1	2.183241000	-1.783162000	-0.344427000
1	4.488759000	1.890539000	-0.081766000
1	-2.244915000	-1.885728000	-0.334618000
1	-2.732941000	3.020777000	-0.009958000
1	-4.742091000	1.670770000	-0.105923000
8	-0.066947000	-0.665959000	-0.229327000
16	-0.178592000	3.916765000	0.069630000
7	-4.812162000	-1.027994000	-0.321348000
7	4.695475000	-0.792441000	-0.248612000
6	-6.156740000	-0.443123000	-0.212586000
1	-6.203221000	0.476569000	-0.798895000
1	-6.850867000	-1.149185000	-0.672896000
6	-6.583382000	-0.152293000	1.226517000
1	-5.896345000	0.551855000	1.703701000
1	-6.606117000	-1.066666000	1.824784000
1	-7.586779000	0.284775000	1.240084000
6	-4.708154000	-2.490806000	-0.408981000
1	-5.650243000	-2.855376000	-0.822973000
1	-3.932392000	-2.749105000	-1.136501000
6	-4.432746000	-3.177575000	0.930475000

Xanthione

1	-5.250149000	-3.001277000	1.634121000
1	-3.508415000	-2.811355000	1.385267000
1	-4.334962000	-4.257388000	0.784703000
6	6.006474000	-0.130209000	-0.162398000
1	6.733333000	-0.803234000	-0.622818000
1	5.997052000	0.785081000	-0.756410000
6	6.435778000	0.201752000	1.267543000
1	6.553952000	-0.703101000	1.869150000
1	5.697505000	0.843052000	1.757680000
1	7.393016000	0.731445000	1.255167000
6	4.673628000	-2.263950000	-0.237320000
1	3.909324000	-2.633150000	-0.925328000
1	5.639454000	-2.598615000	-0.621859000
6	4.427487000	-2.864821000	1.148169000
1	3.477082000	-2.517630000	1.563284000
1	5.224073000	-2.597344000	1.847113000
1	4.384983000	-3.955508000	1.076707000
8	-6.502843000	2.911506000	-0.163790000
1	-6.922616000	2.979177000	-1.039397000
1	-7.183671000	2.692727000	0.496408000
8	6.364715000	3.147421000	-0.225415000
1	5.877943000	3.910437000	-0.585291000
1	6.464217000	3.264173000	0.736121000
8	2.037632000	-4.006306000	-1.326336000
1	1.908755000	-3.597205000	-2.199298000
1	1.218766000	-3.866346000	-0.773633000
8	-0.087500000	-3.368618000	0.114757000
1	-0.103483000	-2.385070000	0.080845000
1	-0.218305000	-3.691324000	1.021564000

Xanthione

2. *L*$_a$

6	-2.417795000	-0.817457000	-0.264956000
6	-3.709717000	-0.242413000	-0.253032000
6	-3.794191000	1.169224000	-0.169451000
6	-2.649407000	1.946330000	-0.115722000
6	-1.352950000	1.391802000	-0.129658000
6	-1.302008000	-0.013340000	-0.204705000
6	-0.132585000	2.132600000	-0.064248000
6	1.121001000	1.448765000	-0.121034000
6	1.140609000	0.040925000	-0.193735000
6	2.387583000	2.067214000	-0.106391000
6	3.567918000	1.345564000	-0.149040000
6	2.293071000	-0.709321000	-0.248426000
6	3.555967000	-0.069148000	-0.223899000
1	2.444183000	3.149951000	-0.059886000
1	2.203539000	-1.783309000	-0.329185000
1	4.502963000	1.888866000	-0.135642000
1	-2.265809000	-1.885293000	-0.312691000
1	-2.759709000	3.024602000	-0.062217000
1	-4.752810000	1.673053000	-0.153787000
8	-0.066218000	-0.676714000	-0.202208000
16	-0.174047000	3.903516000	0.137715000
7	-4.838138000	-1.034746000	-0.336053000
7	4.723236000	-0.802092000	-0.283756000
6	-6.176949000	-0.447868000	-0.233619000
1	-6.223273000	0.461024000	-0.838798000
1	-6.876667000	-1.159254000	-0.678637000
6	-6.606289000	-0.125281000	1.199421000
1	-5.910428000	0.576738000	1.667158000
1	-6.641050000	-1.029318000	1.812872000
1	-7.605357000	0.323001000	1.204468000

Xanthione

6	-4.728734000	-2.494789000	-0.391885000
1	-5.665969000	-2.872697000	-0.807056000
1	-3.945689000	-2.768836000	-1.106839000
6	-4.462927000	-3.159505000	0.961774000
1	-5.287360000	-2.973184000	1.654983000
1	-3.544470000	-2.780089000	1.418099000
1	-4.359007000	-4.241787000	0.837733000
6	6.028950000	-0.140064000	-0.199098000
1	6.762128000	-0.817337000	-0.644659000
1	6.022039000	0.765537000	-0.810010000
6	6.456614000	0.218295000	1.226687000
1	6.581462000	-0.677417000	1.840674000
1	5.710276000	0.856988000	1.708429000
1	7.410614000	0.754684000	1.210206000
6	4.694026000	-2.269011000	-0.240817000
1	3.923412000	-2.650197000	-0.915812000
1	5.654802000	-2.618163000	-0.627428000
6	4.457006000	-2.849167000	1.156303000
1	3.514778000	-2.485199000	1.576114000
1	5.263473000	-2.578290000	1.842847000
1	4.402653000	-3.940765000	1.103497000
8	-6.564020000	2.935205000	-0.190762000
1	-7.060060000	2.878811000	-1.026425000
1	-7.144829000	2.657314000	0.539178000
8	6.415443000	3.188253000	-0.199638000
1	5.875129000	3.967643000	-0.421562000
1	6.488853000	3.124513000	0.769125000
8	2.019108000	-4.042469000	-1.292553000
1	1.912968000	-3.599310000	-2.151744000
1	1.211315000	-3.868565000	-0.731688000
8	-0.076182000	-3.342782000	0.157923000
1	-0.095214000	-2.356760000	0.103021000

Xanthione

1	-0.198041000	-3.647415000	1.071963000

3. L_b

6	-2.409758000	-0.815582000	-0.291784000
6	-3.705032000	-0.240077000	-0.261936000
6	-3.787341000	1.177389000	-0.136199000
6	-2.647245000	1.945696000	-0.058614000
6	-1.338478000	1.396755000	-0.087433000
6	-1.299184000	-0.013138000	-0.204796000
6	-0.134825000	2.172387000	-0.006265000
6	1.104267000	1.450290000	-0.068691000
6	1.128282000	0.028543000	-0.183316000
6	2.390558000	2.069489000	-0.035810000
6	3.562347000	1.357328000	-0.103380000
6	2.271511000	-0.715393000	-0.264244000
6	3.545126000	-0.068180000	-0.213016000
1	2.412069000	3.146904000	0.050499000
1	2.189247000	-1.785955000	-0.400701000
1	4.502377000	1.895253000	-0.073435000
1	-2.257295000	-1.881350000	-0.371783000
1	-2.728965000	3.020814000	0.031767000
1	-4.747944000	1.678482000	-0.106236000
8	-0.070347000	-0.690873000	-0.218339000
16	-0.147991000	3.952641000	0.144764000
7	-4.827838000	-1.023193000	-0.358035000
7	4.693192000	-0.799157000	-0.259472000
6	-6.170418000	-0.440748000	-0.233493000
1	-6.218877000	0.488271000	-0.805290000
1	-6.868639000	-1.140769000	-0.696582000
6	-6.586209000	-0.166728000	1.213671000

Xanthione

1	-5.892950000	0.529005000	1.693665000
1	-6.609467000	-1.088378000	1.800398000
1	-7.587598000	0.274320000	1.237011000
6	-4.723964000	-2.482841000	-0.474399000
1	-5.664069000	-2.838893000	-0.900118000
1	-3.942966000	-2.727401000	-1.200820000
6	-4.456535000	-3.196737000	0.853977000
1	-5.276759000	-3.032863000	1.557125000
1	-3.532975000	-2.841760000	1.318907000
1	-4.361210000	-4.273402000	0.685201000
6	6.012876000	-0.147538000	-0.239729000
1	6.706480000	-0.818462000	-0.750543000
1	5.970393000	0.781635000	-0.808125000
6	6.517402000	0.152535000	1.174738000
1	6.677325000	-0.763503000	1.748190000
1	5.804993000	0.781051000	1.715756000
1	7.468469000	0.688501000	1.112373000
6	4.667452000	-2.271704000	-0.195315000
1	3.910051000	-2.668098000	-0.876626000
1	5.641787000	-2.618415000	-0.542294000
6	4.389160000	-2.809027000	1.212554000
1	3.417420000	-2.466457000	1.577802000
1	5.156698000	-2.493778000	1.922975000
1	4.373168000	-3.901935000	1.183602000
8	-6.525438000	2.905988000	-0.169089000
1	-6.948151000	2.983961000	-1.042426000
1	-7.206213000	2.693054000	0.493077000
8	6.351035000	3.060860000	-0.304553000
1	5.968108000	3.842999000	-0.740482000
1	6.561220000	3.285513000	0.619095000
8	2.061957000	-3.910159000	-1.231811000
1	1.881356000	-3.662137000	-2.154985000

Xanthione

1	1.223477000	-3.824715000	-0.694066000
8	-0.104484000	-3.362926000	0.157045000
1	-0.123596000	-2.377212000	0.121506000
1	-0.268336000	-3.689531000	1.056749000

D. S=6

1. S_0

6	-2.324672000	-0.117244000	-0.691789000
6	-3.597158000	0.463879000	-0.503142000
6	-3.631988000	1.866060000	-0.226718000
6	-2.477375000	2.613086000	-0.176114000
6	-1.197155000	2.045441000	-0.355685000
6	-1.192908000	0.662551000	-0.601684000
6	0.037923000	2.788856000	-0.262718000
6	1.253781000	2.028815000	-0.376541000
6	1.231802000	0.635118000	-0.576717000
6	2.546516000	2.592234000	-0.261697000
6	3.687744000	1.835797000	-0.316492000
6	2.351520000	-0.164966000	-0.640114000
6	3.634188000	0.418598000	-0.505642000
1	2.615627000	3.661168000	-0.110576000
1	2.219527000	-1.221769000	-0.823716000
1	4.639993000	2.330849000	-0.204412000
1	-2.199774000	-1.172194000	-0.877080000
1	-2.532697000	3.674167000	0.027095000
1	-4.573814000	2.363692000	-0.056521000
8	0.011489000	-0.037569000	-0.688627000
16	0.045637000	4.505504000	-0.013127000
7	-4.739019000	-0.283819000	-0.547773000

Xanthione

7	4.768120000	-0.333944000	-0.553829000
6	-6.031076000	0.369182000	-0.244951000
1	-6.015572000	0.737778000	0.789432000
1	-6.127248000	1.243926000	-0.895188000
6	-7.257037000	-0.510382000	-0.445013000
1	-7.271255000	-1.369485000	0.230101000
1	-7.336996000	-0.869055000	-1.474469000
1	-8.145851000	0.089352000	-0.232571000
6	-4.650531000	-1.748791000	-0.626104000
1	-5.600181000	-2.127966000	-0.995673000
1	-3.911387000	-2.018246000	-1.383880000
6	-4.314516000	-2.394681000	0.719434000
1	-5.093731000	-2.176103000	1.455611000
1	-3.364941000	-2.025260000	1.115043000
1	-4.246413000	-3.481145000	0.609334000
6	6.093480000	0.275651000	-0.364329000
1	6.823227000	-0.415098000	-0.790591000
1	6.155580000	1.192219000	-0.957606000
6	6.441113000	0.558801000	1.098372000
1	6.474546000	-0.366919000	1.677637000
1	5.706163000	1.222252000	1.562319000
1	7.422019000	1.038569000	1.164024000
6	4.705798000	-1.802973000	-0.655827000
1	3.917617000	-2.099191000	-1.351806000
1	5.651389000	-2.129377000	-1.095045000
6	4.485472000	-2.499439000	0.688985000
1	3.596220000	-2.121595000	1.202778000
1	5.342414000	-2.361400000	1.354509000
1	4.353911000	-3.573846000	0.526723000
8	2.308827000	-3.302660000	-2.301830000
1	2.426830000	-2.806437000	-3.129948000
1	1.360946000	-3.197406000	-1.982805000

Xanthione

8	-0.107523000	-2.804885000	-1.447117000
1	-0.118512000	-1.831658000	-1.389046000
1	-0.447048000	-3.216485000	-0.590096000
8	-0.915335000	-3.607240000	0.870396000
1	-1.708380000	-4.115658000	1.096622000
1	-0.777047000	-2.810909000	1.461698000
8	2.108218000	-1.023022000	2.842423000
1	2.484513000	-0.328070000	2.276207000
1	1.156004000	-1.156767000	2.600428000
8	-0.417619000	-1.260326000	1.874153000
1	-0.175373000	-0.921089000	0.989457000
1	-1.177599000	-0.710712000	2.255267000
8	-2.411194000	0.168522000	2.655579000
1	-2.755669000	0.879811000	2.087669000
1	-3.085371000	-0.129785000	3.287416000

2. L_a

6	-2.336325000	-0.146201000	-0.707211000
6	-3.613436000	0.439891000	-0.535329000
6	-3.646248000	1.830936000	-0.266834000
6	-2.480890000	2.585931000	-0.214203000
6	-1.205394000	2.014119000	-0.383202000
6	-1.203654000	0.625396000	-0.618317000
6	0.038260000	2.712523000	-0.294242000
6	1.268602000	1.993364000	-0.406808000
6	1.248748000	0.597459000	-0.589602000
6	2.554292000	2.562919000	-0.308637000
6	3.706370000	1.800305000	-0.362222000
6	2.371426000	-0.195936000	-0.648837000
6	3.656556000	0.393154000	-0.532994000

Xanthione

1	2.648562000	3.634662000	-0.172208000
1	2.242878000	-1.256889000	-0.813988000
1	4.656117000	2.303505000	-0.262294000
1	-2.214919000	-1.204430000	-0.879533000
1	-2.564014000	3.647715000	-0.012132000
1	-4.585736000	2.335631000	-0.102651000
8	0.013982000	-0.082056000	-0.674536000
16	0.054630000	4.467505000	0.006541000
7	-4.763048000	-0.320952000	-0.593980000
7	4.798947000	-0.370685000	-0.589800000
6	-6.049332000	0.337653000	-0.304111000
1	-6.058508000	0.698388000	0.735296000
1	-6.123447000	1.221576000	-0.945246000
6	-7.278104000	-0.528036000	-0.547546000
1	-7.334449000	-1.378575000	0.136340000
1	-7.310181000	-0.900677000	-1.574979000
1	-8.169906000	0.083064000	-0.385553000
6	-4.664232000	-1.784502000	-0.546494000
1	-5.603892000	-2.200872000	-0.903347000
1	-3.908986000	-2.114117000	-1.263657000
6	-4.352237000	-2.318495000	0.853662000
1	-5.152095000	-2.054801000	1.552400000
1	-3.418551000	-1.901207000	1.240559000
1	-4.266708000	-3.409566000	0.834223000
6	6.119809000	0.245012000	-0.425717000
1	6.849907000	-0.447186000	-0.851140000
1	6.169573000	1.154087000	-1.033380000
6	6.491631000	0.556994000	1.026481000
1	6.550213000	-0.359312000	1.619005000
1	5.753528000	1.214859000	1.493523000
1	7.465698000	1.053723000	1.068569000
6	4.728617000	-1.838516000	-0.630813000

Xanthione

1	3.941078000	-2.159942000	-1.316530000
1	5.673289000	-2.189910000	-1.053881000
6	4.499527000	-2.485435000	0.738375000
1	3.617581000	-2.073095000	1.237782000
1	5.358926000	-2.334907000	1.398175000
1	4.349940000	-3.563145000	0.617841000
8	2.269500000	-3.414416000	-2.187035000
1	2.395199000	-2.927605000	-3.019533000
1	1.326946000	-3.279674000	-1.862649000
8	-0.130276000	-2.838931000	-1.333829000
1	-0.112397000	-1.863055000	-1.300092000
1	-0.481120000	-3.212084000	-0.464999000
8	-0.974661000	-3.526042000	1.014784000
1	-1.807672000	-3.966498000	1.240965000
1	-0.807789000	-2.704393000	1.564321000
8	2.138451000	-0.767831000	2.774288000
1	2.492500000	-0.211938000	2.059044000
1	1.183884000	-0.959534000	2.587675000
8	-0.410587000	-1.148380000	1.907729000
1	-0.170114000	-0.859880000	1.002328000
1	-1.164961000	-0.571296000	2.257329000
8	-2.389646000	0.355173000	2.590699000
1	-2.812319000	0.908949000	1.908801000
1	-2.991448000	0.198485000	3.336221000

3. L_b

6	-2.327736000	-0.112307000	-0.721551000
6	-3.599718000	0.478501000	-0.534040000
6	-3.617804000	1.865519000	-0.220541000
6	-2.449115000	2.603244000	-0.139365000

Xanthione

6	-1.170897000	2.030366000	-0.324480000
6	-1.190799000	0.649228000	-0.603346000
6	0.066539000	2.747374000	-0.215995000
6	1.278772000	1.995656000	-0.357890000
6	1.250120000	0.586787000	-0.581089000
6	2.586436000	2.564664000	-0.270470000
6	3.724918000	1.804628000	-0.359542000
6	2.360241000	-0.200970000	-0.675894000
6	3.659248000	0.389109000	-0.547646000
1	2.650659000	3.631198000	-0.109630000
1	2.239765000	-1.255047000	-0.889635000
1	4.681500000	2.294723000	-0.257476000
1	-2.214251000	-1.165647000	-0.925561000
1	-2.496198000	3.658626000	0.093660000
1	-4.554023000	2.372418000	-0.042669000
8	0.017441000	-0.078878000	-0.678559000
16	0.136629000	4.500991000	0.101057000
7	-4.754891000	-0.265732000	-0.614566000
7	4.776591000	-0.382882000	-0.592947000
6	-6.038622000	0.394309000	-0.311561000
1	-6.044449000	0.725148000	0.737271000
1	-6.104568000	1.296286000	-0.927848000
6	-7.273578000	-0.455238000	-0.577920000
1	-7.331466000	-1.328226000	0.076843000
1	-7.315916000	-0.791373000	-1.617393000
1	-8.159643000	0.156342000	-0.388811000
6	-4.672954000	-1.730556000	-0.651199000
1	-5.617783000	-2.115987000	-1.027853000
1	-3.921351000	-2.026259000	-1.386714000
6	-4.363954000	-2.343270000	0.717362000
1	-5.160528000	-2.111653000	1.430794000
1	-3.425789000	-1.956087000	1.123494000

Xanthione

1	-4.287379000	-3.432005000	0.636449000
6	6.118810000	0.219518000	-0.554148000
1	6.793936000	-0.477350000	-1.053364000
1	6.110642000	1.134540000	-1.149666000
6	6.619135000	0.502107000	0.866056000
1	6.732370000	-0.423247000	1.434689000
1	5.931460000	1.156624000	1.407652000
1	7.594737000	0.993357000	0.816790000
6	4.697805000	-1.856508000	-0.551121000
1	3.924271000	-2.215892000	-1.237415000
1	5.656550000	-2.230301000	-0.913415000
6	4.417872000	-2.398080000	0.856016000
1	3.503088000	-1.979362000	1.286773000
1	5.243428000	-2.181938000	1.539172000
1	4.301261000	-3.484644000	0.798898000
8	2.326566000	-3.226649000	-2.167168000
1	2.421247000	-2.846177000	-3.057493000
1	1.360176000	-3.182633000	-1.883067000
8	-0.125167000	-2.831273000	-1.406449000
1	-0.144253000	-1.856627000	-1.356126000
1	-0.493961000	-3.235178000	-0.557909000
8	-1.010204000	-3.597680000	0.896585000
1	-1.838040000	-4.061372000	1.093202000
1	-0.871687000	-2.786599000	1.469227000
8	1.974471000	-1.071863000	2.966883000
1	2.320489000	-0.216291000	2.662028000
1	1.036226000	-1.172561000	2.659902000
8	-0.489410000	-1.241257000	1.864877000
1	-0.207473000	-0.917725000	0.984204000
1	-1.240525000	-0.661411000	2.219066000
8	-2.438599000	0.278500000	2.586329000
1	-2.794826000	0.920590000	1.945109000

Xanthione

1	-3.104750000	0.044487000	3.252715000

XI. OPTIMISED GEOMETRIES - GAS PHASE

A. Implicit

1. S_0

6	2.310390000	-0.782021000	-0.357465000
6	3.579490000	-0.202048000	-0.297538000
6	3.639592000	1.203287000	-0.118820000
6	2.500645000	1.951657000	-0.033788000
6	1.218819000	1.386778000	-0.104544000
6	1.174907000	0.001779000	-0.269462000
6	0.000022000	2.167007000	-0.009104000
6	-1.218781000	1.386805000	-0.104497000
6	-1.174905000	0.001802000	-0.269444000
6	-2.500600000	1.951695000	-0.033689000
6	-3.639555000	1.203341000	-0.118742000
6	-2.310393000	-0.781980000	-0.357488000
6	-3.579491000	-0.201991000	-0.297557000
1	-2.561435000	3.023125000	0.098522000
1	-2.164861000	-1.843528000	-0.471686000
1	-4.590994000	1.704850000	-0.046809000
1	2.164846000	-1.843570000	-0.471622000
1	2.561474000	3.023096000	0.098362000
1	4.591037000	1.704794000	-0.046991000
8	-0.000005000	-0.677003000	-0.346491000
16	0.000040000	3.819696000	0.192292000
7	4.718271000	-0.953829000	-0.420811000
7	-4.718282000	-0.953745000	-0.420899000
6	4.642354000	-2.398429000	-0.554132000

Xanthione

1	3.860032000	-2.646838000	-1.273316000
1	5.577922000	-2.731080000	-1.003497000
6	4.409417000	-3.146361000	0.754420000
1	3.503950000	-2.800089000	1.251496000
1	5.243593000	-3.004194000	1.440875000
1	4.306324000	-4.216187000	0.565174000
6	6.034648000	-0.351748000	-0.284640000
1	6.744520000	-1.021335000	-0.769906000
1	6.063876000	0.577383000	-0.855180000
6	6.471898000	-0.098888000	1.153931000
1	6.556597000	-1.033094000	1.708431000
1	5.758724000	0.537442000	1.676894000
1	7.445216000	0.394357000	1.171987000
6	-4.642408000	-2.398354000	-0.554210000
1	-5.577956000	-2.730969000	-1.003645000
1	-3.860029000	-2.646774000	-1.273329000
6	-4.409565000	-3.146261000	0.754356000
1	-5.243759000	-3.004076000	1.440790000
1	-3.504112000	-2.800003000	1.251470000
1	-4.306476000	-4.216089000	0.565116000
6	-6.034658000	-0.351668000	-0.284634000
1	-6.063849000	0.577535000	-0.855056000
1	-6.744529000	-1.021190000	-0.769999000
6	-6.471866000	-0.099030000	1.153985000
1	-5.758570000	0.537068000	1.677061000
1	-6.556740000	-1.033337000	1.708293000
1	-7.445097000	0.394387000	1.172170000

2. L_a

6	2.317958000	-0.774817000	-0.366892000

Xanthione

6	3.594822000	-0.193857000	-0.301557000
6	3.652734000	1.198897000	-0.116589000
6	2.505398000	1.954124000	-0.028388000
6	1.224422000	1.390695000	-0.104109000
6	1.185388000	-0.002603000	-0.277292000
6	-0.000003000	2.113796000	-0.012422000
6	-1.224456000	1.390720000	-0.104054000
6	-1.185477000	-0.002541000	-0.277299000
6	-2.505441000	1.954169000	-0.028208000
6	-3.652771000	1.198981000	-0.116485000
6	-2.318070000	-0.774751000	-0.366973000
6	-3.594879000	-0.193769000	-0.301658000
1	-2.600598000	3.022742000	0.111309000
1	-2.176077000	-1.836167000	-0.489854000
1	-4.602168000	1.703956000	-0.039562000
1	2.175998000	-1.836231000	-0.489789000
1	2.600577000	3.022713000	0.110985000
1	4.602134000	1.703887000	-0.039822000
8	-0.000052000	-0.676663000	-0.357840000
16	-0.000010000	3.833410000	0.208615000
7	4.733883000	-0.957417000	-0.432154000
7	-4.733949000	-0.957272000	-0.432447000
6	4.650945000	-2.401809000	-0.540674000
1	3.873763000	-2.661380000	-1.262073000
1	5.588620000	-2.746877000	-0.976768000
6	4.403725000	-3.130117000	0.777113000
1	3.495044000	-2.773064000	1.260620000
1	5.231995000	-2.980010000	1.469111000
1	4.298203000	-4.202525000	0.603351000
6	6.048409000	-0.358290000	-0.298275000
1	6.758256000	-1.024901000	-0.788618000
1	6.074469000	0.573565000	-0.865825000

Xanthione

6	6.494714000	-0.107005000	1.138900000
1	6.593670000	-1.042930000	1.688266000
1	5.777809000	0.518329000	1.670016000
1	7.462937000	0.396724000	1.152859000
6	-4.650925000	-2.401622000	-0.541256000
1	-5.588471000	-2.746653000	-0.977655000
1	-3.873545000	-2.660975000	-1.262530000
6	-4.403921000	-3.130171000	0.776452000
1	-5.232439000	-2.980461000	1.468242000
1	-3.495494000	-2.772889000	1.260276000
1	-4.297994000	-4.202505000	0.602517000
6	-6.048463000	-0.358240000	-0.297875000
1	-6.074848000	0.573716000	-0.865248000
1	-6.758431000	-1.024858000	-0.788011000
6	-6.494138000	-0.107259000	1.139545000
1	-5.777021000	0.518023000	1.670437000
1	-6.592800000	-1.043280000	1.688798000
1	-7.462374000	0.396431000	1.154016000

3. L_b

6	2.320010000	-0.806272000	-0.366532000
6	3.592208000	-0.197081000	-0.299160000
6	3.637391000	1.201100000	-0.116002000
6	2.491174000	1.951793000	-0.028234000
6	1.211509000	1.358282000	-0.105810000
6	1.185745000	-0.045685000	-0.278687000
6	-0.000067000	2.063368000	-0.018424000
6	-1.211567000	1.358109000	-0.105798000
6	-1.185615000	-0.045837000	-0.278589000
6	-2.491311000	1.951426000	-0.028376000

Xanthione

6	-3.637426000	1.200556000	-0.116086000
6	-2.319757000	-0.806611000	-0.366409000
6	-3.592036000	-0.197629000	-0.299111000
1	-2.534692000	3.022108000	0.110258000
1	-2.196696000	-1.870104000	-0.488032000
1	-4.585550000	1.707880000	-0.039132000
1	2.197129000	-1.869812000	-0.487860000
1	2.534338000	3.022478000	0.110434000
1	4.585452000	1.708559000	-0.039219000
8	0.000125000	-0.708284000	-0.356959000
16	-0.000167000	3.839517000	0.211173000
7	4.735048000	-0.943779000	-0.422756000
7	-4.734683000	-0.944603000	-0.422766000
6	4.675673000	-2.391451000	-0.532291000
1	3.902097000	-2.663602000	-1.252106000
1	5.618666000	-2.719830000	-0.968673000
6	4.443317000	-3.118884000	0.788175000
1	3.527037000	-2.779237000	1.269718000
1	5.268048000	-2.949115000	1.479632000
1	4.360756000	-4.193592000	0.617142000
6	6.046147000	-0.326792000	-0.309281000
1	6.756799000	-0.988593000	-0.804095000
1	6.051982000	0.601484000	-0.881977000
6	6.502411000	-0.064240000	1.122114000
1	6.617069000	-0.996104000	1.675195000
1	5.784644000	0.557234000	1.656307000
1	7.465305000	0.449310000	1.122714000
6	-4.675062000	-2.392573000	-0.528395000
1	-5.618583000	-2.722290000	-0.962591000
1	-3.902380000	-2.666620000	-1.248507000
6	-4.440803000	-3.116406000	0.793713000
1	-5.264548000	-2.944782000	1.485873000

Xanthione

1	-3.523849000	-2.775480000	1.273045000
1	-4.358442000	-4.191557000	0.625407000
6	-6.045979000	-0.327494000	-0.312290000
1	-6.050091000	0.600886000	-0.884756000
1	-6.755537000	-0.989016000	-0.809093000
6	-6.505874000	-0.065180000	1.117965000
1	-5.789287000	0.555837000	1.654283000
1	-6.622488000	-0.997126000	1.670510000
1	-7.468569000	0.448755000	1.116136000
